# Serum microRNA signatures as "liquid biopsies" for interrogating hepatotoxic mechanisms and liver pathogenesis in human

**DOI:** 10.1371/journal.pone.0177928

**Published:** 2017-05-17

**Authors:** Julian Krauskopf, Theo M. de Kok, Shelli J. Schomaker, Mark Gosink, Deborah A. Burt, Patricia Chandler, Roscoe L. Warner, Kent J. Johnson, Florian Caiment, Jos C. Kleinjans, Jiri Aubrecht

**Affiliations:** 1 Department of Toxicogenomics, Maastricht University, Maastricht, the Netherlands; 2 Drug Safety Research and Development, Pfizer Inc., Groton, Connecticut, United States of America; 3 Clinical Research Unit, Pfizer Inc., New Haven, Connecticut, United States of America; 4 Pathology Department, University of Michigan, Ann Arbor, Michigan, United States of America; Saint Louis University, UNITED STATES

## Abstract

MicroRNAs (miRNAs) released into the peripheral circulation upon cellular injury have shown a promise as a new class of tissue-specific biomarkers. We were first to demonstrate that next-generation sequencing analysis of serum from human subjects with acetaminophen-induced liver injury revealed a specific signature of circulating miRNAs. We consequently hypothesized that different types of hepatic liver impairments might feature distinct signatures of circulating miRNAs and that this approach might be useful as minimally invasive diagnostic “liquid biopsies” enabling the interrogation of underlying molecular mechanisms of injury in distant tissues. Therefore we examined serum circulating miRNAs in a total of 72 serum samples from a group of 53 subjects that included patients with accidental acetaminophen overdose, hepatitis B infection, liver cirrhosis and type 2 diabetes as well as gender- and age-matched healthy subjects with no evidence of liver disease. The miRNA signatures were identified using next-generation sequencing that provided analysis for the whole miRNome, including miRNA isoforms. Compared to the healthy subjects, a total of 179 miRNAs showed altered serum levels across the diseased subjects. Although many subjects have elevated alanine aminotransferase suggesting liver impairments, we identified distinct miRNA signatures for different impairments with minimum overlap. Furthermore, the bioinformatics analysis of miRNA signatures revealed relevant molecular pathways associated with the mechanisms of toxicity and or pathogenesis of disease. Interestingly, the high proportion of miRNA isoforms present in the respective signatures indicated a new level of complexity in cellular response to stress or disease. Our study demonstrates for the first time that signatures of circulating miRNAs show specificity for liver injury phenotypes and, once validated, might become useful for diagnosis of organ pathologies as “liquid biopsies”.

## Introduction

MicroRNAs (miRNAs) are regarded as a promising source of tissue-specific biomarkers. These small noncoding RNAs of about 22 nucleotides regulate post-transcriptional gene expression by base pairing with the 3’ untranslated region of the target genes resulting in repressed protein production from the respective gene. MicroRNAs are known to regulate most protein-coding transcripts and therefore are involved in many biological processes [1]. Furthermore, many miRNA species have been identified as tissue-specific [2]. As a result of tissue damage, and/or active secretion, miRNAs are released into the blood circulation [3]. In the bloodstream, miRNAs are stable as they are incorporated within microvesicles or bind to proteins that protect them from RNase digestion [4].

The first demonstration of miRNAs as promising biomarkers of drug-induced liver injury (DILI) was performed in mice treated with toxic doses of acetaminophen (APAP) that resulted in increased serum levels of liver-enriched miRNAs, notably miR-122 and miR-192 [5]. The APAP-induced increases of miR-122 were subsequently confirmed in humans [6]. Since multiple miRNAs can be measured in parallel, several research groups evaluated whether panels of miRNAs might be useful for differentiating various tissue injuries and potentially provide insights into underlying molecular mechanisms. Using multiplexed qPCR analysis, 11 miRNAs were found to differentiate APAP-overdose from ischemic hepatitis [7]. Another multiplexed qPCR analysis found that circulating miRNAs were more sensitive in diagnosing APAP-induced liver injury in humans than alanine amino transference (ALT) [8]. Furthermore, a recent qPCR-based study found alterations of pancreas-enriched miRNAs in the serum of patients with various types of diabetes[9]. Additionally, a microarray-based study reported the upregulation of fibroblast-derived miRNAs in the plasma of patients suffering from chronic heart failure [10]. Lastly, two studies describe a panel of serum miRNAs, possibly released from the brain, as potential biomarkers of Parkinson and Alzheimer’s disease [11, 12].

The multiplexed RT-PCR technology is a closed system dependent on the availability of individual miRNA assays. To cope with this limitation, in our previous study, we used next-generation sequencing (NGS) to interrogate global miRNA signatures in subjects with APAP-overdose [13]. The advantage of NGS technology is its ability to detect the whole miRNome including structural modifications of miRNAs, also called isomiRs. These isomiRs either derive from deviations in the cleavage position by Drosha or Dicer during miRNA maturation in which case the isomiRs are complementary to the sequence of its premature-miRNA. Alternatively, isomiRs may arise from post-transcriptional removal (trimming) or non-templated addition (tailing) of nucleotides by enzymatic reactions [14]. It is assumed that these sequence variations influence miRNA half-life and possibly miRNA target specificity. Interestingly, changes in the relative isomiR distribution have been associated with specific developmental stages and disease progression [15]. Indeed, subjects with APAP-induced liver injury showed a high proportion of isomiRs in their miRNA signatures [13]. Furthermore, a recent study that examined the isomiR signatures in hepatocellular carcinoma tissues revealed that the canonical miRNA was not the most prevalent form in about 39 percent of all miRNAs [16].

In our previous study on subjects with accidental APAP-overdose [13] we identified 36 APAP-inducible miRNAs that included several isomiRs and putative novel miRNAs. Furthermore, the assessment of the biological function of the identified miRNAs was in agreement with molecular mechanisms of APAP-toxicity. This observation prompted us to hypothesize that miRNA signatures in serum might reflect molecular processes in distant tissues and thus serve as “liquid biopsies” by providing insights into mechanisms of toxicity or pathogenesis of disease, specifically to differentiate a variety of liver impairments. Therefore, we have used NGS to study global serum miRNA signatures in groups of healthy subjects (HC), subjects with accidental APAP-overdose, alcohol-induced liver cirrhosis (LC), hepatitis B infection (HBV) and type 2 diabetes mellitus (T2DM) patients with ALT elevation. Furthermore, we evaluated the known molecular functions of the identified miRNAs and their association with the specific hepatopathological processes. We show that the miRNA signatures were indeed able to discriminate between the different impairments. Furthermore, for the first time, we have identified a set of miRNAs with disease-specific distribution of isomiRs and several putatively novel miRNAs of which four are regulated in serum of T2DM patients.

## Methods

### Selection of samples for analysis

Blood samples were collected at the University of Michigan health care system under an approved IRB (2000–05). This study was specifically approved and performed in accordance to the UMHS/Medical School Institutional Review Board (IRBMED) guidelines and regulations, all participants provided informed consent. The selection criteria for healthy subjects included normal levels of ALT, total bilirubin, aspartate aminotransferase, alkaline phosphatase, glucose, blood urea nitrogen, serum creatinine and creatine kinase and the absence of any liver impairment in the medical history. Serum from APAP-overdosed subjects as well as the subjects suffering from HBV, LC and T2DM was collected from leftover diagnostic samples taken during the treatment. The set consisted of 9 APAP patients, 8 LC patients, 7 HBV patients, 7 T2DM patients and 22 HC subjects. The cohort consisted of 21 females and 33 males of an average age of 38 years (characteristics and laboratory values of subjects included in the study see S1 Table).

### MicroRNA isolation

Serum samples were recovered from serum-separator tubes following centrifugation of whole blood at 3000g for 10 minutes at room temperature. Serum samples were kept at 4°C for up to 48 hours before aliquots were frozen at -80°C and stored until shipped for analysis. Serum circulating miRNAs were isolated using the miRNeasy Serum/Plasma Kit (Qiagen). The quality and yield of the miRNA was assessed by means of the Bioanalyzer 2100 using the small RNA Kit (Agilent).

### Small RNA sequencing

RNA samples were prepared for sequencing using the TruSeq Small RNA Preparation Kit (Illumina) and subsequently sequenced using the Illumina HiSeq 2000 sequencing platform (GEO accession GSE90028 and GSE59565). After quality control using FastQC (version 0.11.3) the data were processed using miRDeep2 (v2.0.0.5) [17] and miRBase (release 21) [18] as described earlier [13].

### Statistical analysis

The quantitative miRNA levels retrieved from the miRDeep2 output were analyzed using R (version 3.2.2). To detect differential miRNA levels among the conditions we used the package DESeq2 (version 1.8.1) [19]. Therefore we tested each condition (for the APAP cases only the earliest time-points after overdosing were considered) separately against the HC. The results were filtered for significantly altered miRNAs applying a false discovery rate below 5 percent as described previously [13]. For isomiR quantification we used Isomirage [20] to map all reads to an isomiR database derived from miRBase. The reads were converted to proportions and for each condition the predominant isomiR per miRNA was identified. Based on the remaining sequencing reads miRDeep2 was used to predict novel miRNAs. Briefly, after filtering for other RNA species, the remaining reads were mapped onto the human genome. Subsequently, the results were checked for a valid ratio between precursor and mature read counts and for the possibility of forming a hairpin structure.

### Bioinformatics analysis of identified miRNA signatures for disease specific pathways

Literature-derived pathways were identified from human and animal studies using a modified version of the GenSensor Suite where Ingenuity Pathways replaced KEGG pathways[21]. The GenViewer tool in the GenSensor Suite was used to identify pathways from PubMed which had as MeSH topics: “Liver Cirrhosis”, “Drug-Induced Liver Injury”, “Diabetes Mellitus, Type 2” and “Hepatitis B” (corrected for multiple testing using the qvalue package in R [22]). Pathways were considered significant if a particular disease showed at least a five orders of magnitude higher significance to the other three diseases.

For identification of the molecular pathways from the miRNA signatures, we first assembled lists of all known target genes for each individual miRNA from Firefly Bioworks’s FIREFLY DISCOVERY ENGINE website (http://www.fireflybio.com/). Then the target gene lists for each miRNA signature were analyzed using a modified version of the GenSensor Suite as described above. Finally, liver disease-specific pathways from published literature were compared with pathways derived from miRNA target genes.

## Results

### Small RNA sequencing

The sequencing of a total of 72 serum samples in our study yielded 578 x10^6^ quality-filtered and processed reads across all samples with a mean count of 8 x10^6^ per sample. From these processed reads, 238 x10^6^ (mean count per sample: 3.3 x10^6^) could be aligned to the 2588 precursor miRNAs present in the latest version of the miRNA database miRBase [18]. The remaining reads were most likely derived from other RNA species such as degraded genes, other small RNAs and unknown miRNAs. The count data were filtered for miRNAs that showed at least 20 reads in all samples from one of the five groups of subjects. Based on these criteria we identified 850 mature miRNAs across the 72 samples which were used for further analysis (S2 Table).

During the miRNA isolation from the collected serum samples we observed differences in yield among the groups. The amount of circulating miRNAs isolated from serum of subject with impairments (mean 0.85 ng/μl) was significantly higher (p-value = 0.003218) than the miRNA yield isolated from serum samples of healthy subjects (mean 0.41 ng/μl). Based on the total number of sequencing reads the miRNAs miR-486-5p, miR-92a-3p, miR-22-3p, miR-451a, miR-423-5p, miR-16-5p, miR-142-5p, miR-191-5p, miR-10b-5p and miR-25-3p were the most abundant miRNAs in the serum of HC subjects. Interestingly, these 10 miRNAs accounted for 79%, with miR-486-5p accounting for 40%, of all mapped sequencing reads in control subjects. Apart from miR-22-3p which increased from 7% in the HC to 21% in the APAP samples, the composition of the most abundant miRNAs in APAP, HBV, LC and T2DM was comparable to HC (S3 Table).

### MicroRNA signatures differentiate liver impairments

**Fig 1:**
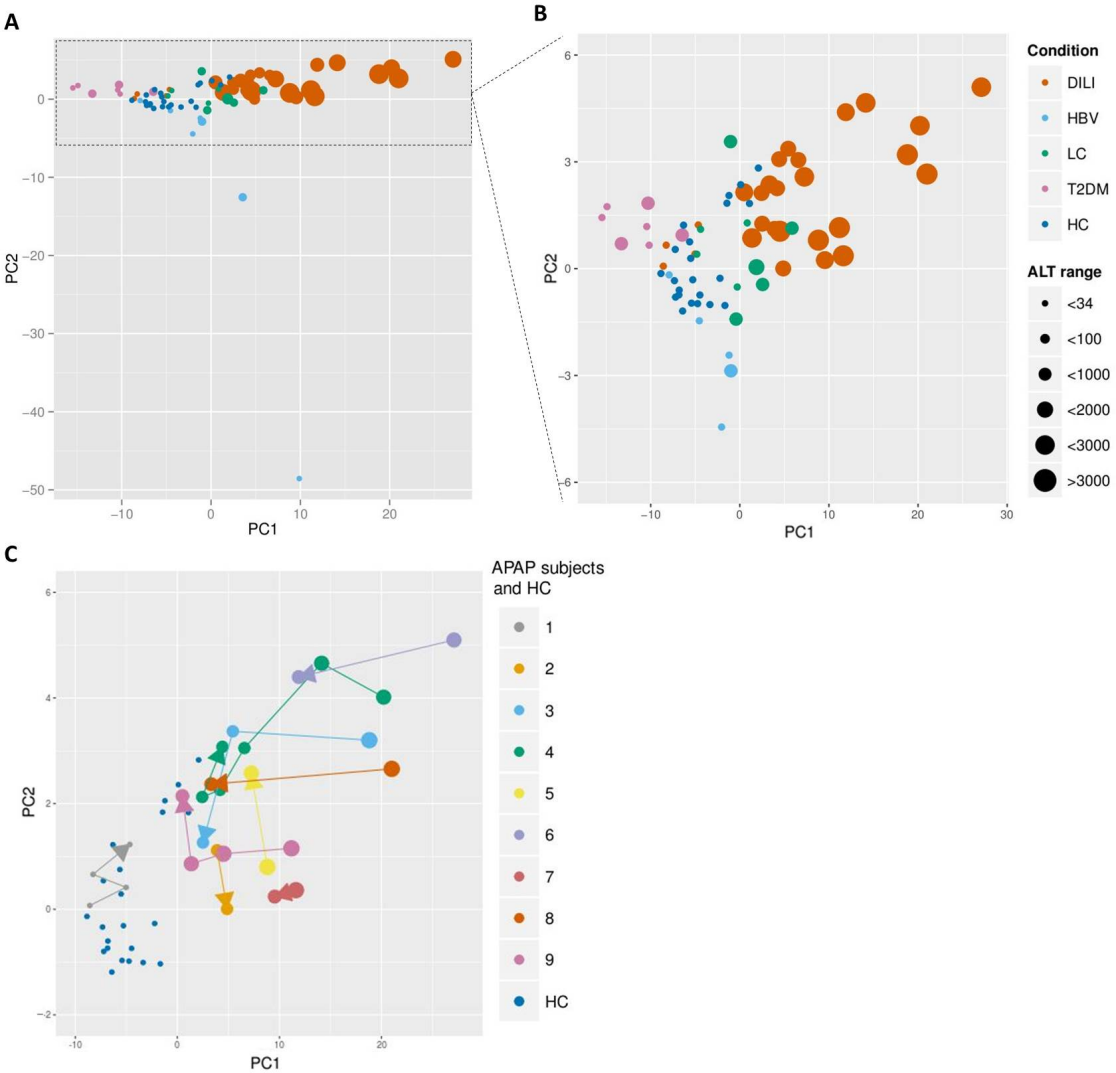
Unsupervised principal component analysis. The PCA presents the serum miRNA and simultaneously the observed ALT levels per subject/sample. The analysis revealed four distinct clusters with subjects/samples clustering according to their respective liver impairment or T2DM (A) (B). Samples, taken at various time points between 1 and 7 days after APAP overdose, show a tendency of clustering towards the control group, which suggests a recovery from liver impairment. Different subjects have varying numbers of time points (ranging 2–6). The temporal sequence of samples is indicated by arrows (C). ALT levels alone were not capable of discriminating patient groups (A) (B) or identifying recovery after APAP overdoses (C).

The (dis)similarities among respective miRNA signatures were evaluated using principal component analysis (PCA). The unsupervised PCA provided four distinct clusters with subjects/samples clustering according to their respective groups (Fig 1A and 1B). Results from the healthy control subjects formed a cluster in the center of the PCA. The fact that samples from patients with liver impairments clustered in various directions from the control group indicates significant differences in miRNA signatures. The PCA analysis showed the largest inter-individual variability in miRNA signatures among HBV subjects (Fig 1A) while T2DM or LC patients were more similar. Interestingly, the ALT levels alone were not capable of identifying recovery after APAP-overdose or of discriminating patient groups according to their liver impairments (Fig 1A and 1B). The APAP sample set consisted of serial samples taken from subjects with APAP-overdose at the admission to the hospital care having at least one follow-up sample for each subject. Close examination of individual subjects in the APAP group showed follow-up samples clustering closer to the control group, thus indicating recovery from APAP-overdose (Fig 1C).

### MicroRNA signatures show specificity to individual liver impairments

**Fig 2:**
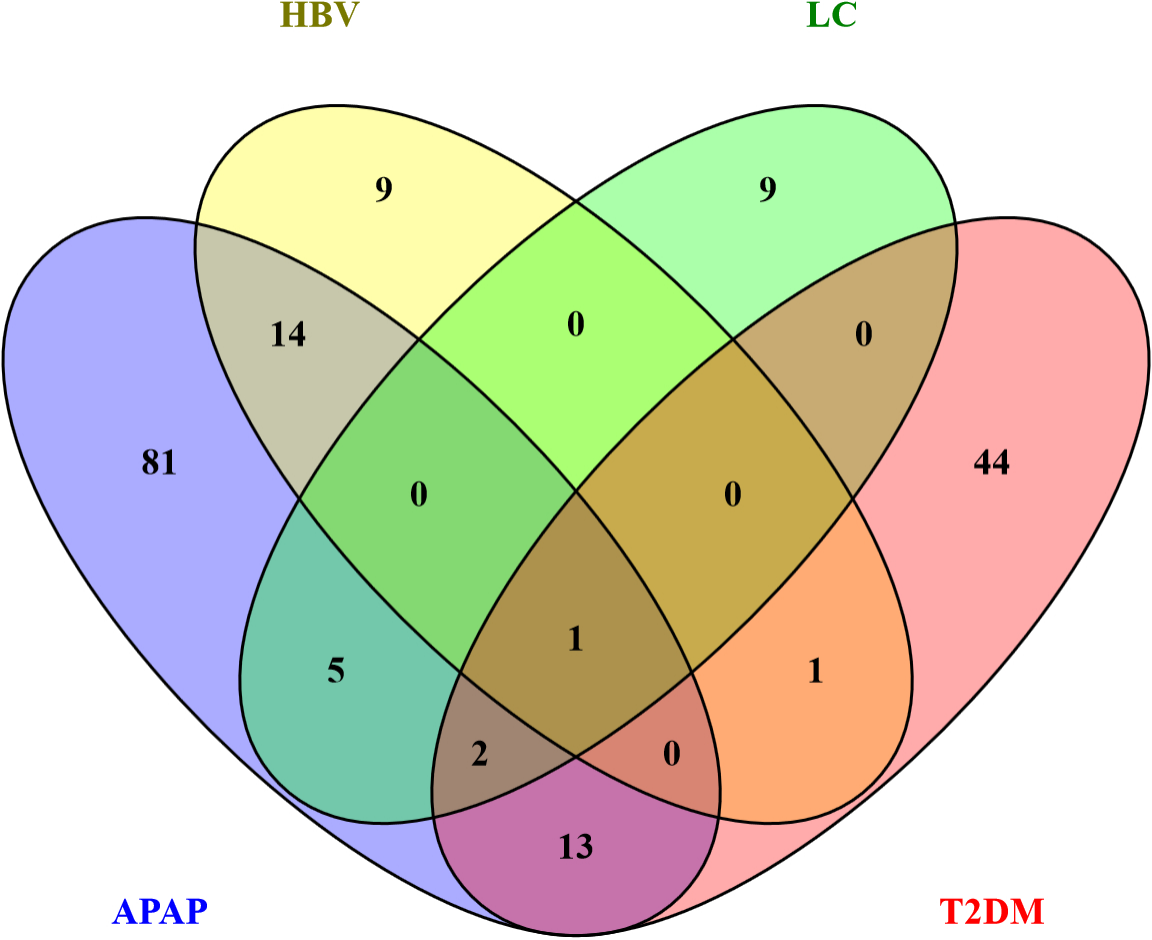
Overlap of individual miRNA signatures. The Venn diagram shows minimal overlap between the liver impairments and T2DM. In total 179 miRNAs were identified (116 in APAP, 25 in HBV, 17 in LC and 61 in T2DM) of which only 1 miRNA was significantly altered among all conditions.

**Fig 3:**
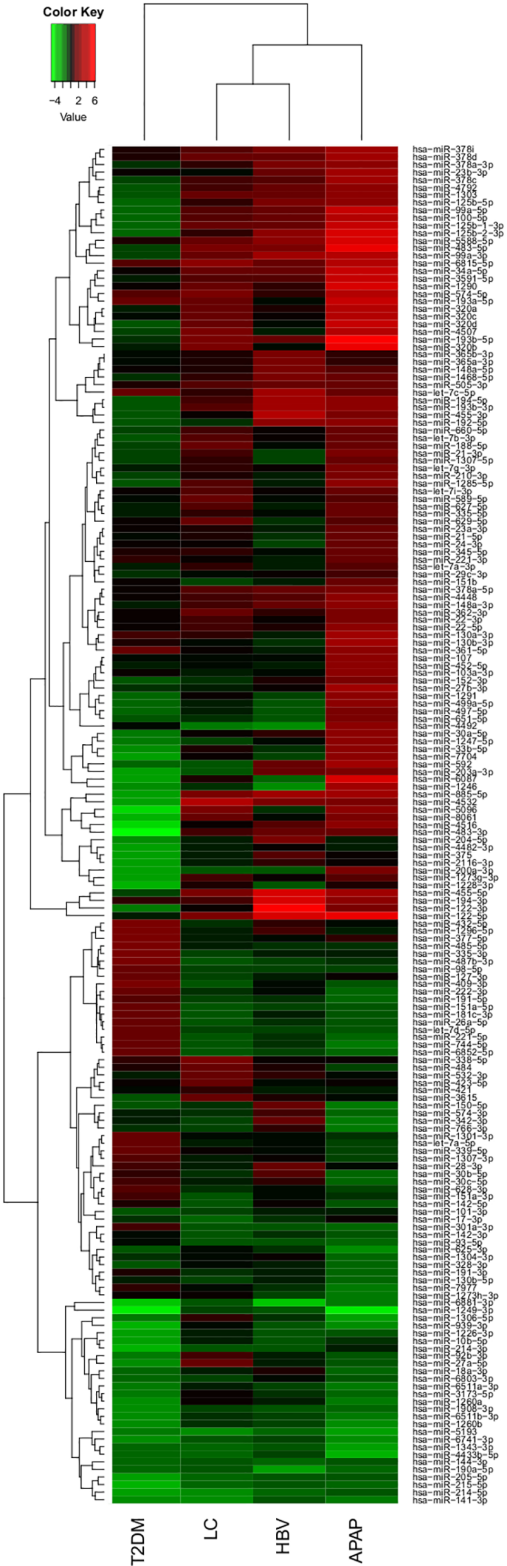
Circulating miRNAs regulated among the different liver impairments and T2DM. The heatmap presents the fold changes of all detected miRNAs across the liver impairments and T2DM compared to HC. The majority of the miRNAs in APAP, HBV and LC were upregulated, while the majority of the miRNAs in T2DM were downregulated. Red indicates increased levels, green indicates decreased levels.

To identify miRNA signatures capable of differentiating respective endpoints of liver injury, we compared the normalized data from subjects suffering from liver impairments with healthy controls. To eliminate the effect of recovery in subjects with APAP-overdose, we have used only the first samples taken at the admission to the hospital for this analysis. The statistical analysis identified a total of 179 miRNAs capable of differentiating liver impairments. The signature for APAP-overdose included 116 miRNAs, the signature for HBV consisted of 25 miRNAs and signatures for LC and T2DM featured 17 and 61 miRNAs, respectively (S4 Table). Interestingly, the distinct miRNA signatures for APAP, HBV, LC and T2DM showed minimal overlap (Fig 2). Close examination of miRNA signatures revealed that the individual miRNAs in signatures for APAP, HBV and LC were, in comparison to controls, mostly increased whereas the majority of the serum miRNAs in subjects with T2DM appeared decreased (Fig 3).

**Fig 4:**
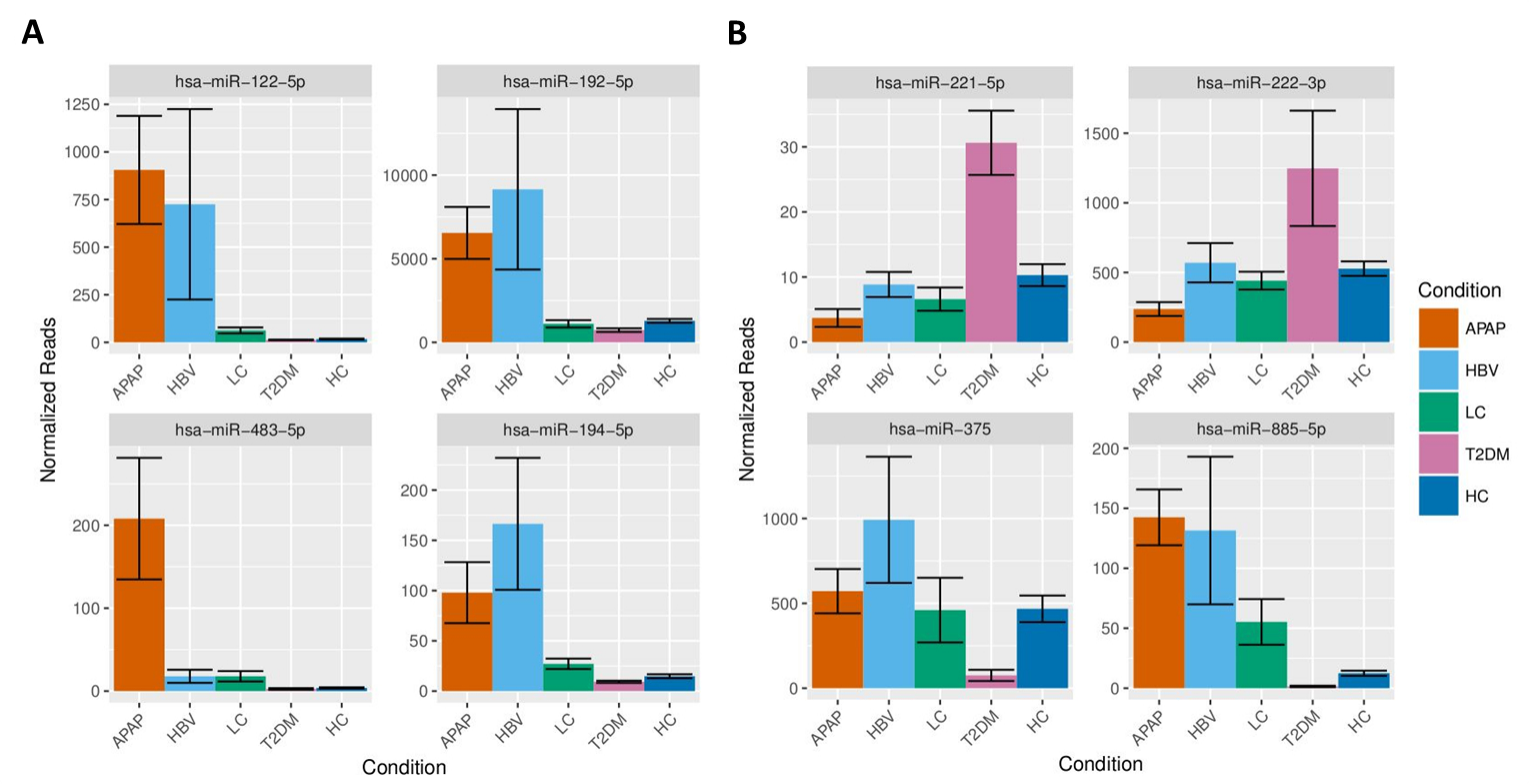
Serum levels of significant regulated miRNAs. The figure presents serum levels, based on normalized sequencing reads, of significantly altered miRNAs in APAP, HBV, LC, T2DM an HC for miR-122-5p, miR-192-5p, miR-483-5p and miR-194-5p (A) and miR-221-5pp, miR-222-3p, miR-375 and miR-885-5p (B).

The APAP miRNA signature consisted of 84 increased and 32 decreased miRNAs. Among these the liver-associated miRNAs miR-122-5p, miR-192-5p, miR-483-5p and miR-194-5p were strongly elevated in the serum of APAP patients (Fig 4A). Within the signature of 116 miRNAs, the regulation of 32 out of 33 miRNAs reported in an earlier study was confirmed [13]. The HBV signature consisted of a total 25 miRNAs of which 23 miRNAs had increased and 2 decreased serum levels. Interestingly, 9 miRNAs were unique for HBV patients and 15 were shared with the miRNAs signature from subjects overdosed on APAP and from LC patients (Fig 2). A significant elevation of both 5p and 3p miRNAs was observed for miR-99a, miR-122, miR-194, miR-125b and miR-455 in HBV patients compared to HC. The miRNA signature for LC showed a total of 17 miRNAs of which 14 had increased and 3 decreased serum levels. Nine miRNAs of the LC signature were unique to LC and 8 miRNAs were shared with the APAP, HBV and T2DM signatures (Fig 2). The T2DM signature featured 61 miRNAs of which 22 were increased and 39 decreased levels. Of these miRNAs, 44 were unique to T2DM and 16 were shared with the other signatures. Remarkably, 10 of the 16 shared miRNAs were inversely regulated in T2DM patients in comparison to subjects having overdosed on APAP (such as miR-885 -5p, Fig 4B). The level of the diabetes-related miR-375 was found to be significantly decreased in T2DM patients while some T2DM-associated miRNAs were upregulated such as miR-221 and miR-222 (Fig 4B).

### MicroRNA signatures correspond to molecular mechanisms related to liver injury subtypes

To evaluate the potential of miRNA signatures to provide insights into molecular mechanisms of toxicity and pathogenesis of disease, we first identified the most relevant pathways for the respective impairments from published literature and then compared them with pathways derived from bioinformatics analysis of miRNA target genes. Since all four disease states are known to alter many of the same metabolic processes, it is not surprising that an analysis of the genes known to be associated with the respective disease phenotypes identifies many similar pathways (S1 File). Therefore, to select distinct pathways for each disease state, we arbitrarily selected pathways with at least five orders of magnitude higher significance for a particular disease. As expected, the disease state-specific pathways derived from literature were in accordance with the known pathogenesis of these diseases. In all cases, these disease state specific pathways were found to be also significantly enriched among pathways derived from analysis of miRNA target genes (Table 1). For instance, the miRNA signature for APAP-overdosing featured oxidative stress and apoptosis signaling, whereas the LC signature referred to immune-related interferon signaling and the antigen presentation pathway. The LC miRNA signature suggests tissue remodeling and inflammation by including the epithelial-mesenchymal transition pathway, adhesion and diapedesis elements. Furthermore, the T2DM miRNA signature was in full agreement with the molecular pathology of the disease since it consisted of T2DM-related pathways such as AMPK, insulin and generic diabetes signaling pathways.

**Table 1:** Comparison of pathways derived from literature analysis with pathways derived from miRNA target genes.

**Cirrhosis Pathways**	**Cirrhosis pathways from PubMed**	**Cirrhosis pathways associated with identified miRNAs**
Bladder Cancer Signaling	3.29E-14	8.25E-16
Regulation of the Epithelial-Mesenchymal Transition Pathway	7.06E-15	8.25E-16
Agranulocyte Adhesion and Diapedesis	5.55E-15	8.25E-16
Granulocyte Adhesion and Diapedesis	5.55E-15	8.25E-16
Glioblastoma Multiforme Signaling	1.47E-11	8.25E-16
STAT3 Pathway	2.40E-12	8.25E-16
Antiproliferative Role of TOB in T Cell Signaling	3.16E-08	8.51E-15
**DILI Pathways**	**DILI pathways from PubMed**	**DILI pathways associated with identified miRNAs**
NRF2-mediated Oxidative Stress Response	9.02E-15	5.75E-16
Apoptosis Signaling	8.33E-13	5.75E-16
Acetone Degradation I (to Methylglyoxal)	6.13E-09	1.20E-08
Bupropion Degradation	6.13E-09	2.38E-08
Estrogen Biosynthesis	6.74E-08	8.81E-08
**Hepatitis B Pathways**	**HBV pathways from PubMed**	**HBV pathways associated with identified miRNAs**
Antigen Presentation Pathway	8.02E-15	8.30E-06
Interferon Signaling	8.02E-15	7.59E-10
**Type-2 Diabetes Pathways**	**T2D pathways from PubMed**	**T2D pathways associated with identified miRNAs**
AMPK Signaling	2.54E-13	5.96E-16
Insulin Receptor Signaling	1.88E-13	5.96E-16
TR/RXR Activation	7.76E-11	5.96E-16
eNOS Signaling	1.23E-09	5.96E-16
Type II Diabetes Mellitus Signaling	1.20E-14	5.96E-16
Leptin Signaling in Obesity	1.20E-09	5.96E-16
Role of NFAT in Cardiac Hypertrophy	1.19E-07	5.96E-16

Significance values for pathways predicted to be the most specific for each individual disease state were calculated based on published literature or known miRNA target genes. Pathways in the table are arranged by relative specificity determined from the literature for individual disease state.

### Circulating isomiRs discriminate between liver impairments

Isoforms of miRNAs, called isomiRs, are thought to be involved in the fine tuning of the miRNA-mediated cell signaling. Therefore, we used NGS to assess the isomiR composition of individual circulating miRNAs. Our data show that in all groups of subjects (APAP, HBV, LC, T2DM and HC) about 48 percent of the total sequencing reads represented the canonical miRNA. The second most abundant miRNA isoform was the 3’ trimmed isomiR with 25–42% distribution across groups of subjects. Although levels of 3’ trimmed isomiRs in HBV and T2DM subjects were comparable with HC (25–28%), the levels of 3’ trimmed isomiRs in APAP-Overdosed subjects were significantly elevated to 42% (p-value = 0.003405). For LC subjects we observed a similar trend of increased levels of 3’ trimmed isomiRs. Furthermore, isomiRs with 3’ additions seemed to be more prevalent in HBV, T2MD and HC than in APAP-overdosed subjects and LC patients (p-value = 0.03629) (Table 2).

**Table 2:** IsomiR proportions among the liver impairments and T2DM.

	APAP (%)	HBV (%)	LC (%)	T2DM (%)	HC (%)
**Canonical**	48	48	49	48	49
**Trimmed**	42	28	36	25	28
**3' A addition**	2	6	3	4	5
**3' addition nt**	4	9	6	9	8
**3' addition templ.**	3	4	3	7	5
**3' U addition**	1	2	1	3	2
**Other forms**	1	2	1	3	2

Analysis of isomiRs revealed different proportions among the trimmed isoform in APAP compared to HC, HBV and T2DM while the proportion of the canonical form was stable among all conditions.

**Fig 5:**
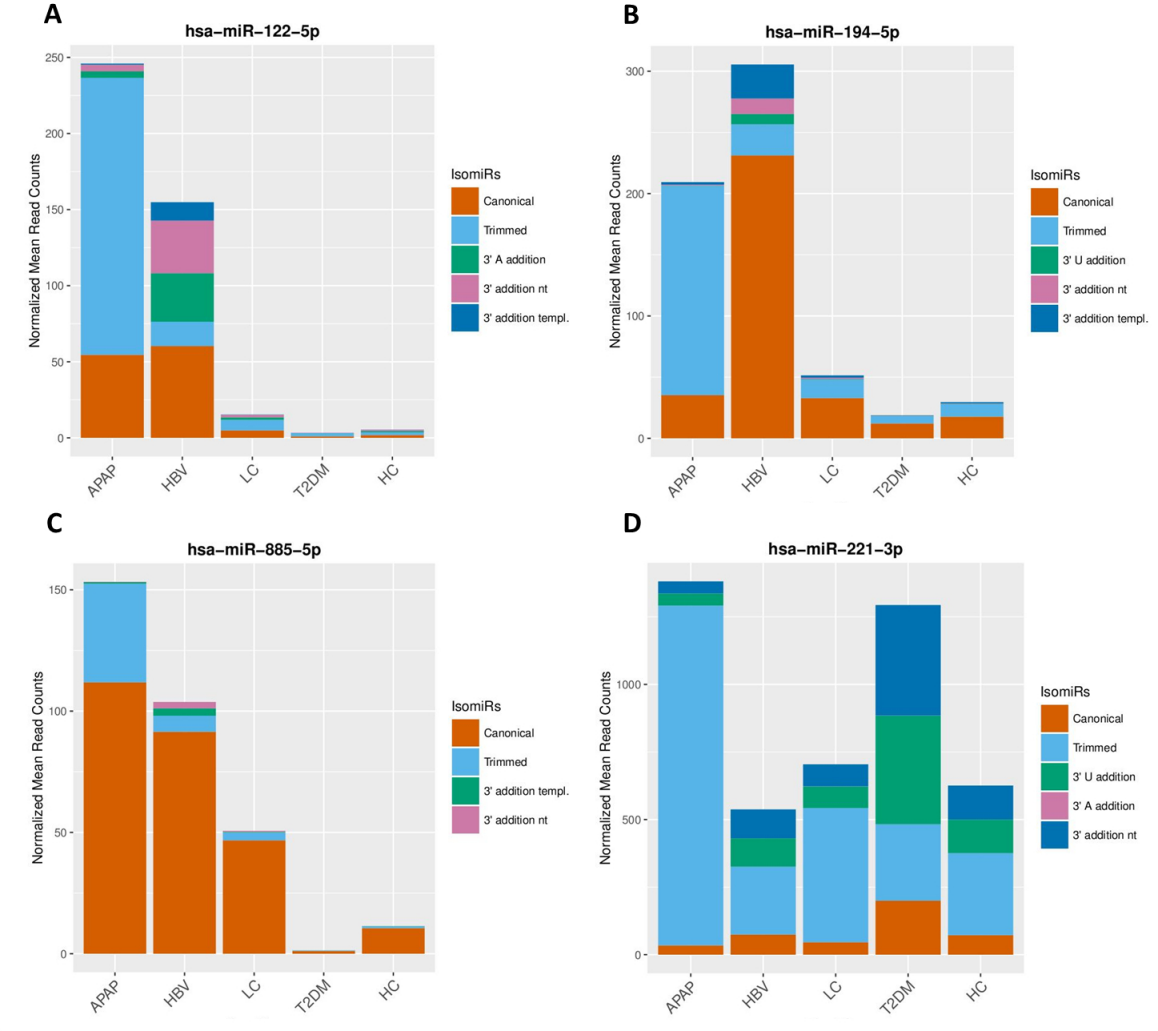
Different isomiR spectrums in APAP, HBV and T2DM. The figure presents the isomiR composition, based on normalized mean sequencing reads, in APAP, HBV, LC, T2DM an HC for miR-122-5p (A), miR-194-5p (B), miR-885-5p (C) and miR-221-3p (D).

The spectrum of isomiRs showed a striking difference across individual miRNAs and impairments (Fig 5). For instance, the canonical form of miR-122-5p was increased to the similar extent in APAP-overdosed subjects and HBV patients. However, the isomiR spectrum of miR-122-5p showed significant differences. In the APAP cases, the miR-122-5p was predominantly present as a trimmed isomiR while, in the HBV samples, the isomiR spectrum consisted of mainly the canonical form, an isomiR with an enzymatic addition of several adenine (A/AA) and a non-templated extension of adenine and uracil (AU/AAU) (Fig 5A). A similar isomiR distribution is presented for miR-194-5p and miR-885-5p in Fig 5B and 5C. Furthermore, we observed distinguished circulating isomiR proportions in T2DM patients for miR-221, showing an underrepresentation of the trimmed isomiRs and an overrepresentation of isomiRs with 3’ additions of uracil (U/UU) and an extension of uracil and adenine (UA/UAU) (5D). By defining the most abundant isomiRs for all miRNAs per condition, we identified a set of 58 miRNAs showing aberrant isomiR proportions that could provide support to discriminate among the different conditions (S5 Table).

### Novel predicted putative miRNAs

In total, 47 miRNA-like small RNAs passed our criteria (see Methods) and were analyzed jointly with the miRBase sequences for differential regulation (see S6 Table). We identified one putative miRNA to be upregulated in both APAP-overdosed and LC subjects, one predicted miRNA to be upregulated in LC cases and downregulated in T2DM subjects, and 4 other predicted miRNAs to be solely regulated in T2DM patients (see S7 Table).

## Discussion

In this study, we have used NGS to evaluate the global signatures of circulating miRNAs in the serum of subjects with APAP-induced liver injury, LC, HBV, T2DM and gender- and age-matched healthy controls. We identified distinct miRNA signatures consisting of biologically relevant miRNAs that were capable of differentiating groups of subjects with respect to the underlying cause of liver injury. We are also the first to show that a distribution of isomiRs for 58 miRNAs provides further differentiation among groups of subjects with different impairments. Furthermore, we identified 47 putative novel miRNAs of which 4 were significantly regulated in the serum of T2DM patients.

In all five groups, we observed miR-486-5p to be the most abundant miRNA, followed by miR-92a-3p in HBV, LC and HC, and miR-22-3p in T2DM and upon APAP-overdosing. Nine out of the 10 most abundantly expressed miRNAs have been described in an earlier study to be among the most abundant miRNAs circulating in healthy individuals (only miR-142-5p was not reported) [23]. Seven out of these 10 miRNAs (miR-486-5p, miR-92a-3p, miR-451a, miR-16-5p, miR-142-5p, miR-191-5p and miR-25-3p) have been found to be highly expressed in various blood cell types [24]. By contrast, miR-22-3p, miR-423-5p and miR-10b-5p have not been found in red blood cells. The presence of these miRNAs in the peripheral circulation has not yet been reported, however, miR-22-3p is also abundantly expressed in the liver [25]. Interestingly, miR-22 was significantly upregulated when comparing APAP-overdosed patients with HC. Apart from this hepatic miRNA, the majority of the highly abundant miRNAs were not altered among the five groups. While we observed a significant difference in RNA yield between healthy and diseased subjects, the disease-specific altered miRNA levels were observed mainly in the low abundantly present miRNAs. For instance the liver-enriched miRNAs miR-122-5p and miR-192-5p increased from < 0.01% and 0.27% in HC to 0.15% and 1.08% in the APAP samples (S3 Table). Consequently, the increased RNA yield is to some extend due to other small RNA species that are released into the circulation as a consequence of liver impairment.

We have identified a total of 179 miRNAs with altered serum levels that differentiated subjects with liver impairments and T2DM from healthy subjects. The miRNA analysis provided distinct miRNA signatures for each group of subjects with minimum overlap (Fig 2). In fact, only one miRNA (miR-885-5p) was in common for all groups; however, it was increased in APAP, HBV and LC, and decreased in T2DM patients. This suggests high specificity of miRNA signatures in human serum. This is in accordance with 2 studies showing differentiation between APAP-induced injury and hepatitis [8], and between Alcoholic, Drug-induced and inflammatory liver disease [26].

The miRNA signature of APAP-induced liver injury consisted of 116 regulated miRNAs (S4 Table). The APAP signature comprised 32 out of 33 miRNAs reported in our earlier publication [13] that included fewer subjects than the current study. The fact that we were able to confirm the APAP signature from our previous study indicates an excellent reproducibility of miRNA signatures and NGS technology across studies. On the other hand, a recently published evaluation of miRNA signatures of APAP-overdosed children using NGS reported only 8 miRNAs [27] from which only 2 miRNAs (miR-122-5p, miR-125b-5p) were also identified in our studies. This discrepancy might be caused by differences in miRNA signatures between children and adults or by differences in study design and methodology of the miRNA analysis by NGS such as differences in sequencing depth. Recently, a qPCR-based study examined circulating miRNAs in patients with acute liver injury and found 281 miRNAs to discriminate between patients with and without APAP-toxicity. The study found a ratio of upregulated miR-122 and downregulated miR-483-3p to reliably predict hepatotoxicity. However, in our study, the serum levels of miR-483-3p were not affected by APAP-induced liver injury, while miR-483-3p was significantly decreased in the serum of subjects with T2DM. Nevertheless, the signature presented here confirmed the upregulation of 28 and downregulation of 8 miRNAs [8]. Differences in the results are likely due to distinct quantification techniques, statistics, but also to different study designs.

The serum miRNA signature found in HBV patients consisted of 25 miRNAs. Interestingly, the signature revealed a simultaneous increase of the 5p and 3p forms for miR-122, miR-125b, miR-194, miR-455 and miR-99a, which has been previously observed in the case of miR-455 in HBV-infected children [28]. Furthermore, the elevated levels of miR-122, miR-194 and miR-125b could be related to immunity and inhibition of HBV [29–31]. Next to the miRNAs elevated in all subtypes of liver injury, 18 miRNAs were uniquely regulated in the HBV or LC subjects. These miRNAs support the discrimination between the subtypes of liver disease.

The serum miRNA signature in T2DM patients was distinct from serum miRNA signatures in subjects with liver impairments. T2DM patients exhibited a general decrease of circulating miRNAs in contrast to APAP-overdosing, as well as to HBV and LC patients (Fig 3). This could be an effect of decreased miRNA expression in the affected organs or decreased leakage or secretion of miRNAs from tissues. The T2DM signature consisted of 61 miRNAs, of which 44 miRNAs were unique to T2DM and 10 miRNAs were inversely correlated when comparing T2DM patients and APAP-overdosed subjects (S4 Table). Interestingly, miR-375 that is specifically expressed in islet cells of the pancreas and regulates insulin expression [32] was found to be significantly decreased exclusively in serum of T2DM subjects. Since the decrease of miR-375 expression in beta islet cells has been shown to cause an increase of insulin production, the decrease of circulating levels of serum miR-375 observed in our study might reflect the high insulin production that is a hallmark of T2DM. Although this is a compelling hypothesis, it needs to be further evaluated particularly in light of a published study that is in conflict with our results [9]. The discrepant results might be caused by differences in patient selection, analytical methodologies and study design. Despite the predominant decrease of miRNA levels in T2DM patients, serum levels of several miRNAs such as miR-221 and miR-222 were increased. Interestingly, both miRNAs have been reported to promote intimal thickening in internal mammary artery segments from T2DM patients [33].

Since some of the miRNAs in the signatures exhibit specificity for individual impairments, we set out to assess whether a pathway analysis could provide mechanistic insights. The identified pathways corresponded to known biology for respective disease states. For example, AMPK and insulin signaling have well established central roles in T2DM [34, 35]. Similarly, antigen presentation and interferon signaling play major roles in hepatitis B [36, 37]. The miRNA signature for APAP-overdosing featured oxidative stress and apoptosis signaling [38, 39]. Finally, the LC miRNA signature suggests tissue remodeling and inflammation by including the epithelial-mesenchymal transition pathway, adhesion and diapedesis elements [40, 41]. Although promising, our pathway analysis did not consider 3p/5p forms and influence of structural modification of miRNAs (isomiRs) on miRNA regulation of gene expression. Nevertheless, our bioinformatics analysis indicates the potential of using miRNA signatures to gain insights into disease mechanisms.

The mechanisms underlying the increased levels of circulating miRNA upon tissue damage or disease are not yet clear. In the case of acute organ damage that is associated with massive cell death, miRNAs are believed to leak from damaged cells into the circulation. However, recently published studies highlight the importance of active miRNA secretion in relation to cell-to-cell communication [42]. These circulating extracellular miRNAs are present in different forms such as embedded in exosomes or bound to proteins [43]. Since the whole serum includes both the exosome- and protein-bound fraction of miRNAs, we have used this for our study. Furthermore, since several miRNAs such as miR-122 or mir-375 show excellent tissue specificity, miRNA platforms show promise as potential tissue specific biomarkers.

The miRNA signatures included high proportions of isomiRs (Table 2). In fact, 58 miRNAs in our study showed a distinct distribution or spectrum of isomiRs across groups of subjects (S5 Table). IsomiRs are formed either by an alternative dicing event (templated form), post-transcriptional removal (trimming) or non-templated addition (tailing) of nucleotides of canonical miRNAs. IsomiRs were shown to exert different functionality [15] than their canonical counterparts that results in fine-tuning of the miRNA-mediated cell signaling. In our study, we observed an overrepresentation of trimmed isomiRs in patients with acute liver injury such as in subjects with the APAP-overdose (Table 2). In contrast, the proportions of trimmed isomiRs in the miRNA signatures of subjects with chronic impairments such as T2DM or HBV were similar to healthy controls (Table 2). The increased proportion of trimmed isomiRs suggests increased exonucleolytic cleavage activity in the blood of APAP-overdosed patients or alternative dicing during miRNA biogenesis. An effect on miRNA activity or miRNA half-life is not yet known for trimmed isoforms. However, we observed an overrepresentation of an adenylated miR-122 in HBV patients. This enzymatic addition of adenosine has been described as selective stabilization of miR-122 in liver [44]. As miR-122 plays an important role in inhibition of HBV reproduction in human, selective stabilization of this miRNAs might be supportive to the immune defense [29]. Even though miR-122 is enriched in both APAP-overdosed subjects and HBV patients, our results suggest the potential to distinguish the two subtypes based on miR-122 isomiR proportions alone (Fig 5). Furthermore, we observed increased uridylation of miR-221-3p and miR-99-5p in T2DM patients. The biological effect of this nucleotide addition is not yet clear, however, the uridylation of pre-miRNA let-7 was shown to inhibit Dicer and promote let-7 decay [45]. Although more work needs to be done to understand the role of isomiRs in cellular response to toxic effects or disease, our work is the first to indicate the potential of isomiRs in these processes.

Furthermore, among the 47 predicted putative miRNAs that were identified in our study, 6 putative miRNAs had significantly altered serum levels in subjects with liver impairments or T2DM. For instance, the serum level of the seq_5 was increased in LC subjects while being decreased in T2DM subjects. Another sequence, seq_22, was found to have increased serum levels in both APAP-overdosed and LC subjects (S7 Table). Since we could not assign any functional annotation to these novel sequences from literature, more research needs to be done to understand their role in cellular responses to toxic effect and diseases. The fact that we identified new putative miRNA in our study demonstrates the advantages of NGS as an open system for miRNA analysis.

## Conclusions

In conclusion, we have identified signatures of circulating miRNAs that were specific for various phenotypes of liver injury and T2DM. Additionally, we report for the first time a set of isomiRs that complement the hepatic disease-specific spectrum of canonical miRNAs. Although further studies need to validate the respective miRNA signatures in a wider range of patients and conditions, our findings indicate the potential of miRNA signatures to be used as “liquid biopsies”, thereby also providing mechanistic information relevant for cellular injury in distant tissues. Our work generates a foundation for potentially developing a non-invasive diagnostic screening test for liver pathology, capable of providing clinically relevant information regarding toxic effects of chemicals and the pathogenesis of diseases in the liver.

## Supporting information

S1 Table Patient characteristics and clinical chemistry data. The table lists all subject including metadata and clinical chemistry paramenters.
(DOCX)

S2 Table List of miRNAs detected across all subjects. Complete circulating miRNAs detected in the serum of all subjects.
(DOCX)

S3 Table Percentages of mapped sequencing reads across all liver impairments and T2DM. List of all miRNAs and its presence in serum in percent.
(DOCX)

S4 Table Individual miRNA signatures. List of significantly altered miRNAs detected for all liver impairments and T2DM.
(DOCX)

S5 Table List of the most abundant miRNA isoforms detected. The list consist of miRNAs that show differences in the predominantly observed miRNA the conditions.
(DOCX)

S6 Table List of small RNA like new predicted miRNAs. List of small RNA like sequences that passed our criteria to be potential novel miRNAs.
(DOCX)

S7 Table Significantly altered small RNA like miRNAs. List of small RNA like sequences that passed our criteria to be potential novel miRNAs and are altered upon liver disease and T2DM.
(DOCX)

S1 File Identified pathways with individual signatures. The table shows all pathways identified with the individual miRNA signatures.
(XLSX)

## References

[1] Bartel DP. MicroRNAs: target recognition and regulatory functions. Cell. 2009;136: 215–33. doi: 10.1016/j.cell.2009.01.002 19167326 10.1016/j.cell.2009.01.002 PMC3794896

[2] Liang Y, Ridzon D, Wong L, Chen C. Characterization of microRNA expression profiles in normal human tissues. BMC Genomics. 2007;8: 166 doi: 10.1186/1471-2164-8-166 17565689 10.1186/1471-2164-8-166 PMC1904203

[3] Creemers EE, Tijsen AJ, Pinto YM. Circulating microRNAs: novel biomarkers and extracellular communicators in cardiovascular disease? Circ Res. 2012;110: 483–95. doi: 10.1161/CIRCRESAHA.111.247452 22302755 10.1161/CIRCRESAHA.111.247452

[4] Cortez MA, Bueso-Ramos C, Ferdin J, Lopez-Berestein G, Sood AK, Calin GA. MicroRNAs in body fluids—the mix of hormones and biomarkers. Nature reviews Clinical oncology. 2011;8: 467–77. doi: 10.1038/nrclinonc.2011.76 21647195 10.1038/nrclinonc.2011.76 PMC3423224

[5] Wang K, Zhang S, Marzolf B, Troisch P, Brightman A, Hu Z, et al Circulating microRNAs, potential biomarkers for drug-induced liver injury. Proceedings of the National Academy of Sciences of the United States of America. 2009;106: 4402–7. doi: 10.1073/pnas.0813371106 19246379 10.1073/pnas.0813371106 PMC2657429

[6] Starkey Lewis PJ, Dear J, Platt V, Simpson KJ, Craig DG, Antoine DJ, et al Circulating microRNAs as potential markers of human drug-induced liver injury. Hepatology. 2011;54: 1767–76. doi: 10.1002/hep.24538 22045675 10.1002/hep.24538

[7] Ward J, Kanchagar C, Veksler-Lublinsky I, Lee RC, McGill MR, Jaeschke H, et al Circulating microRNA profiles in human patients with acetaminophen hepatotoxicity or ischemic hepatitis. Proceedings of the National Academy of Sciences of the United States of America. 2014;111: 12169–74. doi: 10.1073/pnas.1412608111 25092309 10.1073/pnas.1412608111 PMC4143020

[8] Vliegenthart AD, Shaffer JM, Clarke JI, Peeters LE, Caporali A, Bateman DN, et al Comprehensive microRNA profiling in acetaminophen toxicity identifies novel circulating biomarkers for human liver and kidney injury. Scientific reports. 2015;5: 15501 doi: 10.1038/srep15501 26489516 10.1038/srep15501 PMC4614545

[9] Seyhan AA, Nunez Lopez YO, Xie H, Yi F, Mathews C, Pasarica M, et al Pancreas-enriched miRNAs are altered in the circulation of subjects with diabetes: a pilot cross-sectional study. Scientific reports. 2016;6: 31479 doi: 10.1038/srep31479 27558530 10.1038/srep31479 PMC4997329

[10] Li H, Fan J, Yin Z, Wang F, Chen C, Wang DW. Identification of cardiac-related circulating microRNA profile in human chronic heart failure. Oncotarget. 2016;7: 33–45. doi: 10.18632/oncotarget.6631 26683101 10.18632/oncotarget.6631 PMC4807981

[11] Ding HX, Huang Z, Chen MJ, Wang C, Chen X, Chen JN, et al Identification of a panel of five serum miRNAs as a biomarker for Parkinson's disease. Parkinsonism Relat D. 2016;22: 68–73. 10.1016/j.parkreldis.2015.11.014 26631952

[12] Galimberti D, Villa C, Fenoglio C, Serpente M, Ghezzi L, Cioffi SMG, et al Circulating miRNAs as Potential Biomarkers in Alzheimer's Disease. J Alzheimers Dis. 2014;42: 1261–7. doi: 10.3233/JAD-140756 25024331 10.3233/JAD-140756

[13] Krauskopf J, Caiment F, Claessen SM, Johnson KJ, Warner RL, Schomaker SJ, et al Application of high-throughput sequencing to circulating microRNAs reveals novel biomarkers for drug-induced liver injury. Toxicological sciences: an official journal of the Society of Toxicology. 2015;143: 268–76. 25359176 10.1093/toxsci/kfu232

[14] Ameres SL, Zamore PD. Diversifying microRNA sequence and function. Nature reviews Molecular cell biology. 2013;14: 475–88. doi: 10.1038/nrm3611 23800994 10.1038/nrm3611

[15] Williams Z, Ben-Dov IZ, Elias R, Mihailovic A, Brown M, Rosenwaks Z, et al Comprehensive profiling of circulating microRNA via small RNA sequencing of cDNA libraries reveals biomarker potential and limitations. Proceedings of the National Academy of Sciences of the United States of America. 2013;110: 4255–60. doi: 10.1073/pnas.1214046110 23440203 10.1073/pnas.1214046110 PMC3600502

[16] Wojcicka A, Swierniak M, Kornasiewicz O, Gierlikowski W, Maciag M, Kolanowska M, et al Next generation sequencing reveals microRNA isoforms in liver cirrhosis and hepatocellular carcinoma. Int J Biochem Cell Biol. 2014;53: 208–17. doi: 10.1016/j.biocel.2014.05.020 24875649 10.1016/j.biocel.2014.05.020

[17] Friedlander MR, Mackowiak SD, Li N, Chen W, Rajewsky N. miRDeep2 accurately identifies known and hundreds of novel microRNA genes in seven animal clades. Nucleic Acids Res. 2012;40: 37–52. doi: 10.1093/nar/gkr688 21911355 10.1093/nar/gkr688 PMC3245920

[18] Griffiths-Jones S. miRBase: the microRNA sequence database. Methods Mol Biol. 2006;342: 129–38. doi: 10.1385/1-59745-123-1:129 16957372 10.1385/1-59745-123-1:129

[19] Love MI, Huber W, Anders S. Moderated estimation of fold change and dispersion for RNA-seq data with DESeq2. Genome Biol. 2014;15: 550 doi: 10.1186/s13059-014-0550-8 25516281 10.1186/s13059-014-0550-8 PMC4302049

[20] Muller H, Marzi MJ, Nicassio F. IsomiRage: From Functional Classification to Differential Expression of miRNA Isoforms. Frontiers in bioengineering and biotechnology. 2014;2: 38 doi: 10.3389/fbioe.2014.00038 25325056 10.3389/fbioe.2014.00038 PMC4179619

[21] Gosink M, Khuri S, Valdes C, Jiang Z, Tsinoremas NF. GenSensor Suite: A Web-Based Tool for the Analysis of Gene and Protein Interactions, Pathways, and Regulation. Adv Bioinformatics. 2011;2011: 271563 doi: 10.1155/2011/271563 22194743 10.1155/2011/271563 PMC3238354

[22] Dabney A, Storey JD, Warnes G. qvalue: Q-value estimation for false discovery rate control. R package version. 2010;1:

[23] Tonge DP, Gant TW. What is normal? Next generation sequencing-driven analysis of the human circulating miRNAOme. Bmc Mol Biol. 2016;17:10.1186/s12867-016-0057-9 PMC4748454 26860190

[24] Pritchard CC, Kroh E, Wood B, Arroyo JD, Dougherty KJ, Miyaji MM, et al Blood cell origin of circulating microRNAs: a cautionary note for cancer biomarker studies. Cancer Prev Res (Phila). 2012;5: 492–7. 22158052 10.1158/1940-6207.CAPR-11-0370 PMC4186243

[25] Kaur K, Vig S, Srivastava R, Mishra A, Singh VP, Srivastava AK, et al Elevated Hepatic miR-22-3p Expression Impairs Gluconeogenesis by Silencing the Wnt-Responsive Transcription Factor Tcf7. Diabetes. 2015;64: 3659–69. doi: 10.2337/db14-1924 26193896 10.2337/db14-1924

[26] Bala S, Petrasek J, Mundkur S, Catalano D, Levin I, Ward J, et al Circulating microRNAs in exosomes indicate hepatocyte injury and inflammation in alcoholic, drug-induced, and inflammatory liver diseases. Hepatology. 2012;56: 1946–57. doi: 10.1002/hep.25873 22684891 10.1002/hep.25873 PMC3486954

[27] Yang X, Salminen WF, Shi Q, Greenhaw J, Gill PS, Bhattacharyya S, et al Potential of extracellular microRNAs as biomarkers of acetaminophen toxicity in children. Toxicol Appl Pharmacol. 2015;284: 180–7. doi: 10.1016/j.taap.2015.02.013 25708609 10.1016/j.taap.2015.02.013 PMC4558622

[28] Winther TN, Jacobsen KS, Mirza AH, Heiberg IL, Bang-Berthelsen CH, Pociot F, et al Circulating MicroRNAs in Plasma of Hepatitis B e Antigen Positive Children Reveal Liver-Specific Target Genes. Int J Hepatol. 2014;2014: 791045 doi: 10.1155/2014/791045 25580300 10.1155/2014/791045 PMC4281389

[29] Chen Y, Shen A, Rider PJ, Yu Y, Wu K, Mu Y, et al A liver-specific microRNA binds to a highly conserved RNA sequence of hepatitis B virus and negatively regulates viral gene expression and replication. FASEB journal: official publication of the Federation of American Societies for Experimental Biology. 2011;25: 4511–21. 21903935 10.1096/fj.11-187781 PMC3236624

[30] Jiang X, Kanda T, Wu S, Nakamura M, Miyamura T, Nakamoto S, et al Regulation of microRNA by hepatitis B virus infection and their possible association with control of innate immunity. World J Gastroentero. 2014;20: 7197–206. 10.3748/wjg.v20.i23.7197 PMC4064064 24966589

[31] Zhang ZZ, Chen J, He Y, Zhan X, Zhao RQ, Huang YF, et al miR-125b inhibits hepatitis B virus expression in vitro through targeting of the SCNN1A gene. Arch Virol. 2014;159: 3335–43. doi: 10.1007/s00705-014-2208-y 25173609 10.1007/s00705-014-2208-y

[32] Poy MN, Eliasson L, Krutzfeldt J, Kuwajima S, Ma X, Macdonald PE, et al A pancreatic islet-specific microRNA regulates insulin secretion. Nature. 2004;432: 226–30. doi: 10.1038/nature03076 15538371 10.1038/nature03076

[33] Ortega FJ, Mercader JM, Moreno-Navarrete JM, Rovira O, Guerra E, Esteve E, et al Profiling of Circulating MicroRNAs Reveals Common MicroRNAs Linked to Type 2 Diabetes That Change With Insulin Sensitization. Diabetes Care. 2014;37: 1375–83. doi: 10.2337/dc13-1847 24478399 10.2337/dc13-1847

[34] Cool B, Zinker B, Chiou W, Kifle L, Cao N, Perham M, et al Identification and characterization of a small molecule AMPK activator that treats key components of type 2 diabetes and the metabolic syndrome. Cell Metab. 2006;3: 403–16. doi: 10.1016/j.cmet.2006.05.005 16753576 10.1016/j.cmet.2006.05.005

[35] Saltiel AR, Kahn CR. Insulin signalling and the regulation of glucose and lipid metabolism. Nature. 2001;414: 799–806. doi: 10.1038/414799a 11742412 10.1038/414799a

[36] Milich DR, Chen M, Schodel F, Peterson DL, Jones JE, Hughes JL. Role of B cells in antigen presentation of the hepatitis B core. Proceedings of the National Academy of Sciences of the United States of America. 1997;94: 14648–53. 9405667 10.1073/pnas.94.26.14648 PMC25082

[37] Christen V, Duong F, Bernsmeier C, Sun D, Nassal M, Heim MH. Inhibition of alpha interferon signaling by hepatitis B virus. J Virol. 2007;81: 159–65. doi: 10.1128/JVI.01292-06 17065208 10.1128/JVI.01292-06 PMC1797249

[38] Du K, Ramachandran A, Jaeschke H. Oxidative stress during acetaminophen hepatotoxicity: Sources, pathophysiological role and therapeutic potential. Redox Biol. 2016;10: 148–56. doi: 10.1016/j.redox.2016.10.001 27744120 10.1016/j.redox.2016.10.001 PMC5065645

[39] Jaeschke H, McGill MR, Ramachandran A. Oxidant stress, mitochondria, and cell death mechanisms in drug-induced liver injury: lessons learned from acetaminophen hepatotoxicity. Drug Metab Rev. 2012;44: 88–106. doi: 10.3109/03602532.2011.602688 22229890 10.3109/03602532.2011.602688 PMC5319847

[40] Lee SJ, Kim KH, Park KK. Mechanisms of fibrogenesis in liver cirrhosis: The molecular aspects of epithelial-mesenchymal transition. World J Hepatol. 2014;6: 207–16. doi: 10.4254/wjh.v6.i4.207 24799989 10.4254/wjh.v6.i4.207 PMC4009476

[41] Preheim LC, Gentry MJ, Snitily MU. Pulmonary recruitment, adherence, and chemotaxis of neutrophils in a rat model of cirrhosis and pneumococcal pneumonia. The Journal of infectious diseases. 1991;164: 1203–6. 1955720 10.1093/infdis/164.6.1203

[42] Turchinovich A, Weiz L, Burwinkel B. Extracellular miRNAs: the mystery of their origin and function. Trends Biochem Sci. 2012;37: 460–5. doi: 10.1016/j.tibs.2012.08.003 22944280 10.1016/j.tibs.2012.08.003

[43] Vickers KC, Palmisano BT, Shoucri BM, Shamburek RD, Remaley AT. MicroRNAs are transported in plasma and delivered to recipient cells by high-density lipoproteins. Nat Cell Biol. 2011;13: 423–U182. doi: 10.1038/ncb2210 21423178 10.1038/ncb2210 PMC3074610

[44] Katoh T, Sakaguchi Y, Miyauchi K, Suzuki T, Kashiwabara S, Baba T, et al Selective stabilization of mammalian microRNAs by 3' adenylation mediated by the cytoplasmic poly(A) polymerase GLD-2. Genes Dev. 2009;23: 433–8. doi: 10.1101/gad.1761509 19240131 10.1101/gad.1761509 PMC2648654

[45] Heo I, Ha M, Lim J, Yoon MJ, Park JE, Kwon SC, et al Mono-Uridylation of Pre-MicroRNA as a Key Step in the Biogenesis of Group II let-7 MicroRNAs. Cell. 2012;151: 521–32. doi: 10.1016/j.cell.2012.09.022 23063654 10.1016/j.cell.2012.09.022

