# Supplementary material for: Serum microRNA signatures as "liquid biopsies" for interrogating hepatotoxic mechanisms and liver pathogenesis in human
Source: PLoS One. 2017 May 17;12(5):e0177928. doi: 10.1371/journal.pone.0177928 (PMC5435338; doi:10.1371/journal.pone.0177928)
Supplement: S1 Table — The table lists all subject including metadata and clinical chemistry paramenters. (DOCX) [file pone.0177928.s001.docx]

| **Diagnosis** | **Patient** | **Age** | **Gender** | **Ethnicity** | **ALT** | **AST** | **Tbil** | **Fibrosis score**  **(APRI)** |
| --- | --- | --- | --- | --- | --- | --- | --- | --- |
| LC | 1 | 40 | m | american indian | 8 | 28 | 6.7 | F4 |
|  | 2 | 60 | f | caucasian | 55 | 119 | 27 | F4 |
|  | 3 | 57 | m | caucasian | 34 | 82 | 0.7 | F4 |
|  | 4 | 45 | m | caucasian | 23 | 64 | 4.1 | F4 |
|  | 5 | 50 | m | african american | 130 | 82 | 0.2 | F3 |
|  | 6 | 45 | m | asian | 74 | 41 | 1.0 | F3 |
|  | 7 | 46 | m | asian | 25 | 18 | 1.4 | F0–F2 |
|  | 8 | 33 | m | caucasian | 24 | 51 | 9.7 | F3 |
| T2DM | 9 | 43 | m | caucasian | 44 | 29 | 1.2 | F3 |
|  | 10 | 59 | f | caucasian | 18 | 18 | 0.6 | F0–F2 |
|  | 11 | 60 | m | caucasian | unknown | unknown | unknown | F0–F2 |
|  | 12 | 26 | m | asian | 25 | 18 | 0.4 | F0–F2 |
|  | 13 | 58 | m | caucasian | 44 | 28 | 0.6 | F3 |
|  | 14 | 72 | m | caucasian | 36 | 30 | 0.7 | F0–F2 |
|  | 15 | 44 | f | asian | 15 | 18 | 0.4 | F0–F2 |
| HepB | 16 | 32 | f | asian | 19 | 22 | 0.3 | F0–F2 |
|  | 17 | 49 | m | caucasian | 71 | 32 | 0.5 | F0–F2 |
|  | 18 | 31 | m | unknown | 14 | 15 | 0.9 | F0–F2 |
|  | 19 | 30 | m | asian | 26 | 26 | 0.3 | F0–F2 |
|  | 20 | 26 | f | asian | 32 | 27 | 0.2 | F0–F2 |
|  | 21 | 50 | m | caucasian | 27 | 20 | 0.7 | F0–F2 |
|  | 22 | 38 | m | caucasian | 51 | 44 | 0.8 | F3 |

| **Diagnosis** | **Patient** | **Age** | **Gender** | **Ethnicity** | **ALT** | **AST** | **Tbil** | **Fibrosis score**  **(APRI)** |
| --- | --- | --- | --- | --- | --- | --- | --- | --- |
| HV | 23 | 52 | f | hispanic | ref range | ref range | ref range | N.A. |
|  | 24 | 37 | m | black | ref range | ref range | ref range | N.A. |
|  | 25 | 33 | m | black | ref range | ref range | ref range | N.A. |
|  | 26 | 25 | f | black | ref range | ref range | ref range | N.A. |
|  | 27 | 29 | m | other | ref range | ref range | ref range | N.A. |
|  | 28 | 42 | m | caucasian | ref range | ref range | ref range | N.A. |
|  | 29 | 29 | m | black | ref range | ref range | ref range | N.A. |
|  | 30 | 23 | f | black | ref range | ref range | ref range | N.A. |
|  | 31 | 13 | f | caucasian | 11 | 18 | 0.4 | F0–F2 |
|  | 32 | 64 | m | caucasian | 23 | 31 | 0.8 | F0–F2 |
|  | 33 | 31 | f | asian | 12 | 19 | 0.6 | F0–F2 |
|  | 34 | 25 | f | asian | 17 | 17 | 1.4 | F0–F2 |
|  | 35 | 40 | f | asian | 14 | 20 | 1.3 | F0–F2 |
|  | 36 | 35 | m | asian | 24 | 23 | 0.7 | F0–F2 |
|  | 37 | 42 | m | asian | 24 | 27 | 1.3 | F0–F2 |
|  | 38 | 62 | m | asian | 21 | 28 | 0.9 | F3 |
|  | 39 | 36 | f | caucasian | 22 | 19 | 0.3 | F0–F2 |
|  | 40 | 28 | f | caucasian | 16 | 17 | 0.3 | F0–F2 |
|  | 41 | 38 | f | unknown | 13 | 19 | 0.9 | F0–F2 |
|  | 42 | 43 | m | caucasian | 24 | 22 | 0.6 | F0–F2 |
|  | 43 | 21 | m | caucasian | 15 | 17 | 0.6 | F0–F2 |
|  | 44 | 32 | m | caucasian | 18 | 18 | 0.3 | F0–F2 |
| APAP | 45 | 37 | f | caucasian | 458 | 790 | 1.3 | Not applicable |
|  | 46 | 62 | f | caucasian | 3,057 | 2,335 | 3.3 | Not applicable |
|  | 47 | 14 | f | caucasian | 16 | 30 | 0.4 | Not applicable |
|  | 48 | 30 | m | caucasian | 5,410 | 5,810 | 0.6 | Not applicable |
|  | 49 | 24 | m | unknown | 8,941 | 8,707 | 3.2 | Not applicable |
|  | 50 | 29 | f | unknown | 3,196 | 12,105 | 5.1 | Not applicable |
|  | 51 | 43 | m | caucasian | 4,506 | 2,012 | 0.9 | Not applicable |
|  | 52 | 35 | f | caucasian | 3,541 | 4,654 | 4 | Not applicable |
|  | 53 | 35 | f | unknown | 10,363 | 10,208 | 1.3 | Not applicable |
