# Supplementary material for: Serum microRNA signatures as "liquid biopsies" for interrogating hepatotoxic mechanisms and liver pathogenesis in human
Source: PLoS One. 2017 May 17;12(5):e0177928. doi: 10.1371/journal.pone.0177928 (PMC5435338; doi:10.1371/journal.pone.0177928)
Supplement: S2 Table — Complete circulating miRNAs detected in the serum of all subjects. (DOCX) [file pone.0177928.s002.docx]

| miRNA |
| --- |
| hsa-let-7a-3p |
| hsa-let-7a-5p |
| hsa-let-7b-5p |
| hsa-let-7b-3p |
| hsa-let-7c-3p |
| hsa-let-7c-5p |
| hsa-let-7d-3p |
| hsa-let-7d-5p |
| hsa-let-7e-3p |
| hsa-let-7e-5p |
| hsa-let-7f-5p |
| hsa-let-7f-1-3p |
| hsa-let-7f-2-3p |
| hsa-let-7g-5p |
| hsa-let-7g-3p |
| hsa-let-7i-3p |
| hsa-let-7i-5p |
| hsa-miR-100-5p |
| hsa-miR-101-5p |
| hsa-miR-101-3p |
| hsa-miR-103a-3p |
| hsa-miR-103a-2-5p |
| hsa-miR-106a-3p |
| hsa-miR-106a-5p |
| hsa-miR-106b-5p |
| hsa-miR-106b-3p |
| hsa-miR-107 |
| hsa-miR-10a-5p |
| hsa-miR-10a-3p |
| hsa-miR-10b-5p |
| hsa-miR-10b-3p |
| hsa-miR-1180-3p |
| hsa-miR-1185-5p |
| hsa-miR-1185-1-3p |
| hsa-miR-1185-2-3p |
| hsa-miR-1197 |
| hsa-miR-122-3p |
| hsa-miR-122-5p |
| hsa-miR-1224-5p |
| hsa-miR-1226-3p |
| hsa-miR-1228-3p |
| hsa-miR-1229-3p |
| hsa-miR-1234-3p |
| hsa-miR-1246 |
| hsa-miR-1247-5p |
| hsa-miR-1249-3p |
| hsa-miR-1254 |
| hsa-miR-1255a |
| hsa-miR-1255b-5p |
| hsa-miR-1256 |
| hsa-miR-125a-5p |
| hsa-miR-125a-3p |
| hsa-miR-125b-5p |
| hsa-miR-125b-1-3p |
| hsa-miR-125b-2-3p |
| hsa-miR-126-5p |
| hsa-miR-126-3p |
| hsa-miR-1260a |
| hsa-miR-1260b |
| hsa-miR-1262 |
| hsa-miR-1266-5p |
| hsa-miR-127-3p |
| hsa-miR-127-5p |
| hsa-miR-1270 |
| hsa-miR-1271-5p |
| hsa-miR-1273d |
| hsa-miR-1273f |
| hsa-miR-1273g-3p |
| hsa-miR-1273h-5p |
| hsa-miR-1273h-3p |
| hsa-miR-1275 |
| hsa-miR-1276 |
| hsa-miR-1277-5p |
| hsa-miR-1277-3p |
| hsa-miR-1278 |
| hsa-miR-128-1-5p |
| hsa-miR-128-3p |
| hsa-miR-1283 |
| hsa-miR-1284 |
| hsa-miR-1285-3p |
| hsa-miR-1285-5p |
| hsa-miR-1287-5p |
| hsa-miR-1290 |
| hsa-miR-1291 |
| hsa-miR-1292-5p |
| hsa-miR-1294 |
| hsa-miR-1295b-5p |
| hsa-miR-1296-5p |
| hsa-miR-1299 |
| hsa-miR-1301-3p |
| hsa-miR-1303 |
| hsa-miR-1304-3p |
| hsa-miR-1304-5p |
| hsa-miR-1306-5p |
| hsa-miR-1306-3p |
| hsa-miR-1307-5p |
| hsa-miR-1307-3p |
| hsa-miR-130a-3p |
| hsa-miR-130b-5p |
| hsa-miR-130b-3p |
| hsa-miR-132-3p |
| hsa-miR-132-5p |
| hsa-miR-1323 |
| hsa-miR-133a-3p |
| hsa-miR-133b |
| hsa-miR-134-5p |
| hsa-miR-134-3p |
| hsa-miR-1343-3p |
| hsa-miR-135a-3p |
| hsa-miR-136-3p |
| hsa-miR-136-5p |
| hsa-miR-138-5p |
| hsa-miR-139-3p |
| hsa-miR-139-5p |
| hsa-miR-140-5p |
| hsa-miR-140-3p |
| hsa-miR-141-3p |
| hsa-miR-142-5p |
| hsa-miR-142-3p |
| hsa-miR-143-3p |
| hsa-miR-143-5p |
| hsa-miR-144-3p |
| hsa-miR-144-5p |
| hsa-miR-145-5p |
| hsa-miR-145-3p |
| hsa-miR-1468-5p |
| hsa-miR-146a-5p |
| hsa-miR-146a-3p |
| hsa-miR-146b-3p |
| hsa-miR-146b-5p |
| hsa-miR-148a-3p |
| hsa-miR-148a-5p |
| hsa-miR-148b-5p |
| hsa-miR-148b-3p |
| hsa-miR-149-5p |
| hsa-miR-150-5p |
| hsa-miR-150-3p |
| hsa-miR-151a-5p |
| hsa-miR-151a-3p |
| hsa-miR-151b |
| hsa-miR-152-5p |
| hsa-miR-152-3p |
| hsa-miR-153-3p |
| hsa-miR-1537-3p |
| hsa-miR-1538 |
| hsa-miR-154-5p |
| hsa-miR-155-5p |
| hsa-miR-15a-5p |
| hsa-miR-15a-3p |
| hsa-miR-15b-3p |
| hsa-miR-15b-5p |
| hsa-miR-16-1-3p |
| hsa-miR-16-5p |
| hsa-miR-16-2-3p |
| hsa-miR-17-5p |
| hsa-miR-17-3p |
| hsa-miR-181a-3p |
| hsa-miR-181a-5p |
| hsa-miR-181a-2-3p |
| hsa-miR-181b-3p |
| hsa-miR-181b-5p |
| hsa-miR-181b-2-3p |
| hsa-miR-181c-3p |
| hsa-miR-181c-5p |
| hsa-miR-181d-3p |
| hsa-miR-181d-5p |
| hsa-miR-182-5p |
| hsa-miR-183-3p |
| hsa-miR-183-5p |
| hsa-miR-184 |
| hsa-miR-185-5p |
| hsa-miR-185-3p |
| hsa-miR-186-5p |
| hsa-miR-186-3p |
| hsa-miR-187-3p |
| hsa-miR-188-5p |
| hsa-miR-18a-5p |
| hsa-miR-18a-3p |
| hsa-miR-18b-5p |
| hsa-miR-18b-3p |
| hsa-miR-1908-5p |
| hsa-miR-1908-3p |
| hsa-miR-190a-5p |
| hsa-miR-190b |
| hsa-miR-191-3p |
| hsa-miR-191-5p |
| hsa-miR-1910-5p |
| hsa-miR-1914-5p |
| hsa-miR-192-5p |
| hsa-miR-193a-3p |
| hsa-miR-193a-5p |
| hsa-miR-193b-3p |
| hsa-miR-193b-5p |
| hsa-miR-194-5p |
| hsa-miR-194-3p |
| hsa-miR-195-3p |
| hsa-miR-195-5p |
| hsa-miR-196b-5p |
| hsa-miR-196b-3p |
| hsa-miR-197-5p |
| hsa-miR-197-3p |
| hsa-miR-1976 |
| hsa-miR-199a-3p |
| hsa-miR-199a-5p |
| hsa-miR-199b-5p |
| hsa-miR-199b-3p |
| hsa-miR-19a-3p |
| hsa-miR-19a-5p |
| hsa-miR-19b-3p |
| hsa-miR-200a-3p |
| hsa-miR-200b-3p |
| hsa-miR-200c-3p |
| hsa-miR-202-3p |
| hsa-miR-203a-3p |
| hsa-miR-204-5p |
| hsa-miR-204-3p |
| hsa-miR-205-5p |
| hsa-miR-208b-3p |
| hsa-miR-20a-3p |
| hsa-miR-20a-5p |
| hsa-miR-20b-5p |
| hsa-miR-20b-3p |
| hsa-miR-21-5p |
| hsa-miR-21-3p |
| hsa-miR-210-3p |
| hsa-miR-210-5p |
| hsa-miR-2110 |
| hsa-miR-2114-3p |
| hsa-miR-2115-3p |
| hsa-miR-2115-5p |
| hsa-miR-2116-3p |
| hsa-miR-212-3p |
| hsa-miR-212-5p |
| hsa-miR-214-5p |
| hsa-miR-214-3p |
| hsa-miR-215-5p |
| hsa-miR-219a-1-3p |
| hsa-miR-219b-5p |
| hsa-miR-22-3p |
| hsa-miR-22-5p |
| hsa-miR-221-3p |
| hsa-miR-221-5p |
| hsa-miR-222-5p |
| hsa-miR-222-3p |
| hsa-miR-223-3p |
| hsa-miR-223-5p |
| hsa-miR-224-5p |
| hsa-miR-224-3p |
| hsa-miR-2276-3p |
| hsa-miR-2277-3p |
| hsa-miR-2277-5p |
| hsa-miR-2355-5p |
| hsa-miR-2355-3p |
| hsa-miR-23a-3p |
| hsa-miR-23a-5p |
| hsa-miR-23b-3p |
| hsa-miR-23b-5p |
| hsa-miR-24-3p |
| hsa-miR-24-2-5p |
| hsa-miR-2467-5p |
| hsa-miR-25-5p |
| hsa-miR-25-3p |
| hsa-miR-26a-5p |
| hsa-miR-26a-1-3p |
| hsa-miR-26a-2-3p |
| hsa-miR-26b-5p |
| hsa-miR-26b-3p |
| hsa-miR-27a-5p |
| hsa-miR-27a-3p |
| hsa-miR-27b-5p |
| hsa-miR-27b-3p |
| hsa-miR-28-3p |
| hsa-miR-28-5p |
| hsa-miR-296-5p |
| hsa-miR-299-3p |
| hsa-miR-29a-5p |
| hsa-miR-29a-3p |
| hsa-miR-29b-3p |
| hsa-miR-29b-2-5p |
| hsa-miR-29c-3p |
| hsa-miR-29c-5p |
| hsa-miR-301a-5p |
| hsa-miR-301a-3p |
| hsa-miR-301b-3p |
| hsa-miR-3064-5p |
| hsa-miR-3065-3p |
| hsa-miR-3065-5p |
| hsa-miR-3074-5p |
| hsa-miR-3074-3p |
| hsa-miR-30a-5p |
| hsa-miR-30a-3p |
| hsa-miR-30b-5p |
| hsa-miR-30b-3p |
| hsa-miR-30c-5p |
| hsa-miR-30c-1-3p |
| hsa-miR-30c-2-3p |
| hsa-miR-30d-3p |
| hsa-miR-30d-5p |
| hsa-miR-30e-3p |
| hsa-miR-30e-5p |
| hsa-miR-31-5p |
| hsa-miR-3120-3p |
| hsa-miR-3120-5p |
| hsa-miR-3121-3p |
| hsa-miR-3122 |
| hsa-miR-3124-5p |
| hsa-miR-3127-5p |
| hsa-miR-3127-3p |
| hsa-miR-3130-5p |
| hsa-miR-3135a |
| hsa-miR-3136-5p |
| hsa-miR-3138 |
| hsa-miR-3143 |
| hsa-miR-3150a-5p |
| hsa-miR-3150b-3p |
| hsa-miR-3157-3p |
| hsa-miR-3157-5p |
| hsa-miR-3158-3p |
| hsa-miR-3159 |
| hsa-miR-3161 |
| hsa-miR-3163 |
| hsa-miR-3166 |
| hsa-miR-3173-5p |
| hsa-miR-3173-3p |
| hsa-miR-3174 |
| hsa-miR-3176 |
| hsa-miR-3177-3p |
| hsa-miR-3186-3p |
| hsa-miR-3187-3p |
| hsa-miR-3188 |
| hsa-miR-3190-3p |
| hsa-miR-3191-3p |
| hsa-miR-3196 |
| hsa-miR-32-5p |
| hsa-miR-32-3p |
| hsa-miR-3200-5p |
| hsa-miR-3200-3p |
| hsa-miR-320a |
| hsa-miR-320b |
| hsa-miR-320c |
| hsa-miR-320d |
| hsa-miR-323a-3p |
| hsa-miR-323b-3p |
| hsa-miR-324-3p |
| hsa-miR-324-5p |
| hsa-miR-326 |
| hsa-miR-328-3p |
| hsa-miR-329-5p |
| hsa-miR-329-3p |
| hsa-miR-330-3p |
| hsa-miR-330-5p |
| hsa-miR-331-3p |
| hsa-miR-331-5p |
| hsa-miR-335-3p |
| hsa-miR-335-5p |
| hsa-miR-337-5p |
| hsa-miR-337-3p |
| hsa-miR-338-5p |
| hsa-miR-338-3p |
| hsa-miR-339-5p |
| hsa-miR-339-3p |
| hsa-miR-33a-5p |
| hsa-miR-33a-3p |
| hsa-miR-33b-5p |
| hsa-miR-340-3p |
| hsa-miR-340-5p |
| hsa-miR-342-3p |
| hsa-miR-342-5p |
| hsa-miR-345-5p |
| hsa-miR-34a-5p |
| hsa-miR-34c-5p |
| hsa-miR-3591-5p |
| hsa-miR-3605-5p |
| hsa-miR-3605-3p |
| hsa-miR-3607-3p |
| hsa-miR-361-3p |
| hsa-miR-361-5p |
| hsa-miR-3613-5p |
| hsa-miR-3613-3p |
| hsa-miR-3614-5p |
| hsa-miR-3615 |
| hsa-miR-3617-3p |
| hsa-miR-3617-5p |
| hsa-miR-362-3p |
| hsa-miR-362-5p |
| hsa-miR-363-3p |
| hsa-miR-3653-3p |
| hsa-miR-3656 |
| hsa-miR-365a-3p |
| hsa-miR-365b-3p |
| hsa-miR-365b-5p |
| hsa-miR-3661 |
| hsa-miR-3675-5p |
| hsa-miR-3677-3p |
| hsa-miR-3684 |
| hsa-miR-3688-3p |
| hsa-miR-369-5p |
| hsa-miR-369-3p |
| hsa-miR-3690 |
| hsa-miR-3691-5p |
| hsa-miR-370-3p |
| hsa-miR-370-5p |
| hsa-miR-371b-3p |
| hsa-miR-371b-5p |
| hsa-miR-374a-3p |
| hsa-miR-374a-5p |
| hsa-miR-374b-3p |
| hsa-miR-374b-5p |
| hsa-miR-375 |
| hsa-miR-376a-5p |
| hsa-miR-376a-3p |
| hsa-miR-376b-3p |
| hsa-miR-376c-3p |
| hsa-miR-377-5p |
| hsa-miR-377-3p |
| hsa-miR-378a-5p |
| hsa-miR-378a-3p |
| hsa-miR-378c |
| hsa-miR-378d |
| hsa-miR-378f |
| hsa-miR-378g |
| hsa-miR-378i |
| hsa-miR-379-5p |
| hsa-miR-379-3p |
| hsa-miR-380-3p |
| hsa-miR-381-3p |
| hsa-miR-382-3p |
| hsa-miR-382-5p |
| hsa-miR-3909 |
| hsa-miR-3912-3p |
| hsa-miR-3913-5p |
| hsa-miR-3916 |
| hsa-miR-3918 |
| hsa-miR-3920 |
| hsa-miR-3928-3p |
| hsa-miR-3938 |
| hsa-miR-3939 |
| hsa-miR-3940-3p |
| hsa-miR-3942-5p |
| hsa-miR-3960 |
| hsa-miR-409-5p |
| hsa-miR-409-3p |
| hsa-miR-410-3p |
| hsa-miR-411-5p |
| hsa-miR-411-3p |
| hsa-miR-412-5p |
| hsa-miR-421 |
| hsa-miR-423-5p |
| hsa-miR-423-3p |
| hsa-miR-424-3p |
| hsa-miR-424-5p |
| hsa-miR-425-3p |
| hsa-miR-425-5p |
| hsa-miR-4286 |
| hsa-miR-429 |
| hsa-miR-431-3p |
| hsa-miR-431-5p |
| hsa-miR-432-3p |
| hsa-miR-432-5p |
| hsa-miR-4326 |
| hsa-miR-433-3p |
| hsa-miR-433-5p |
| hsa-miR-4422 |
| hsa-miR-4433b-5p |
| hsa-miR-4433b-3p |
| hsa-miR-4435 |
| hsa-miR-4440 |
| hsa-miR-4446-5p |
| hsa-miR-4446-3p |
| hsa-miR-4448 |
| hsa-miR-4449 |
| hsa-miR-4454 |
| hsa-miR-4466 |
| hsa-miR-4467 |
| hsa-miR-4482-3p |
| hsa-miR-4485-3p |
| hsa-miR-4492 |
| hsa-miR-449c-5p |
| hsa-miR-4507 |
| hsa-miR-4508 |
| hsa-miR-450a-5p |
| hsa-miR-450a-2-3p |
| hsa-miR-450b-5p |
| hsa-miR-4511 |
| hsa-miR-4516 |
| hsa-miR-451a |
| hsa-miR-452-5p |
| hsa-miR-4523 |
| hsa-miR-4526 |
| hsa-miR-4532 |
| hsa-miR-454-5p |
| hsa-miR-454-3p |
| hsa-miR-455-3p |
| hsa-miR-455-5p |
| hsa-miR-4638-3p |
| hsa-miR-4645-3p |
| hsa-miR-4646-5p |
| hsa-miR-4647 |
| hsa-miR-4657 |
| hsa-miR-4659b-3p |
| hsa-miR-4660 |
| hsa-miR-4661-5p |
| hsa-miR-4662a-5p |
| hsa-miR-4664-3p |
| hsa-miR-4665-5p |
| hsa-miR-4672 |
| hsa-miR-4676-3p |
| hsa-miR-4677-3p |
| hsa-miR-4685-3p |
| hsa-miR-4687-5p |
| hsa-miR-4688 |
| hsa-miR-4701-5p |
| hsa-miR-4707-3p |
| hsa-miR-4714-5p |
| hsa-miR-4714-3p |
| hsa-miR-4732-3p |
| hsa-miR-4732-5p |
| hsa-miR-4738-3p |
| hsa-miR-4741 |
| hsa-miR-4742-5p |
| hsa-miR-4742-3p |
| hsa-miR-4745-5p |
| hsa-miR-4746-5p |
| hsa-miR-4748 |
| hsa-miR-4751 |
| hsa-miR-4753-5p |
| hsa-miR-4755-5p |
| hsa-miR-4755-3p |
| hsa-miR-4757-3p |
| hsa-miR-4762-5p |
| hsa-miR-4767 |
| hsa-miR-4772-3p |
| hsa-miR-4772-5p |
| hsa-miR-4773 |
| hsa-miR-4775 |
| hsa-miR-4777-3p |
| hsa-miR-4781-3p |
| hsa-miR-4785 |
| hsa-miR-4786-5p |
| hsa-miR-4792 |
| hsa-miR-4796-3p |
| hsa-miR-4797-3p |
| hsa-miR-483-3p |
| hsa-miR-483-5p |
| hsa-miR-484 |
| hsa-miR-485-5p |
| hsa-miR-485-3p |
| hsa-miR-486-3p |
| hsa-miR-486-5p |
| hsa-miR-487a-3p |
| hsa-miR-487a-5p |
| hsa-miR-487b-3p |
| hsa-miR-487b-5p |
| hsa-miR-490-3p |
| hsa-miR-491-5p |
| hsa-miR-493-3p |
| hsa-miR-493-5p |
| hsa-miR-494-5p |
| hsa-miR-494-3p |
| hsa-miR-495-3p |
| hsa-miR-496 |
| hsa-miR-497-5p |
| hsa-miR-499a-5p |
| hsa-miR-5001-3p |
| hsa-miR-5006-3p |
| hsa-miR-500a-3p |
| hsa-miR-500a-5p |
| hsa-miR-500b-5p |
| hsa-miR-501-3p |
| hsa-miR-501-5p |
| hsa-miR-5010-5p |
| hsa-miR-5010-3p |
| hsa-miR-502-3p |
| hsa-miR-502-5p |
| hsa-miR-503-5p |
| hsa-miR-504-5p |
| hsa-miR-505-3p |
| hsa-miR-505-5p |
| hsa-miR-509-3p |
| hsa-miR-5094 |
| hsa-miR-5096 |
| hsa-miR-5100 |
| hsa-miR-511-5p |
| hsa-miR-512-3p |
| hsa-miR-516b-5p |
| hsa-miR-5187-5p |
| hsa-miR-5187-3p |
| hsa-miR-5189-3p |
| hsa-miR-5189-5p |
| hsa-miR-5193 |
| hsa-miR-520a-3p |
| hsa-miR-532-3p |
| hsa-miR-532-5p |
| hsa-miR-539-3p |
| hsa-miR-539-5p |
| hsa-miR-542-3p |
| hsa-miR-542-5p |
| hsa-miR-543 |
| hsa-miR-545-5p |
| hsa-miR-548a-3p |
| hsa-miR-548a-5p |
| hsa-miR-548ab |
| hsa-miR-548ac |
| hsa-miR-548ad-5p |
| hsa-miR-548ae-5p |
| hsa-miR-548ah-3p |
| hsa-miR-548aj-5p |
| hsa-miR-548al |
| hsa-miR-548am-5p |
| hsa-miR-548ap-5p |
| hsa-miR-548aq-3p |
| hsa-miR-548ar-5p |
| hsa-miR-548at-5p |
| hsa-miR-548au-5p |
| hsa-miR-548ax |
| hsa-miR-548ay-5p |
| hsa-miR-548ay-3p |
| hsa-miR-548az-5p |
| hsa-miR-548b-5p |
| hsa-miR-548c-5p |
| hsa-miR-548c-3p |
| hsa-miR-548d-3p |
| hsa-miR-548d-5p |
| hsa-miR-548e-3p |
| hsa-miR-548e-5p |
| hsa-miR-548f-5p |
| hsa-miR-548g-5p |
| hsa-miR-548h-5p |
| hsa-miR-548h-3p |
| hsa-miR-548j-5p |
| hsa-miR-548j-3p |
| hsa-miR-548k |
| hsa-miR-548l |
| hsa-miR-548n |
| hsa-miR-548o-3p |
| hsa-miR-548o-5p |
| hsa-miR-548p |
| hsa-miR-548q |
| hsa-miR-548u |
| hsa-miR-548w |
| hsa-miR-548x-5p |
| hsa-miR-548z |
| hsa-miR-550a-5p |
| hsa-miR-550a-3-5p |
| hsa-miR-550a-3p |
| hsa-miR-550b-3p |
| hsa-miR-551a |
| hsa-miR-551b-3p |
| hsa-miR-556-3p |
| hsa-miR-556-5p |
| hsa-miR-5581-3p |
| hsa-miR-5587-5p |
| hsa-miR-5588-5p |
| hsa-miR-561-5p |
| hsa-miR-5683 |
| hsa-miR-5695 |
| hsa-miR-570-3p |
| hsa-miR-5706 |
| hsa-miR-574-3p |
| hsa-miR-574-5p |
| hsa-miR-576-3p |
| hsa-miR-576-5p |
| hsa-miR-577 |
| hsa-miR-579-5p |
| hsa-miR-579-3p |
| hsa-miR-580-3p |
| hsa-miR-582-5p |
| hsa-miR-582-3p |
| hsa-miR-584-5p |
| hsa-miR-584-3p |
| hsa-miR-589-5p |
| hsa-miR-589-3p |
| hsa-miR-590-5p |
| hsa-miR-590-3p |
| hsa-miR-592 |
| hsa-miR-597-3p |
| hsa-miR-598-3p |
| hsa-miR-605-5p |
| hsa-miR-605-3p |
| hsa-miR-6087 |
| hsa-miR-610 |
| hsa-miR-615-3p |
| hsa-miR-616-5p |
| hsa-miR-616-3p |
| hsa-miR-618 |
| hsa-miR-619-5p |
| hsa-miR-624-3p |
| hsa-miR-624-5p |
| hsa-miR-625-3p |
| hsa-miR-625-5p |
| hsa-miR-627-3p |
| hsa-miR-627-5p |
| hsa-miR-628-5p |
| hsa-miR-628-3p |
| hsa-miR-629-3p |
| hsa-miR-629-5p |
| hsa-miR-636 |
| hsa-miR-641 |
| hsa-miR-642a-3p |
| hsa-miR-643 |
| hsa-miR-6500-3p |
| hsa-miR-6501-5p |
| hsa-miR-6502-5p |
| hsa-miR-6503-5p |
| hsa-miR-6503-3p |
| hsa-miR-6509-5p |
| hsa-miR-651-5p |
| hsa-miR-6511a-3p |
| hsa-miR-6511b-5p |
| hsa-miR-6511b-3p |
| hsa-miR-6513-3p |
| hsa-miR-6513-5p |
| hsa-miR-6514-5p |
| hsa-miR-6515-5p |
| hsa-miR-6516-5p |
| hsa-miR-6516-3p |
| hsa-miR-652-5p |
| hsa-miR-652-3p |
| hsa-miR-654-3p |
| hsa-miR-654-5p |
| hsa-miR-655-3p |
| hsa-miR-656-3p |
| hsa-miR-659-5p |
| hsa-miR-660-3p |
| hsa-miR-660-5p |
| hsa-miR-664a-3p |
| hsa-miR-664a-5p |
| hsa-miR-664b-5p |
| hsa-miR-664b-3p |
| hsa-miR-665 |
| hsa-miR-668-3p |
| hsa-miR-671-5p |
| hsa-miR-671-3p |
| hsa-miR-6715a-3p |
| hsa-miR-6716-3p |
| hsa-miR-6721-5p |
| hsa-miR-6730-3p |
| hsa-miR-6734-5p |
| hsa-miR-6735-5p |
| hsa-miR-6735-3p |
| hsa-miR-6740-5p |
| hsa-miR-6741-3p |
| hsa-miR-6747-3p |
| hsa-miR-675-3p |
| hsa-miR-6750-3p |
| hsa-miR-6754-3p |
| hsa-miR-6755-5p |
| hsa-miR-6762-3p |
| hsa-miR-6764-3p |
| hsa-miR-6764-5p |
| hsa-miR-6767-5p |
| hsa-miR-6770-3p |
| hsa-miR-6772-3p |
| hsa-miR-6775-3p |
| hsa-miR-6777-3p |
| hsa-miR-6780a-5p |
| hsa-miR-6783-5p |
| hsa-miR-6786-3p |
| hsa-miR-6802-3p |
| hsa-miR-6802-5p |
| hsa-miR-6803-3p |
| hsa-miR-6804-5p |
| hsa-miR-6809-5p |
| hsa-miR-6810-5p |
| hsa-miR-6813-5p |
| hsa-miR-6815-5p |
| hsa-miR-6816-3p |
| hsa-miR-6817-3p |
| hsa-miR-6818-5p |
| hsa-miR-6819-3p |
| hsa-miR-6820-5p |
| hsa-miR-6832-5p |
| hsa-miR-6837-3p |
| hsa-miR-6838-5p |
| hsa-miR-6840-5p |
| hsa-miR-6842-5p |
| hsa-miR-6842-3p |
| hsa-miR-6847-5p |
| hsa-miR-6850-5p |
| hsa-miR-6852-5p |
| hsa-miR-6855-3p |
| hsa-miR-6859-5p |
| hsa-miR-6861-5p |
| hsa-miR-6862-5p |
| hsa-miR-6866-5p |
| hsa-miR-6868-3p |
| hsa-miR-6875-5p |
| hsa-miR-6876-5p |
| hsa-miR-6881-3p |
| hsa-miR-6882-5p |
| hsa-miR-6884-5p |
| hsa-miR-6891-5p |
| hsa-miR-6892-5p |
| hsa-miR-6894-5p |
| hsa-miR-7-1-3p |
| hsa-miR-744-3p |
| hsa-miR-744-5p |
| hsa-miR-758-3p |
| hsa-miR-760 |
| hsa-miR-7641 |
| hsa-miR-766-3p |
| hsa-miR-766-5p |
| hsa-miR-769-5p |
| hsa-miR-769-3p |
| hsa-miR-7704 |
| hsa-miR-7705 |
| hsa-miR-7706 |
| hsa-miR-7848-3p |
| hsa-miR-7849-3p |
| hsa-miR-7850-5p |
| hsa-miR-7854-3p |
| hsa-miR-7855-5p |
| hsa-miR-7856-5p |
| hsa-miR-7976 |
| hsa-miR-7977 |
| hsa-miR-8061 |
| hsa-miR-873-5p |
| hsa-miR-873-3p |
| hsa-miR-874-3p |
| hsa-miR-874-5p |
| hsa-miR-877-5p |
| hsa-miR-885-5p |
| hsa-miR-885-3p |
| hsa-miR-887-3p |
| hsa-miR-889-5p |
| hsa-miR-889-3p |
| hsa-miR-9-5p |
| hsa-miR-92a-1-5p |
| hsa-miR-92a-3p |
| hsa-miR-92b-5p |
| hsa-miR-92b-3p |
| hsa-miR-93-5p |
| hsa-miR-93-3p |
| hsa-miR-933 |
| hsa-miR-937-3p |
| hsa-miR-939-3p |
| hsa-miR-939-5p |
| hsa-miR-940 |
| hsa-miR-941 |
| hsa-miR-942-3p |
| hsa-miR-942-5p |
| hsa-miR-943 |
| hsa-miR-95-3p |
| hsa-miR-96-5p |
| hsa-miR-98-3p |
| hsa-miR-98-5p |
| hsa-miR-99a-3p |
| hsa-miR-99a-5p |
| hsa-miR-99b-5p |
| hsa-miR-99b-3p |
