# Supplementary material for: Serum microRNA signatures as "liquid biopsies" for interrogating hepatotoxic mechanisms and liver pathogenesis in human
Source: PLoS One. 2017 May 17;12(5):e0177928. doi: 10.1371/journal.pone.0177928 (PMC5435338; doi:10.1371/journal.pone.0177928)
Supplement: S3 Table — List of all miRNAs and its presence in serum in percent. (DOCX) [file pone.0177928.s003.docx]

| miRNA | APAP (%) | HBV (%) | LC (%) | T2DM (%) | HC (%) |
| --- | --- | --- | --- | --- | --- |
| hsa-miR-486-5p | 22.36 | 44.84 | 46.85 | 36.43 | 39.78 |
| hsa-miR-22-3p | 20.59 | 6.04 | 7.61 | 8.8 | 6.87 |
| hsa-miR-92a-3p | 8.33 | 12.61 | 12.58 | 8.12 | 11.41 |
| hsa-miR-451a | 5.31 | 4.77 | 4.44 | 4.53 | 6.08 |
| hsa-miR-27b-3p | 4.42 | 0.48 | 0.33 | 0.45 | 0.55 |
| hsa-miR-423-5p | 4.27 | 3.94 | 6.52 | 5.92 | 4.68 |
| hsa-miR-320a | 2.61 | 0.24 | 0.34 | 0.24 | 0.24 |
| hsa-miR-16-5p | 2.49 | 2.75 | 2.73 | 2.52 | 3.2 |
| hsa-miR-148a-3p | 1.91 | 0.87 | 0.53 | 0.48 | 0.5 |
| hsa-miR-21-5p | 1.85 | 0.55 | 0.71 | 0.91 | 0.83 |
| hsa-miR-30d-5p | 1.42 | 1.56 | 1.09 | 2.04 | 1.41 |
| hsa-miR-320b | 1.41 | 0.02 | 0.08 | 0.03 | 0.03 |
| hsa-miR-103a-3p | 1.38 | 0.25 | 0.21 | 0.41 | 0.33 |
| hsa-miR-107 | 1.36 | 0.22 | 0.18 | 0.3 | 0.28 |
| hsa-miR-25-3p | 1.16 | 1.39 | 1.01 | 1.4 | 1.43 |
| hsa-miR-10a-5p | 1.1 | 0.42 | 0.5 | 0.38 | 0.7 |
| hsa-miR-10b-5p | 1.09 | 0.83 | 1 | 0.27 | 1.6 |
| hsa-miR-192-5p | 1.08 | 1.48 | 0.15 | 0.16 | 0.27 |
| hsa-miR-15a-5p | 1.04 | 0.64 | 0.9 | 0.83 | 0.87 |
| hsa-miR-30a-5p | 0.99 | 0.23 | 0.15 | 0.05 | 0.19 |
| hsa-miR-142-5p | 0.9 | 1.91 | 0.92 | 3.05 | 2.08 |
| hsa-miR-30e-5p | 0.8 | 0.92 | 0.83 | 1.18 | 1.09 |
| hsa-miR-130a-3p | 0.74 | 0.09 | 0.12 | 0.21 | 0.12 |
| hsa-miR-126-5p | 0.67 | 0.33 | 0.38 | 0.93 | 0.86 |
| hsa-miR-191-5p | 0.58 | 1.14 | 0.82 | 3.74 | 1.73 |
| hsa-miR-181a-5p | 0.5 | 0.64 | 0.64 | 0.64 | 0.83 |
| hsa-miR-423-3p | 0.4 | 0.37 | 0.32 | 0.73 | 0.41 |
| hsa-miR-378a-3p | 0.4 | 0.3 | 0.09 | 0.09 | 0.1 |
| hsa-miR-26a-5p | 0.38 | 0.5 | 0.37 | 1.92 | 0.81 |
| hsa-miR-186-5p | 0.37 | 0.54 | 0.49 | 0.55 | 0.6 |
| hsa-miR-130b-3p | 0.33 | 0.03 | 0.03 | 0.07 | 0.05 |
| hsa-let-7f-5p | 0.27 | 0.36 | 0.29 | 0.78 | 0.59 |
| hsa-miR-151a-3p | 0.27 | 0.34 | 0.19 | 0.79 | 0.43 |
| hsa-let-7i-5p | 0.26 | 0.25 | 0.24 | 0.54 | 0.45 |
| hsa-miR-100-5p | 0.26 | 0.06 | 0.03 | 0.01 | 0.02 |
| hsa-miR-484 | 0.23 | 0.39 | 0.8 | 0.57 | 0.39 |
| hsa-miR-27a-3p | 0.22 | 0.14 | 0.22 | 0.25 | 0.23 |
| hsa-miR-221-3p | 0.21 | 0.08 | 0.09 | 0.19 | 0.11 |
| hsa-miR-28-3p | 0.18 | 0.4 | 0.14 | 0.39 | 0.22 |
| hsa-miR-23a-3p | 0.18 | 0.06 | 0.07 | 0.1 | 0.08 |
| hsa-miR-21-3p | 0.18 | 0.03 | 0.06 | 0.04 | 0.06 |
| hsa-miR-425-5p | 0.17 | 0.29 | 0.27 | 0.29 | 0.33 |
| hsa-miR-143-3p | 0.17 | 0.12 | 0.12 | 0.22 | 0.2 |
| hsa-miR-1307-5p | 0.17 | 0.05 | 0.08 | 0.06 | 0.08 |
| hsa-miR-150-5p | 0.15 | 1.44 | 0.39 | 0.37 | 0.7 |
| hsa-miR-122-5p | 0.15 | 0.12 | 0.01 | 0 | 0 |
| hsa-miR-101-3p | 0.14 | 0.14 | 0.09 | 0.1 | 0.19 |
| hsa-miR-16-2-3p | 0.14 | 0.13 | 0.17 | 0.16 | 0.17 |
| hsa-miR-23b-3p | 0.14 | 0.03 | 0.01 | 0.02 | 0.02 |
| hsa-miR-363-3p | 0.13 | 0.13 | 0.12 | 0.17 | 0.15 |
| hsa-miR-99a-5p | 0.13 | 0.02 | 0.01 | 0 | 0.01 |
| hsa-miR-182-5p | 0.11 | 0.1 | 0.1 | 0.2 | 0.2 |
| hsa-let-7d-3p | 0.11 | 0.15 | 0.18 | 0.13 | 0.17 |
| hsa-miR-29a-3p | 0.11 | 0.1 | 0.05 | 0.07 | 0.08 |
| hsa-miR-140-3p | 0.1 | 0.21 | 0.14 | 0.23 | 0.21 |
| hsa-miR-151a-5p | 0.1 | 0.11 | 0.08 | 0.44 | 0.2 |
| hsa-miR-93-5p | 0.09 | 0.12 | 0.08 | 0.26 | 0.23 |
| hsa-let-7a-5p | 0.09 | 0.14 | 0.11 | 0.43 | 0.17 |
| hsa-miR-26b-5p | 0.09 | 0.1 | 0.07 | 0.24 | 0.16 |
| hsa-let-7b-5p | 0.09 | 0.1 | 0.1 | 0.25 | 0.14 |
| hsa-miR-375 | 0.09 | 0.16 | 0.06 | 0.02 | 0.1 |
| hsa-miR-92b-3p | 0.08 | 0.12 | 0.17 | 0.06 | 0.16 |
| hsa-miR-126-3p | 0.08 | 0.06 | 0.08 | 0.15 | 0.13 |
| hsa-miR-335-5p | 0.08 | 0.03 | 0.04 | 0.04 | 0.04 |
| hsa-miR-125b-2-3p | 0.08 | 0.01 | 0 | 0 | 0 |
| hsa-miR-144-3p | 0.07 | 0.05 | 0.05 | 0.06 | 0.15 |
| hsa-let-7g-5p | 0.07 | 0.1 | 0.06 | 0.18 | 0.14 |
| hsa-miR-99b-5p | 0.07 | 0.06 | 0.08 | 0.16 | 0.08 |
| hsa-miR-3615 | 0.07 | 0.08 | 0.11 | 0.05 | 0.08 |
| hsa-miR-652-3p | 0.07 | 0.04 | 0.06 | 0.08 | 0.05 |
| hsa-miR-210-3p | 0.07 | 0.02 | 0.02 | 0.02 | 0.02 |
| hsa-miR-223-3p | 0.06 | 0.16 | 0.11 | 0.4 | 0.17 |
| hsa-miR-146a-5p | 0.06 | 0.07 | 0.05 | 0.16 | 0.12 |
| hsa-miR-1246 | 0.06 | 0 | 0 | 0 | 0.01 |
| hsa-miR-6087 | 0.06 | 0 | 0 | 0 | 0 |
| hsa-miR-584-5p | 0.05 | 0.06 | 0.09 | 0.11 | 0.1 |
| hsa-miR-128-3p | 0.05 | 0.05 | 0.05 | 0.07 | 0.05 |
| hsa-miR-340-5p | 0.05 | 0.03 | 0.03 | 0.05 | 0.05 |
| hsa-miR-345-5p | 0.05 | 0.02 | 0.03 | 0.04 | 0.03 |
| hsa-miR-660-5p | 0.05 | 0.03 | 0.03 | 0.02 | 0.03 |
| hsa-miR-361-5p | 0.05 | 0.01 | 0.01 | 0.03 | 0.01 |
| hsa-miR-222-3p | 0.04 | 0.09 | 0.06 | 0.28 | 0.11 |
| hsa-let-7d-5p | 0.04 | 0.06 | 0.05 | 0.23 | 0.1 |
| hsa-miR-125a-5p | 0.04 | 0.08 | 0.07 | 0.12 | 0.09 |
| hsa-miR-144-5p | 0.04 | 0.04 | 0.04 | 0.04 | 0.08 |
| hsa-miR-106b-3p | 0.04 | 0.06 | 0.04 | 0.05 | 0.07 |
| hsa-miR-199a-3p | 0.04 | 0.03 | 0.03 | 0.08 | 0.06 |
| hsa-miR-199b-3p | 0.04 | 0.03 | 0.03 | 0.08 | 0.06 |
| hsa-miR-532-5p | 0.04 | 0.05 | 0.03 | 0.05 | 0.05 |
| hsa-miR-29c-3p | 0.04 | 0.03 | 0.02 | 0.03 | 0.03 |
| hsa-miR-193b-5p | 0.04 | 0 | 0 | 0 | 0 |
| hsa-miR-148b-3p | 0.03 | 0.04 | 0.03 | 0.12 | 0.07 |
| hsa-miR-127-3p | 0.03 | 0.02 | 0.01 | 0.09 | 0.03 |
| hsa-miR-32-5p | 0.03 | 0.01 | 0.02 | 0.02 | 0.02 |
| hsa-miR-125b-5p | 0.03 | 0.02 | 0.01 | 0 | 0.01 |
| hsa-miR-483-5p | 0.03 | 0 | 0 | 0 | 0 |
| hsa-miR-30c-5p | 0.02 | 0.09 | 0.05 | 0.16 | 0.08 |
| hsa-miR-744-5p | 0.02 | 0.04 | 0.03 | 0.19 | 0.07 |
| hsa-miR-342-3p | 0.02 | 0.12 | 0.03 | 0.06 | 0.06 |
| hsa-miR-486-3p | 0.02 | 0.03 | 0.02 | 0.03 | 0.04 |
| hsa-miR-106b-5p | 0.02 | 0.02 | 0.02 | 0.03 | 0.04 |
| hsa-miR-4732-3p | 0.02 | 0.05 | 0.03 | 0.02 | 0.04 |
| hsa-miR-19b-3p | 0.02 | 0.03 | 0.02 | 0.03 | 0.03 |
| hsa-miR-421 | 0.02 | 0.02 | 0.03 | 0.03 | 0.03 |
| hsa-miR-15b-5p | 0.02 | 0.01 | 0.01 | 0.03 | 0.03 |
| hsa-miR-181b-5p | 0.02 | 0.03 | 0.03 | 0.02 | 0.03 |
| hsa-miR-146b-5p | 0.02 | 0.03 | 0.01 | 0.04 | 0.02 |
| hsa-miR-501-3p | 0.02 | 0.02 | 0.03 | 0.03 | 0.02 |
| hsa-miR-424-3p | 0.02 | 0.01 | 0.02 | 0.02 | 0.02 |
| hsa-miR-589-5p | 0.02 | 0.01 | 0.02 | 0.01 | 0.01 |
| hsa-let-7b-3p | 0.02 | 0.01 | 0.01 | 0 | 0.01 |
| hsa-miR-194-5p | 0.02 | 0.03 | 0 | 0 | 0 |
| hsa-miR-885-5p | 0.02 | 0.02 | 0.01 | 0 | 0 |
| hsa-miR-152-3p | 0.02 | 0 | 0 | 0 | 0 |
| hsa-miR-320c | 0.02 | 0 | 0 | 0 | 0 |
| hsa-miR-30b-5p | 0.01 | 0.07 | 0.03 | 0.08 | 0.05 |
| hsa-miR-409-3p | 0.01 | 0.03 | 0.02 | 0.14 | 0.04 |
| hsa-miR-17-5p | 0.01 | 0.02 | 0.01 | 0.04 | 0.03 |
| hsa-miR-142-3p | 0.01 | 0.02 | 0.01 | 0.03 | 0.03 |
| hsa-miR-197-3p | 0.01 | 0.03 | 0.02 | 0.02 | 0.03 |
| hsa-miR-215-5p | 0.01 | 0.02 | 0.01 | 0 | 0.03 |
| hsa-let-7e-5p | 0.01 | 0.01 | 0.02 | 0.06 | 0.02 |
| hsa-miR-98-5p | 0.01 | 0.01 | 0.01 | 0.06 | 0.02 |
| hsa-miR-654-3p | 0.01 | 0.01 | 0.01 | 0.05 | 0.02 |
| hsa-miR-1307-3p | 0.01 | 0.02 | 0.01 | 0.04 | 0.02 |
| hsa-miR-181c-5p | 0.01 | 0.01 | 0.01 | 0.04 | 0.02 |
| hsa-miR-410-3p | 0.01 | 0.01 | 0.01 | 0.04 | 0.02 |
| hsa-miR-28-5p | 0.01 | 0.02 | 0.01 | 0.03 | 0.02 |
| hsa-miR-301a-3p | 0.01 | 0.01 | 0.01 | 0.03 | 0.02 |
| hsa-miR-328-3p | 0.01 | 0.02 | 0.02 | 0.02 | 0.02 |
| hsa-miR-30e-3p | 0.01 | 0.02 | 0.01 | 0.02 | 0.02 |
| hsa-miR-19a-3p | 0.01 | 0.01 | 0.01 | 0.02 | 0.02 |
| hsa-miR-20a-5p | 0.01 | 0.01 | 0.01 | 0.02 | 0.02 |
| hsa-miR-181a-2-3p | 0.01 | 0.02 | 0.01 | 0.02 | 0.01 |
| hsa-miR-339-5p | 0.01 | 0.01 | 0.01 | 0.02 | 0.01 |
| hsa-miR-339-3p | 0.01 | 0.01 | 0.01 | 0.02 | 0.01 |
| hsa-miR-769-5p | 0.01 | 0.01 | 0.01 | 0.02 | 0.01 |
| hsa-miR-381-3p | 0.01 | 0 | 0 | 0.02 | 0.01 |
| hsa-let-7c-5p | 0.01 | 0.04 | 0.01 | 0.01 | 0.01 |
| hsa-miR-148a-5p | 0.01 | 0.02 | 0.01 | 0.01 | 0.01 |
| hsa-miR-342-5p | 0.01 | 0.02 | 0.01 | 0.01 | 0.01 |
| hsa-miR-361-3p | 0.01 | 0.02 | 0.01 | 0.01 | 0.01 |
| hsa-miR-155-5p | 0.01 | 0.01 | 0.01 | 0.01 | 0.01 |
| hsa-miR-15b-3p | 0.01 | 0.01 | 0.01 | 0.01 | 0.01 |
| hsa-miR-223-5p | 0.01 | 0.01 | 0.01 | 0.01 | 0.01 |
| hsa-miR-500a-3p | 0.01 | 0.01 | 0.01 | 0.01 | 0.01 |
| hsa-miR-450b-5p | 0.01 | 0 | 0.01 | 0.01 | 0.01 |
| hsa-miR-136-3p | 0.01 | 0 | 0 | 0.01 | 0.01 |
| hsa-miR-151b | 0.01 | 0 | 0 | 0.01 | 0.01 |
| hsa-miR-183-5p | 0.01 | 0 | 0 | 0.01 | 0.01 |
| hsa-miR-24-3p | 0.01 | 0 | 0 | 0.01 | 0.01 |
| hsa-miR-324-3p | 0.01 | 0.01 | 0.01 | 0 | 0.01 |
| hsa-miR-3605-3p | 0.01 | 0.01 | 0.01 | 0 | 0.01 |
| hsa-let-7i-3p | 0.01 | 0 | 0.01 | 0.01 | 0 |
| hsa-miR-1468-5p | 0.01 | 0.01 | 0 | 0 | 0 |
| hsa-miR-146b-3p | 0.01 | 0.01 | 0 | 0 | 0 |
| hsa-miR-193b-3p | 0.01 | 0.01 | 0 | 0 | 0 |
| hsa-miR-30a-3p | 0.01 | 0.01 | 0 | 0 | 0 |
| hsa-miR-483-3p | 0.01 | 0.01 | 0 | 0 | 0 |
| hsa-miR-125b-1-3p | 0.01 | 0 | 0 | 0 | 0 |
| hsa-miR-22-5p | 0.01 | 0 | 0 | 0 | 0 |
| hsa-miR-33b-5p | 0.01 | 0 | 0 | 0 | 0 |
| hsa-miR-378c | 0.01 | 0 | 0 | 0 | 0 |
| hsa-miR-4792 | 0.01 | 0 | 0 | 0 | 0 |
| hsa-miR-497-5p | 0.01 | 0 | 0 | 0 | 0 |
| hsa-miR-502-3p | 0.01 | 0 | 0 | 0 | 0 |
| hsa-miR-505-3p | 0.01 | 0 | 0 | 0 | 0 |
| hsa-miR-5096 | 0.01 | 0 | 0 | 0 | 0 |
| hsa-miR-8061 | 0.01 | 0 | 0 | 0 | 0 |
| hsa-miR-874-3p | 0.01 | 0 | 0 | 0 | 0 |
| hsa-miR-4433b-5p | 0 | 0.03 | 0.01 | 0.02 | 0.05 |
| hsa-miR-1260b | 0 | 0.01 | 0.01 | 0.01 | 0.02 |
| hsa-miR-141-3p | 0 | 0.01 | 0 | 0 | 0.02 |
| hsa-miR-224-5p | 0 | 0 | 0 | 0.02 | 0.01 |
| hsa-miR-411-5p | 0 | 0 | 0 | 0.02 | 0.01 |
| hsa-miR-1304-3p | 0 | 0.01 | 0.01 | 0.01 | 0.01 |
| hsa-miR-181a-3p | 0 | 0.01 | 0.01 | 0.01 | 0.01 |
| hsa-miR-26b-3p | 0 | 0.01 | 0.01 | 0.01 | 0.01 |
| hsa-miR-574-3p | 0 | 0.01 | 0.01 | 0.01 | 0.01 |
| hsa-miR-625-3p | 0 | 0.01 | 0.01 | 0.01 | 0.01 |
| hsa-miR-877-5p | 0 | 0.01 | 0.01 | 0.01 | 0.01 |
| hsa-miR-130b-5p | 0 | 0.01 | 0 | 0.01 | 0.01 |
| hsa-miR-671-3p | 0 | 0.01 | 0 | 0.01 | 0.01 |
| hsa-miR-1273h-3p | 0 | 0 | 0 | 0.01 | 0.01 |
| hsa-miR-199a-5p | 0 | 0 | 0 | 0.01 | 0.01 |
| hsa-miR-326 | 0 | 0 | 0 | 0.01 | 0.01 |
| hsa-miR-4446-3p | 0 | 0 | 0 | 0.01 | 0.01 |
| hsa-miR-93-3p | 0 | 0 | 0 | 0.01 | 0.01 |
| hsa-miR-941 | 0 | 0 | 0 | 0.01 | 0.01 |
| hsa-miR-942-5p | 0 | 0 | 0 | 0.01 | 0.01 |
| hsa-miR-1260a | 0 | 0.01 | 0.01 | 0 | 0.01 |
| hsa-miR-532-3p | 0 | 0.01 | 0.01 | 0 | 0.01 |
| hsa-miR-150-3p | 0 | 0.01 | 0 | 0 | 0.01 |
| hsa-miR-18a-3p | 0 | 0.01 | 0 | 0 | 0.01 |
| hsa-miR-96-5p | 0 | 0 | 0.01 | 0.01 | 0 |
| hsa-miR-1301-3p | 0 | 0 | 0 | 0.01 | 0 |
| hsa-miR-133a-3p | 0 | 0 | 0 | 0.01 | 0 |
| hsa-miR-134-5p | 0 | 0 | 0 | 0.01 | 0 |
| hsa-miR-148b-5p | 0 | 0 | 0 | 0.01 | 0 |
| hsa-miR-181c-3p | 0 | 0 | 0 | 0.01 | 0 |
| hsa-miR-181d-5p | 0 | 0 | 0 | 0.01 | 0 |
| hsa-miR-221-5p | 0 | 0 | 0 | 0.01 | 0 |
| hsa-miR-301b-3p | 0 | 0 | 0 | 0.01 | 0 |
| hsa-miR-323b-3p | 0 | 0 | 0 | 0.01 | 0 |
| hsa-miR-330-3p | 0 | 0 | 0 | 0.01 | 0 |
| hsa-miR-335-3p | 0 | 0 | 0 | 0.01 | 0 |
| hsa-miR-340-3p | 0 | 0 | 0 | 0.01 | 0 |
| hsa-miR-374a-5p | 0 | 0 | 0 | 0.01 | 0 |
| hsa-miR-374b-5p | 0 | 0 | 0 | 0.01 | 0 |
| hsa-miR-425-3p | 0 | 0 | 0 | 0.01 | 0 |
| hsa-miR-431-5p | 0 | 0 | 0 | 0.01 | 0 |
| hsa-miR-432-5p | 0 | 0 | 0 | 0.01 | 0 |
| hsa-miR-454-5p | 0 | 0 | 0 | 0.01 | 0 |
| hsa-miR-454-3p | 0 | 0 | 0 | 0.01 | 0 |
| hsa-miR-485-5p | 0 | 0 | 0 | 0.01 | 0 |
| hsa-miR-485-3p | 0 | 0 | 0 | 0.01 | 0 |
| hsa-miR-487b-3p | 0 | 0 | 0 | 0.01 | 0 |
| hsa-miR-5010-5p | 0 | 0 | 0 | 0.01 | 0 |
| hsa-miR-628-3p | 0 | 0 | 0 | 0.01 | 0 |
| hsa-miR-6852-5p | 0 | 0 | 0 | 0.01 | 0 |
| hsa-miR-889-3p | 0 | 0 | 0 | 0.01 | 0 |
| hsa-miR-122-3p | 0 | 0.02 | 0 | 0 | 0 |
| hsa-miR-204-5p | 0 | 0.01 | 0 | 0 | 0 |
| hsa-miR-424-5p | 0 | 0 | 0.01 | 0 | 0 |
| hsa-miR-4508 | 0 | 0 | 0.01 | 0 | 0 |
| hsa-let-7a-3p | 0 | 0 | 0 | 0 | 0 |
| hsa-let-7c-3p | 0 | 0 | 0 | 0 | 0 |
| hsa-let-7e-3p | 0 | 0 | 0 | 0 | 0 |
| hsa-let-7f-1-3p | 0 | 0 | 0 | 0 | 0 |
| hsa-let-7f-2-3p | 0 | 0 | 0 | 0 | 0 |
| hsa-let-7g-3p | 0 | 0 | 0 | 0 | 0 |
| hsa-miR-101-5p | 0 | 0 | 0 | 0 | 0 |
| hsa-miR-103a-2-5p | 0 | 0 | 0 | 0 | 0 |
| hsa-miR-106a-3p | 0 | 0 | 0 | 0 | 0 |
| hsa-miR-106a-5p | 0 | 0 | 0 | 0 | 0 |
| hsa-miR-10a-3p | 0 | 0 | 0 | 0 | 0 |
| hsa-miR-10b-3p | 0 | 0 | 0 | 0 | 0 |
| hsa-miR-1180-3p | 0 | 0 | 0 | 0 | 0 |
| hsa-miR-1185-5p | 0 | 0 | 0 | 0 | 0 |
| hsa-miR-1185-1-3p | 0 | 0 | 0 | 0 | 0 |
| hsa-miR-1185-2-3p | 0 | 0 | 0 | 0 | 0 |
| hsa-miR-1197 | 0 | 0 | 0 | 0 | 0 |
| hsa-miR-1224-5p | 0 | 0 | 0 | 0 | 0 |
| hsa-miR-1226-3p | 0 | 0 | 0 | 0 | 0 |
| hsa-miR-1228-3p | 0 | 0 | 0 | 0 | 0 |
| hsa-miR-1229-3p | 0 | 0 | 0 | 0 | 0 |
| hsa-miR-1234-3p | 0 | 0 | 0 | 0 | 0 |
| hsa-miR-1247-5p | 0 | 0 | 0 | 0 | 0 |
| hsa-miR-1249-3p | 0 | 0 | 0 | 0 | 0 |
| hsa-miR-1254 | 0 | 0 | 0 | 0 | 0 |
| hsa-miR-1255a | 0 | 0 | 0 | 0 | 0 |
| hsa-miR-1255b-5p | 0 | 0 | 0 | 0 | 0 |
| hsa-miR-1256 | 0 | 0 | 0 | 0 | 0 |
| hsa-miR-125a-3p | 0 | 0 | 0 | 0 | 0 |
| hsa-miR-1262 | 0 | 0 | 0 | 0 | 0 |
| hsa-miR-1266-5p | 0 | 0 | 0 | 0 | 0 |
| hsa-miR-127-5p | 0 | 0 | 0 | 0 | 0 |
| hsa-miR-1270 | 0 | 0 | 0 | 0 | 0 |
| hsa-miR-1271-5p | 0 | 0 | 0 | 0 | 0 |
| hsa-miR-1273d | 0 | 0 | 0 | 0 | 0 |
| hsa-miR-1273f | 0 | 0 | 0 | 0 | 0 |
| hsa-miR-1273g-3p | 0 | 0 | 0 | 0 | 0 |
| hsa-miR-1273h-5p | 0 | 0 | 0 | 0 | 0 |
| hsa-miR-1275 | 0 | 0 | 0 | 0 | 0 |
| hsa-miR-1276 | 0 | 0 | 0 | 0 | 0 |
| hsa-miR-1277-5p | 0 | 0 | 0 | 0 | 0 |
| hsa-miR-1277-3p | 0 | 0 | 0 | 0 | 0 |
| hsa-miR-1278 | 0 | 0 | 0 | 0 | 0 |
| hsa-miR-128-1-5p | 0 | 0 | 0 | 0 | 0 |
| hsa-miR-1283 | 0 | 0 | 0 | 0 | 0 |
| hsa-miR-1284 | 0 | 0 | 0 | 0 | 0 |
| hsa-miR-1285-3p | 0 | 0 | 0 | 0 | 0 |
| hsa-miR-1285-5p | 0 | 0 | 0 | 0 | 0 |
| hsa-miR-1287-5p | 0 | 0 | 0 | 0 | 0 |
| hsa-miR-1290 | 0 | 0 | 0 | 0 | 0 |
| hsa-miR-1291 | 0 | 0 | 0 | 0 | 0 |
| hsa-miR-1292-5p | 0 | 0 | 0 | 0 | 0 |
| hsa-miR-1294 | 0 | 0 | 0 | 0 | 0 |
| hsa-miR-1295b-5p | 0 | 0 | 0 | 0 | 0 |
| hsa-miR-1296-5p | 0 | 0 | 0 | 0 | 0 |
| hsa-miR-1299 | 0 | 0 | 0 | 0 | 0 |
| hsa-miR-1303 | 0 | 0 | 0 | 0 | 0 |
| hsa-miR-1304-5p | 0 | 0 | 0 | 0 | 0 |
| hsa-miR-1306-5p | 0 | 0 | 0 | 0 | 0 |
| hsa-miR-1306-3p | 0 | 0 | 0 | 0 | 0 |
| hsa-miR-132-3p | 0 | 0 | 0 | 0 | 0 |
| hsa-miR-132-5p | 0 | 0 | 0 | 0 | 0 |
| hsa-miR-1323 | 0 | 0 | 0 | 0 | 0 |
| hsa-miR-133b | 0 | 0 | 0 | 0 | 0 |
| hsa-miR-134-3p | 0 | 0 | 0 | 0 | 0 |
| hsa-miR-1343-3p | 0 | 0 | 0 | 0 | 0 |
| hsa-miR-135a-3p | 0 | 0 | 0 | 0 | 0 |
| hsa-miR-136-5p | 0 | 0 | 0 | 0 | 0 |
| hsa-miR-138-5p | 0 | 0 | 0 | 0 | 0 |
| hsa-miR-139-3p | 0 | 0 | 0 | 0 | 0 |
| hsa-miR-139-5p | 0 | 0 | 0 | 0 | 0 |
| hsa-miR-140-5p | 0 | 0 | 0 | 0 | 0 |
| hsa-miR-143-5p | 0 | 0 | 0 | 0 | 0 |
| hsa-miR-145-5p | 0 | 0 | 0 | 0 | 0 |
| hsa-miR-145-3p | 0 | 0 | 0 | 0 | 0 |
| hsa-miR-146a-3p | 0 | 0 | 0 | 0 | 0 |
| hsa-miR-149-5p | 0 | 0 | 0 | 0 | 0 |
| hsa-miR-152-5p | 0 | 0 | 0 | 0 | 0 |
| hsa-miR-153-3p | 0 | 0 | 0 | 0 | 0 |
| hsa-miR-1537-3p | 0 | 0 | 0 | 0 | 0 |
| hsa-miR-1538 | 0 | 0 | 0 | 0 | 0 |
| hsa-miR-154-5p | 0 | 0 | 0 | 0 | 0 |
| hsa-miR-15a-3p | 0 | 0 | 0 | 0 | 0 |
| hsa-miR-16-1-3p | 0 | 0 | 0 | 0 | 0 |
| hsa-miR-17-3p | 0 | 0 | 0 | 0 | 0 |
| hsa-miR-181b-3p | 0 | 0 | 0 | 0 | 0 |
| hsa-miR-181b-2-3p | 0 | 0 | 0 | 0 | 0 |
| hsa-miR-181d-3p | 0 | 0 | 0 | 0 | 0 |
| hsa-miR-183-3p | 0 | 0 | 0 | 0 | 0 |
| hsa-miR-184 | 0 | 0 | 0 | 0 | 0 |
| hsa-miR-185-5p | 0 | 0 | 0 | 0 | 0 |
| hsa-miR-185-3p | 0 | 0 | 0 | 0 | 0 |
| hsa-miR-186-3p | 0 | 0 | 0 | 0 | 0 |
| hsa-miR-187-3p | 0 | 0 | 0 | 0 | 0 |
| hsa-miR-188-5p | 0 | 0 | 0 | 0 | 0 |
| hsa-miR-18a-5p | 0 | 0 | 0 | 0 | 0 |
| hsa-miR-18b-5p | 0 | 0 | 0 | 0 | 0 |
| hsa-miR-18b-3p | 0 | 0 | 0 | 0 | 0 |
| hsa-miR-1908-5p | 0 | 0 | 0 | 0 | 0 |
| hsa-miR-1908-3p | 0 | 0 | 0 | 0 | 0 |
| hsa-miR-190a-5p | 0 | 0 | 0 | 0 | 0 |
| hsa-miR-190b | 0 | 0 | 0 | 0 | 0 |
| hsa-miR-191-3p | 0 | 0 | 0 | 0 | 0 |
| hsa-miR-1910-5p | 0 | 0 | 0 | 0 | 0 |
| hsa-miR-1914-5p | 0 | 0 | 0 | 0 | 0 |
| hsa-miR-193a-3p | 0 | 0 | 0 | 0 | 0 |
| hsa-miR-193a-5p | 0 | 0 | 0 | 0 | 0 |
| hsa-miR-194-3p | 0 | 0 | 0 | 0 | 0 |
| hsa-miR-195-3p | 0 | 0 | 0 | 0 | 0 |
| hsa-miR-195-5p | 0 | 0 | 0 | 0 | 0 |
| hsa-miR-196b-5p | 0 | 0 | 0 | 0 | 0 |
| hsa-miR-196b-3p | 0 | 0 | 0 | 0 | 0 |
| hsa-miR-197-5p | 0 | 0 | 0 | 0 | 0 |
| hsa-miR-1976 | 0 | 0 | 0 | 0 | 0 |
| hsa-miR-199b-5p | 0 | 0 | 0 | 0 | 0 |
| hsa-miR-19a-5p | 0 | 0 | 0 | 0 | 0 |
| hsa-miR-200a-3p | 0 | 0 | 0 | 0 | 0 |
| hsa-miR-200b-3p | 0 | 0 | 0 | 0 | 0 |
| hsa-miR-200c-3p | 0 | 0 | 0 | 0 | 0 |
| hsa-miR-202-3p | 0 | 0 | 0 | 0 | 0 |
| hsa-miR-203a-3p | 0 | 0 | 0 | 0 | 0 |
| hsa-miR-204-3p | 0 | 0 | 0 | 0 | 0 |
| hsa-miR-205-5p | 0 | 0 | 0 | 0 | 0 |
| hsa-miR-208b-3p | 0 | 0 | 0 | 0 | 0 |
| hsa-miR-20a-3p | 0 | 0 | 0 | 0 | 0 |
| hsa-miR-20b-5p | 0 | 0 | 0 | 0 | 0 |
| hsa-miR-20b-3p | 0 | 0 | 0 | 0 | 0 |
| hsa-miR-210-5p | 0 | 0 | 0 | 0 | 0 |
| hsa-miR-2110 | 0 | 0 | 0 | 0 | 0 |
| hsa-miR-2114-3p | 0 | 0 | 0 | 0 | 0 |
| hsa-miR-2115-3p | 0 | 0 | 0 | 0 | 0 |
| hsa-miR-2115-5p | 0 | 0 | 0 | 0 | 0 |
| hsa-miR-2116-3p | 0 | 0 | 0 | 0 | 0 |
| hsa-miR-212-3p | 0 | 0 | 0 | 0 | 0 |
| hsa-miR-212-5p | 0 | 0 | 0 | 0 | 0 |
| hsa-miR-214-5p | 0 | 0 | 0 | 0 | 0 |
| hsa-miR-214-3p | 0 | 0 | 0 | 0 | 0 |
| hsa-miR-219a-1-3p | 0 | 0 | 0 | 0 | 0 |
| hsa-miR-219b-5p | 0 | 0 | 0 | 0 | 0 |
| hsa-miR-222-5p | 0 | 0 | 0 | 0 | 0 |
| hsa-miR-224-3p | 0 | 0 | 0 | 0 | 0 |
| hsa-miR-2276-3p | 0 | 0 | 0 | 0 | 0 |
| hsa-miR-2277-3p | 0 | 0 | 0 | 0 | 0 |
| hsa-miR-2277-5p | 0 | 0 | 0 | 0 | 0 |
| hsa-miR-2355-5p | 0 | 0 | 0 | 0 | 0 |
| hsa-miR-2355-3p | 0 | 0 | 0 | 0 | 0 |
| hsa-miR-23a-5p | 0 | 0 | 0 | 0 | 0 |
| hsa-miR-23b-5p | 0 | 0 | 0 | 0 | 0 |
| hsa-miR-24-2-5p | 0 | 0 | 0 | 0 | 0 |
| hsa-miR-2467-5p | 0 | 0 | 0 | 0 | 0 |
| hsa-miR-25-5p | 0 | 0 | 0 | 0 | 0 |
| hsa-miR-26a-1-3p | 0 | 0 | 0 | 0 | 0 |
| hsa-miR-26a-2-3p | 0 | 0 | 0 | 0 | 0 |
| hsa-miR-27a-5p | 0 | 0 | 0 | 0 | 0 |
| hsa-miR-27b-5p | 0 | 0 | 0 | 0 | 0 |
| hsa-miR-296-5p | 0 | 0 | 0 | 0 | 0 |
| hsa-miR-299-3p | 0 | 0 | 0 | 0 | 0 |
| hsa-miR-29a-5p | 0 | 0 | 0 | 0 | 0 |
| hsa-miR-29b-3p | 0 | 0 | 0 | 0 | 0 |
| hsa-miR-29b-2-5p | 0 | 0 | 0 | 0 | 0 |
| hsa-miR-29c-5p | 0 | 0 | 0 | 0 | 0 |
| hsa-miR-301a-5p | 0 | 0 | 0 | 0 | 0 |
| hsa-miR-3064-5p | 0 | 0 | 0 | 0 | 0 |
| hsa-miR-3065-3p | 0 | 0 | 0 | 0 | 0 |
| hsa-miR-3065-5p | 0 | 0 | 0 | 0 | 0 |
| hsa-miR-3074-5p | 0 | 0 | 0 | 0 | 0 |
| hsa-miR-3074-3p | 0 | 0 | 0 | 0 | 0 |
| hsa-miR-30b-3p | 0 | 0 | 0 | 0 | 0 |
| hsa-miR-30c-1-3p | 0 | 0 | 0 | 0 | 0 |
| hsa-miR-30c-2-3p | 0 | 0 | 0 | 0 | 0 |
| hsa-miR-30d-3p | 0 | 0 | 0 | 0 | 0 |
| hsa-miR-31-5p | 0 | 0 | 0 | 0 | 0 |
| hsa-miR-3120-3p | 0 | 0 | 0 | 0 | 0 |
| hsa-miR-3120-5p | 0 | 0 | 0 | 0 | 0 |
| hsa-miR-3121-3p | 0 | 0 | 0 | 0 | 0 |
| hsa-miR-3122 | 0 | 0 | 0 | 0 | 0 |
| hsa-miR-3124-5p | 0 | 0 | 0 | 0 | 0 |
| hsa-miR-3127-5p | 0 | 0 | 0 | 0 | 0 |
| hsa-miR-3127-3p | 0 | 0 | 0 | 0 | 0 |
| hsa-miR-3130-5p | 0 | 0 | 0 | 0 | 0 |
| hsa-miR-3135a | 0 | 0 | 0 | 0 | 0 |
| hsa-miR-3136-5p | 0 | 0 | 0 | 0 | 0 |
| hsa-miR-3138 | 0 | 0 | 0 | 0 | 0 |
| hsa-miR-3143 | 0 | 0 | 0 | 0 | 0 |
| hsa-miR-3150a-5p | 0 | 0 | 0 | 0 | 0 |
| hsa-miR-3150b-3p | 0 | 0 | 0 | 0 | 0 |
| hsa-miR-3157-3p | 0 | 0 | 0 | 0 | 0 |
| hsa-miR-3157-5p | 0 | 0 | 0 | 0 | 0 |
| hsa-miR-3158-3p | 0 | 0 | 0 | 0 | 0 |
| hsa-miR-3159 | 0 | 0 | 0 | 0 | 0 |
| hsa-miR-3161 | 0 | 0 | 0 | 0 | 0 |
| hsa-miR-3163 | 0 | 0 | 0 | 0 | 0 |
| hsa-miR-3166 | 0 | 0 | 0 | 0 | 0 |
| hsa-miR-3173-5p | 0 | 0 | 0 | 0 | 0 |
| hsa-miR-3173-3p | 0 | 0 | 0 | 0 | 0 |
| hsa-miR-3174 | 0 | 0 | 0 | 0 | 0 |
| hsa-miR-3176 | 0 | 0 | 0 | 0 | 0 |
| hsa-miR-3177-3p | 0 | 0 | 0 | 0 | 0 |
| hsa-miR-3186-3p | 0 | 0 | 0 | 0 | 0 |
| hsa-miR-3187-3p | 0 | 0 | 0 | 0 | 0 |
| hsa-miR-3188 | 0 | 0 | 0 | 0 | 0 |
| hsa-miR-3190-3p | 0 | 0 | 0 | 0 | 0 |
| hsa-miR-3191-3p | 0 | 0 | 0 | 0 | 0 |
| hsa-miR-3196 | 0 | 0 | 0 | 0 | 0 |
| hsa-miR-32-3p | 0 | 0 | 0 | 0 | 0 |
| hsa-miR-3200-5p | 0 | 0 | 0 | 0 | 0 |
| hsa-miR-3200-3p | 0 | 0 | 0 | 0 | 0 |
| hsa-miR-320d | 0 | 0 | 0 | 0 | 0 |
| hsa-miR-323a-3p | 0 | 0 | 0 | 0 | 0 |
| hsa-miR-324-5p | 0 | 0 | 0 | 0 | 0 |
| hsa-miR-329-5p | 0 | 0 | 0 | 0 | 0 |
| hsa-miR-329-3p | 0 | 0 | 0 | 0 | 0 |
| hsa-miR-330-5p | 0 | 0 | 0 | 0 | 0 |
| hsa-miR-331-3p | 0 | 0 | 0 | 0 | 0 |
| hsa-miR-331-5p | 0 | 0 | 0 | 0 | 0 |
| hsa-miR-337-5p | 0 | 0 | 0 | 0 | 0 |
| hsa-miR-337-3p | 0 | 0 | 0 | 0 | 0 |
| hsa-miR-338-5p | 0 | 0 | 0 | 0 | 0 |
| hsa-miR-338-3p | 0 | 0 | 0 | 0 | 0 |
| hsa-miR-33a-5p | 0 | 0 | 0 | 0 | 0 |
| hsa-miR-33a-3p | 0 | 0 | 0 | 0 | 0 |
| hsa-miR-34a-5p | 0 | 0 | 0 | 0 | 0 |
| hsa-miR-34c-5p | 0 | 0 | 0 | 0 | 0 |
| hsa-miR-3591-5p | 0 | 0 | 0 | 0 | 0 |
| hsa-miR-3605-5p | 0 | 0 | 0 | 0 | 0 |
| hsa-miR-3607-3p | 0 | 0 | 0 | 0 | 0 |
| hsa-miR-3613-5p | 0 | 0 | 0 | 0 | 0 |
| hsa-miR-3613-3p | 0 | 0 | 0 | 0 | 0 |
| hsa-miR-3614-5p | 0 | 0 | 0 | 0 | 0 |
| hsa-miR-3617-3p | 0 | 0 | 0 | 0 | 0 |
| hsa-miR-3617-5p | 0 | 0 | 0 | 0 | 0 |
| hsa-miR-362-3p | 0 | 0 | 0 | 0 | 0 |
| hsa-miR-362-5p | 0 | 0 | 0 | 0 | 0 |
| hsa-miR-3653-3p | 0 | 0 | 0 | 0 | 0 |
| hsa-miR-3656 | 0 | 0 | 0 | 0 | 0 |
| hsa-miR-365a-3p | 0 | 0 | 0 | 0 | 0 |
| hsa-miR-365b-3p | 0 | 0 | 0 | 0 | 0 |
| hsa-miR-365b-5p | 0 | 0 | 0 | 0 | 0 |
| hsa-miR-3661 | 0 | 0 | 0 | 0 | 0 |
| hsa-miR-3675-5p | 0 | 0 | 0 | 0 | 0 |
| hsa-miR-3677-3p | 0 | 0 | 0 | 0 | 0 |
| hsa-miR-3684 | 0 | 0 | 0 | 0 | 0 |
| hsa-miR-3688-3p | 0 | 0 | 0 | 0 | 0 |
| hsa-miR-369-5p | 0 | 0 | 0 | 0 | 0 |
| hsa-miR-369-3p | 0 | 0 | 0 | 0 | 0 |
| hsa-miR-3690 | 0 | 0 | 0 | 0 | 0 |
| hsa-miR-3691-5p | 0 | 0 | 0 | 0 | 0 |
| hsa-miR-370-3p | 0 | 0 | 0 | 0 | 0 |
| hsa-miR-370-5p | 0 | 0 | 0 | 0 | 0 |
| hsa-miR-371b-3p | 0 | 0 | 0 | 0 | 0 |
| hsa-miR-371b-5p | 0 | 0 | 0 | 0 | 0 |
| hsa-miR-374a-3p | 0 | 0 | 0 | 0 | 0 |
| hsa-miR-374b-3p | 0 | 0 | 0 | 0 | 0 |
| hsa-miR-376a-5p | 0 | 0 | 0 | 0 | 0 |
| hsa-miR-376a-3p | 0 | 0 | 0 | 0 | 0 |
| hsa-miR-376b-3p | 0 | 0 | 0 | 0 | 0 |
| hsa-miR-376c-3p | 0 | 0 | 0 | 0 | 0 |
| hsa-miR-377-5p | 0 | 0 | 0 | 0 | 0 |
| hsa-miR-377-3p | 0 | 0 | 0 | 0 | 0 |
| hsa-miR-378a-5p | 0 | 0 | 0 | 0 | 0 |
| hsa-miR-378d | 0 | 0 | 0 | 0 | 0 |
| hsa-miR-378f | 0 | 0 | 0 | 0 | 0 |
| hsa-miR-378g | 0 | 0 | 0 | 0 | 0 |
| hsa-miR-378i | 0 | 0 | 0 | 0 | 0 |
| hsa-miR-379-5p | 0 | 0 | 0 | 0 | 0 |
| hsa-miR-379-3p | 0 | 0 | 0 | 0 | 0 |
| hsa-miR-380-3p | 0 | 0 | 0 | 0 | 0 |
| hsa-miR-382-3p | 0 | 0 | 0 | 0 | 0 |
| hsa-miR-382-5p | 0 | 0 | 0 | 0 | 0 |
| hsa-miR-3909 | 0 | 0 | 0 | 0 | 0 |
| hsa-miR-3912-3p | 0 | 0 | 0 | 0 | 0 |
| hsa-miR-3913-5p | 0 | 0 | 0 | 0 | 0 |
| hsa-miR-3916 | 0 | 0 | 0 | 0 | 0 |
| hsa-miR-3918 | 0 | 0 | 0 | 0 | 0 |
| hsa-miR-3920 | 0 | 0 | 0 | 0 | 0 |
| hsa-miR-3928-3p | 0 | 0 | 0 | 0 | 0 |
| hsa-miR-3938 | 0 | 0 | 0 | 0 | 0 |
| hsa-miR-3939 | 0 | 0 | 0 | 0 | 0 |
| hsa-miR-3940-3p | 0 | 0 | 0 | 0 | 0 |
| hsa-miR-3942-5p | 0 | 0 | 0 | 0 | 0 |
| hsa-miR-3960 | 0 | 0 | 0 | 0 | 0 |
| hsa-miR-409-5p | 0 | 0 | 0 | 0 | 0 |
| hsa-miR-411-3p | 0 | 0 | 0 | 0 | 0 |
| hsa-miR-412-5p | 0 | 0 | 0 | 0 | 0 |
| hsa-miR-4286 | 0 | 0 | 0 | 0 | 0 |
| hsa-miR-429 | 0 | 0 | 0 | 0 | 0 |
| hsa-miR-431-3p | 0 | 0 | 0 | 0 | 0 |
| hsa-miR-432-3p | 0 | 0 | 0 | 0 | 0 |
| hsa-miR-4326 | 0 | 0 | 0 | 0 | 0 |
| hsa-miR-433-3p | 0 | 0 | 0 | 0 | 0 |
| hsa-miR-433-5p | 0 | 0 | 0 | 0 | 0 |
| hsa-miR-4422 | 0 | 0 | 0 | 0 | 0 |
| hsa-miR-4433b-3p | 0 | 0 | 0 | 0 | 0 |
| hsa-miR-4435 | 0 | 0 | 0 | 0 | 0 |
| hsa-miR-4440 | 0 | 0 | 0 | 0 | 0 |
| hsa-miR-4446-5p | 0 | 0 | 0 | 0 | 0 |
| hsa-miR-4448 | 0 | 0 | 0 | 0 | 0 |
| hsa-miR-4449 | 0 | 0 | 0 | 0 | 0 |
| hsa-miR-4454 | 0 | 0 | 0 | 0 | 0 |
| hsa-miR-4466 | 0 | 0 | 0 | 0 | 0 |
| hsa-miR-4467 | 0 | 0 | 0 | 0 | 0 |
| hsa-miR-4482-3p | 0 | 0 | 0 | 0 | 0 |
| hsa-miR-4485-3p | 0 | 0 | 0 | 0 | 0 |
| hsa-miR-4492 | 0 | 0 | 0 | 0 | 0 |
| hsa-miR-449c-5p | 0 | 0 | 0 | 0 | 0 |
| hsa-miR-4507 | 0 | 0 | 0 | 0 | 0 |
| hsa-miR-450a-5p | 0 | 0 | 0 | 0 | 0 |
| hsa-miR-450a-2-3p | 0 | 0 | 0 | 0 | 0 |
| hsa-miR-4511 | 0 | 0 | 0 | 0 | 0 |
| hsa-miR-4516 | 0 | 0 | 0 | 0 | 0 |
| hsa-miR-452-5p | 0 | 0 | 0 | 0 | 0 |
| hsa-miR-4523 | 0 | 0 | 0 | 0 | 0 |
| hsa-miR-4526 | 0 | 0 | 0 | 0 | 0 |
| hsa-miR-4532 | 0 | 0 | 0 | 0 | 0 |
| hsa-miR-455-3p | 0 | 0 | 0 | 0 | 0 |
| hsa-miR-455-5p | 0 | 0 | 0 | 0 | 0 |
| hsa-miR-4638-3p | 0 | 0 | 0 | 0 | 0 |
| hsa-miR-4645-3p | 0 | 0 | 0 | 0 | 0 |
| hsa-miR-4646-5p | 0 | 0 | 0 | 0 | 0 |
| hsa-miR-4647 | 0 | 0 | 0 | 0 | 0 |
| hsa-miR-4657 | 0 | 0 | 0 | 0 | 0 |
| hsa-miR-4659b-3p | 0 | 0 | 0 | 0 | 0 |
| hsa-miR-4660 | 0 | 0 | 0 | 0 | 0 |
| hsa-miR-4661-5p | 0 | 0 | 0 | 0 | 0 |
| hsa-miR-4662a-5p | 0 | 0 | 0 | 0 | 0 |
| hsa-miR-4664-3p | 0 | 0 | 0 | 0 | 0 |
| hsa-miR-4665-5p | 0 | 0 | 0 | 0 | 0 |
| hsa-miR-4672 | 0 | 0 | 0 | 0 | 0 |
| hsa-miR-4676-3p | 0 | 0 | 0 | 0 | 0 |
| hsa-miR-4677-3p | 0 | 0 | 0 | 0 | 0 |
| hsa-miR-4685-3p | 0 | 0 | 0 | 0 | 0 |
| hsa-miR-4687-5p | 0 | 0 | 0 | 0 | 0 |
| hsa-miR-4688 | 0 | 0 | 0 | 0 | 0 |
| hsa-miR-4701-5p | 0 | 0 | 0 | 0 | 0 |
| hsa-miR-4707-3p | 0 | 0 | 0 | 0 | 0 |
| hsa-miR-4714-5p | 0 | 0 | 0 | 0 | 0 |
| hsa-miR-4714-3p | 0 | 0 | 0 | 0 | 0 |
| hsa-miR-4732-5p | 0 | 0 | 0 | 0 | 0 |
| hsa-miR-4738-3p | 0 | 0 | 0 | 0 | 0 |
| hsa-miR-4741 | 0 | 0 | 0 | 0 | 0 |
| hsa-miR-4742-5p | 0 | 0 | 0 | 0 | 0 |
| hsa-miR-4742-3p | 0 | 0 | 0 | 0 | 0 |
| hsa-miR-4745-5p | 0 | 0 | 0 | 0 | 0 |
| hsa-miR-4746-5p | 0 | 0 | 0 | 0 | 0 |
| hsa-miR-4748 | 0 | 0 | 0 | 0 | 0 |
| hsa-miR-4751 | 0 | 0 | 0 | 0 | 0 |
| hsa-miR-4753-5p | 0 | 0 | 0 | 0 | 0 |
| hsa-miR-4755-5p | 0 | 0 | 0 | 0 | 0 |
| hsa-miR-4755-3p | 0 | 0 | 0 | 0 | 0 |
| hsa-miR-4757-3p | 0 | 0 | 0 | 0 | 0 |
| hsa-miR-4762-5p | 0 | 0 | 0 | 0 | 0 |
| hsa-miR-4767 | 0 | 0 | 0 | 0 | 0 |
| hsa-miR-4772-3p | 0 | 0 | 0 | 0 | 0 |
| hsa-miR-4772-5p | 0 | 0 | 0 | 0 | 0 |
| hsa-miR-4773 | 0 | 0 | 0 | 0 | 0 |
| hsa-miR-4775 | 0 | 0 | 0 | 0 | 0 |
| hsa-miR-4777-3p | 0 | 0 | 0 | 0 | 0 |
| hsa-miR-4781-3p | 0 | 0 | 0 | 0 | 0 |
| hsa-miR-4785 | 0 | 0 | 0 | 0 | 0 |
| hsa-miR-4786-5p | 0 | 0 | 0 | 0 | 0 |
| hsa-miR-4796-3p | 0 | 0 | 0 | 0 | 0 |
| hsa-miR-4797-3p | 0 | 0 | 0 | 0 | 0 |
| hsa-miR-487a-3p | 0 | 0 | 0 | 0 | 0 |
| hsa-miR-487a-5p | 0 | 0 | 0 | 0 | 0 |
| hsa-miR-487b-5p | 0 | 0 | 0 | 0 | 0 |
| hsa-miR-490-3p | 0 | 0 | 0 | 0 | 0 |
| hsa-miR-491-5p | 0 | 0 | 0 | 0 | 0 |
| hsa-miR-493-3p | 0 | 0 | 0 | 0 | 0 |
| hsa-miR-493-5p | 0 | 0 | 0 | 0 | 0 |
| hsa-miR-494-5p | 0 | 0 | 0 | 0 | 0 |
| hsa-miR-494-3p | 0 | 0 | 0 | 0 | 0 |
| hsa-miR-495-3p | 0 | 0 | 0 | 0 | 0 |
| hsa-miR-496 | 0 | 0 | 0 | 0 | 0 |
| hsa-miR-499a-5p | 0 | 0 | 0 | 0 | 0 |
| hsa-miR-5001-3p | 0 | 0 | 0 | 0 | 0 |
| hsa-miR-5006-3p | 0 | 0 | 0 | 0 | 0 |
| hsa-miR-500a-5p | 0 | 0 | 0 | 0 | 0 |
| hsa-miR-500b-5p | 0 | 0 | 0 | 0 | 0 |
| hsa-miR-501-5p | 0 | 0 | 0 | 0 | 0 |
| hsa-miR-5010-3p | 0 | 0 | 0 | 0 | 0 |
| hsa-miR-502-5p | 0 | 0 | 0 | 0 | 0 |
| hsa-miR-503-5p | 0 | 0 | 0 | 0 | 0 |
| hsa-miR-504-5p | 0 | 0 | 0 | 0 | 0 |
| hsa-miR-505-5p | 0 | 0 | 0 | 0 | 0 |
| hsa-miR-509-3p | 0 | 0 | 0 | 0 | 0 |
| hsa-miR-5094 | 0 | 0 | 0 | 0 | 0 |
| hsa-miR-5100 | 0 | 0 | 0 | 0 | 0 |
| hsa-miR-511-5p | 0 | 0 | 0 | 0 | 0 |
| hsa-miR-512-3p | 0 | 0 | 0 | 0 | 0 |
| hsa-miR-516b-5p | 0 | 0 | 0 | 0 | 0 |
| hsa-miR-5187-5p | 0 | 0 | 0 | 0 | 0 |
| hsa-miR-5187-3p | 0 | 0 | 0 | 0 | 0 |
| hsa-miR-5189-3p | 0 | 0 | 0 | 0 | 0 |
| hsa-miR-5189-5p | 0 | 0 | 0 | 0 | 0 |
| hsa-miR-5193 | 0 | 0 | 0 | 0 | 0 |
| hsa-miR-520a-3p | 0 | 0 | 0 | 0 | 0 |
| hsa-miR-539-3p | 0 | 0 | 0 | 0 | 0 |
| hsa-miR-539-5p | 0 | 0 | 0 | 0 | 0 |
| hsa-miR-542-3p | 0 | 0 | 0 | 0 | 0 |
| hsa-miR-542-5p | 0 | 0 | 0 | 0 | 0 |
| hsa-miR-543 | 0 | 0 | 0 | 0 | 0 |
| hsa-miR-545-5p | 0 | 0 | 0 | 0 | 0 |
| hsa-miR-548a-3p | 0 | 0 | 0 | 0 | 0 |
| hsa-miR-548a-5p | 0 | 0 | 0 | 0 | 0 |
| hsa-miR-548ab | 0 | 0 | 0 | 0 | 0 |
| hsa-miR-548ac | 0 | 0 | 0 | 0 | 0 |
| hsa-miR-548ad-5p | 0 | 0 | 0 | 0 | 0 |
| hsa-miR-548ae-5p | 0 | 0 | 0 | 0 | 0 |
| hsa-miR-548ah-3p | 0 | 0 | 0 | 0 | 0 |
| hsa-miR-548aj-5p | 0 | 0 | 0 | 0 | 0 |
| hsa-miR-548al | 0 | 0 | 0 | 0 | 0 |
| hsa-miR-548am-5p | 0 | 0 | 0 | 0 | 0 |
| hsa-miR-548ap-5p | 0 | 0 | 0 | 0 | 0 |
| hsa-miR-548aq-3p | 0 | 0 | 0 | 0 | 0 |
| hsa-miR-548ar-5p | 0 | 0 | 0 | 0 | 0 |
| hsa-miR-548at-5p | 0 | 0 | 0 | 0 | 0 |
| hsa-miR-548au-5p | 0 | 0 | 0 | 0 | 0 |
| hsa-miR-548ax | 0 | 0 | 0 | 0 | 0 |
| hsa-miR-548ay-5p | 0 | 0 | 0 | 0 | 0 |
| hsa-miR-548ay-3p | 0 | 0 | 0 | 0 | 0 |
| hsa-miR-548az-5p | 0 | 0 | 0 | 0 | 0 |
| hsa-miR-548b-5p | 0 | 0 | 0 | 0 | 0 |
| hsa-miR-548c-5p | 0 | 0 | 0 | 0 | 0 |
| hsa-miR-548c-3p | 0 | 0 | 0 | 0 | 0 |
| hsa-miR-548d-3p | 0 | 0 | 0 | 0 | 0 |
| hsa-miR-548d-5p | 0 | 0 | 0 | 0 | 0 |
| hsa-miR-548e-3p | 0 | 0 | 0 | 0 | 0 |
| hsa-miR-548e-5p | 0 | 0 | 0 | 0 | 0 |
| hsa-miR-548f-5p | 0 | 0 | 0 | 0 | 0 |
| hsa-miR-548g-5p | 0 | 0 | 0 | 0 | 0 |
| hsa-miR-548h-5p | 0 | 0 | 0 | 0 | 0 |
| hsa-miR-548h-3p | 0 | 0 | 0 | 0 | 0 |
| hsa-miR-548j-5p | 0 | 0 | 0 | 0 | 0 |
| hsa-miR-548j-3p | 0 | 0 | 0 | 0 | 0 |
| hsa-miR-548k | 0 | 0 | 0 | 0 | 0 |
| hsa-miR-548l | 0 | 0 | 0 | 0 | 0 |
| hsa-miR-548n | 0 | 0 | 0 | 0 | 0 |
| hsa-miR-548o-3p | 0 | 0 | 0 | 0 | 0 |
| hsa-miR-548o-5p | 0 | 0 | 0 | 0 | 0 |
| hsa-miR-548p | 0 | 0 | 0 | 0 | 0 |
| hsa-miR-548q | 0 | 0 | 0 | 0 | 0 |
| hsa-miR-548u | 0 | 0 | 0 | 0 | 0 |
| hsa-miR-548w | 0 | 0 | 0 | 0 | 0 |
| hsa-miR-548x-5p | 0 | 0 | 0 | 0 | 0 |
| hsa-miR-548z | 0 | 0 | 0 | 0 | 0 |
| hsa-miR-550a-5p | 0 | 0 | 0 | 0 | 0 |
| hsa-miR-550a-3-5p | 0 | 0 | 0 | 0 | 0 |
| hsa-miR-550a-3p | 0 | 0 | 0 | 0 | 0 |
| hsa-miR-550b-3p | 0 | 0 | 0 | 0 | 0 |
| hsa-miR-551a | 0 | 0 | 0 | 0 | 0 |
| hsa-miR-551b-3p | 0 | 0 | 0 | 0 | 0 |
| hsa-miR-556-3p | 0 | 0 | 0 | 0 | 0 |
| hsa-miR-556-5p | 0 | 0 | 0 | 0 | 0 |
| hsa-miR-5581-3p | 0 | 0 | 0 | 0 | 0 |
| hsa-miR-5587-5p | 0 | 0 | 0 | 0 | 0 |
| hsa-miR-5588-5p | 0 | 0 | 0 | 0 | 0 |
| hsa-miR-561-5p | 0 | 0 | 0 | 0 | 0 |
| hsa-miR-5683 | 0 | 0 | 0 | 0 | 0 |
| hsa-miR-5695 | 0 | 0 | 0 | 0 | 0 |
| hsa-miR-570-3p | 0 | 0 | 0 | 0 | 0 |
| hsa-miR-5706 | 0 | 0 | 0 | 0 | 0 |
| hsa-miR-574-5p | 0 | 0 | 0 | 0 | 0 |
| hsa-miR-576-3p | 0 | 0 | 0 | 0 | 0 |
| hsa-miR-576-5p | 0 | 0 | 0 | 0 | 0 |
| hsa-miR-577 | 0 | 0 | 0 | 0 | 0 |
| hsa-miR-579-5p | 0 | 0 | 0 | 0 | 0 |
| hsa-miR-579-3p | 0 | 0 | 0 | 0 | 0 |
| hsa-miR-580-3p | 0 | 0 | 0 | 0 | 0 |
| hsa-miR-582-5p | 0 | 0 | 0 | 0 | 0 |
| hsa-miR-582-3p | 0 | 0 | 0 | 0 | 0 |
| hsa-miR-584-3p | 0 | 0 | 0 | 0 | 0 |
| hsa-miR-589-3p | 0 | 0 | 0 | 0 | 0 |
| hsa-miR-590-5p | 0 | 0 | 0 | 0 | 0 |
| hsa-miR-590-3p | 0 | 0 | 0 | 0 | 0 |
| hsa-miR-592 | 0 | 0 | 0 | 0 | 0 |
| hsa-miR-597-3p | 0 | 0 | 0 | 0 | 0 |
| hsa-miR-598-3p | 0 | 0 | 0 | 0 | 0 |
| hsa-miR-605-5p | 0 | 0 | 0 | 0 | 0 |
| hsa-miR-605-3p | 0 | 0 | 0 | 0 | 0 |
| hsa-miR-610 | 0 | 0 | 0 | 0 | 0 |
| hsa-miR-615-3p | 0 | 0 | 0 | 0 | 0 |
| hsa-miR-616-5p | 0 | 0 | 0 | 0 | 0 |
| hsa-miR-616-3p | 0 | 0 | 0 | 0 | 0 |
| hsa-miR-618 | 0 | 0 | 0 | 0 | 0 |
| hsa-miR-619-5p | 0 | 0 | 0 | 0 | 0 |
| hsa-miR-624-3p | 0 | 0 | 0 | 0 | 0 |
| hsa-miR-624-5p | 0 | 0 | 0 | 0 | 0 |
| hsa-miR-625-5p | 0 | 0 | 0 | 0 | 0 |
| hsa-miR-627-3p | 0 | 0 | 0 | 0 | 0 |
| hsa-miR-627-5p | 0 | 0 | 0 | 0 | 0 |
| hsa-miR-628-5p | 0 | 0 | 0 | 0 | 0 |
| hsa-miR-629-3p | 0 | 0 | 0 | 0 | 0 |
| hsa-miR-629-5p | 0 | 0 | 0 | 0 | 0 |
| hsa-miR-636 | 0 | 0 | 0 | 0 | 0 |
| hsa-miR-641 | 0 | 0 | 0 | 0 | 0 |
| hsa-miR-642a-3p | 0 | 0 | 0 | 0 | 0 |
| hsa-miR-643 | 0 | 0 | 0 | 0 | 0 |
| hsa-miR-6500-3p | 0 | 0 | 0 | 0 | 0 |
| hsa-miR-6501-5p | 0 | 0 | 0 | 0 | 0 |
| hsa-miR-6502-5p | 0 | 0 | 0 | 0 | 0 |
| hsa-miR-6503-5p | 0 | 0 | 0 | 0 | 0 |
| hsa-miR-6503-3p | 0 | 0 | 0 | 0 | 0 |
| hsa-miR-6509-5p | 0 | 0 | 0 | 0 | 0 |
| hsa-miR-651-5p | 0 | 0 | 0 | 0 | 0 |
| hsa-miR-6511a-3p | 0 | 0 | 0 | 0 | 0 |
| hsa-miR-6511b-5p | 0 | 0 | 0 | 0 | 0 |
| hsa-miR-6511b-3p | 0 | 0 | 0 | 0 | 0 |
| hsa-miR-6513-3p | 0 | 0 | 0 | 0 | 0 |
| hsa-miR-6513-5p | 0 | 0 | 0 | 0 | 0 |
| hsa-miR-6514-5p | 0 | 0 | 0 | 0 | 0 |
| hsa-miR-6515-5p | 0 | 0 | 0 | 0 | 0 |
| hsa-miR-6516-5p | 0 | 0 | 0 | 0 | 0 |
| hsa-miR-6516-3p | 0 | 0 | 0 | 0 | 0 |
| hsa-miR-652-5p | 0 | 0 | 0 | 0 | 0 |
| hsa-miR-654-5p | 0 | 0 | 0 | 0 | 0 |
| hsa-miR-655-3p | 0 | 0 | 0 | 0 | 0 |
| hsa-miR-656-3p | 0 | 0 | 0 | 0 | 0 |
| hsa-miR-659-5p | 0 | 0 | 0 | 0 | 0 |
| hsa-miR-660-3p | 0 | 0 | 0 | 0 | 0 |
| hsa-miR-664a-3p | 0 | 0 | 0 | 0 | 0 |
| hsa-miR-664a-5p | 0 | 0 | 0 | 0 | 0 |
| hsa-miR-664b-5p | 0 | 0 | 0 | 0 | 0 |
| hsa-miR-664b-3p | 0 | 0 | 0 | 0 | 0 |
| hsa-miR-665 | 0 | 0 | 0 | 0 | 0 |
| hsa-miR-668-3p | 0 | 0 | 0 | 0 | 0 |
| hsa-miR-671-5p | 0 | 0 | 0 | 0 | 0 |
| hsa-miR-6715a-3p | 0 | 0 | 0 | 0 | 0 |
| hsa-miR-6716-3p | 0 | 0 | 0 | 0 | 0 |
| hsa-miR-6721-5p | 0 | 0 | 0 | 0 | 0 |
| hsa-miR-6730-3p | 0 | 0 | 0 | 0 | 0 |
| hsa-miR-6734-5p | 0 | 0 | 0 | 0 | 0 |
| hsa-miR-6735-5p | 0 | 0 | 0 | 0 | 0 |
| hsa-miR-6735-3p | 0 | 0 | 0 | 0 | 0 |
| hsa-miR-6740-5p | 0 | 0 | 0 | 0 | 0 |
| hsa-miR-6741-3p | 0 | 0 | 0 | 0 | 0 |
| hsa-miR-6747-3p | 0 | 0 | 0 | 0 | 0 |
| hsa-miR-675-3p | 0 | 0 | 0 | 0 | 0 |
| hsa-miR-6750-3p | 0 | 0 | 0 | 0 | 0 |
| hsa-miR-6754-3p | 0 | 0 | 0 | 0 | 0 |
| hsa-miR-6755-5p | 0 | 0 | 0 | 0 | 0 |
| hsa-miR-6762-3p | 0 | 0 | 0 | 0 | 0 |
| hsa-miR-6764-3p | 0 | 0 | 0 | 0 | 0 |
| hsa-miR-6764-5p | 0 | 0 | 0 | 0 | 0 |
| hsa-miR-6767-5p | 0 | 0 | 0 | 0 | 0 |
| hsa-miR-6770-3p | 0 | 0 | 0 | 0 | 0 |
| hsa-miR-6772-3p | 0 | 0 | 0 | 0 | 0 |
| hsa-miR-6775-3p | 0 | 0 | 0 | 0 | 0 |
| hsa-miR-6777-3p | 0 | 0 | 0 | 0 | 0 |
| hsa-miR-6780a-5p | 0 | 0 | 0 | 0 | 0 |
| hsa-miR-6783-5p | 0 | 0 | 0 | 0 | 0 |
| hsa-miR-6786-3p | 0 | 0 | 0 | 0 | 0 |
| hsa-miR-6802-3p | 0 | 0 | 0 | 0 | 0 |
| hsa-miR-6802-5p | 0 | 0 | 0 | 0 | 0 |
| hsa-miR-6803-3p | 0 | 0 | 0 | 0 | 0 |
| hsa-miR-6804-5p | 0 | 0 | 0 | 0 | 0 |
| hsa-miR-6809-5p | 0 | 0 | 0 | 0 | 0 |
| hsa-miR-6810-5p | 0 | 0 | 0 | 0 | 0 |
| hsa-miR-6813-5p | 0 | 0 | 0 | 0 | 0 |
| hsa-miR-6815-5p | 0 | 0 | 0 | 0 | 0 |
| hsa-miR-6816-3p | 0 | 0 | 0 | 0 | 0 |
| hsa-miR-6817-3p | 0 | 0 | 0 | 0 | 0 |
| hsa-miR-6818-5p | 0 | 0 | 0 | 0 | 0 |
| hsa-miR-6819-3p | 0 | 0 | 0 | 0 | 0 |
| hsa-miR-6820-5p | 0 | 0 | 0 | 0 | 0 |
| hsa-miR-6832-5p | 0 | 0 | 0 | 0 | 0 |
| hsa-miR-6837-3p | 0 | 0 | 0 | 0 | 0 |
| hsa-miR-6838-5p | 0 | 0 | 0 | 0 | 0 |
| hsa-miR-6840-5p | 0 | 0 | 0 | 0 | 0 |
| hsa-miR-6842-5p | 0 | 0 | 0 | 0 | 0 |
| hsa-miR-6842-3p | 0 | 0 | 0 | 0 | 0 |
| hsa-miR-6847-5p | 0 | 0 | 0 | 0 | 0 |
| hsa-miR-6850-5p | 0 | 0 | 0 | 0 | 0 |
| hsa-miR-6855-3p | 0 | 0 | 0 | 0 | 0 |
| hsa-miR-6859-5p | 0 | 0 | 0 | 0 | 0 |
| hsa-miR-6861-5p | 0 | 0 | 0 | 0 | 0 |
| hsa-miR-6862-5p | 0 | 0 | 0 | 0 | 0 |
| hsa-miR-6866-5p | 0 | 0 | 0 | 0 | 0 |
| hsa-miR-6868-3p | 0 | 0 | 0 | 0 | 0 |
| hsa-miR-6875-5p | 0 | 0 | 0 | 0 | 0 |
| hsa-miR-6876-5p | 0 | 0 | 0 | 0 | 0 |
| hsa-miR-6881-3p | 0 | 0 | 0 | 0 | 0 |
| hsa-miR-6882-5p | 0 | 0 | 0 | 0 | 0 |
| hsa-miR-6884-5p | 0 | 0 | 0 | 0 | 0 |
| hsa-miR-6891-5p | 0 | 0 | 0 | 0 | 0 |
| hsa-miR-6892-5p | 0 | 0 | 0 | 0 | 0 |
| hsa-miR-6894-5p | 0 | 0 | 0 | 0 | 0 |
| hsa-miR-7-1-3p | 0 | 0 | 0 | 0 | 0 |
| hsa-miR-744-3p | 0 | 0 | 0 | 0 | 0 |
| hsa-miR-758-3p | 0 | 0 | 0 | 0 | 0 |
| hsa-miR-760 | 0 | 0 | 0 | 0 | 0 |
| hsa-miR-7641 | 0 | 0 | 0 | 0 | 0 |
| hsa-miR-766-3p | 0 | 0 | 0 | 0 | 0 |
| hsa-miR-766-5p | 0 | 0 | 0 | 0 | 0 |
| hsa-miR-769-3p | 0 | 0 | 0 | 0 | 0 |
| hsa-miR-7704 | 0 | 0 | 0 | 0 | 0 |
| hsa-miR-7705 | 0 | 0 | 0 | 0 | 0 |
| hsa-miR-7706 | 0 | 0 | 0 | 0 | 0 |
| hsa-miR-7848-3p | 0 | 0 | 0 | 0 | 0 |
| hsa-miR-7849-3p | 0 | 0 | 0 | 0 | 0 |
| hsa-miR-7850-5p | 0 | 0 | 0 | 0 | 0 |
| hsa-miR-7854-3p | 0 | 0 | 0 | 0 | 0 |
| hsa-miR-7855-5p | 0 | 0 | 0 | 0 | 0 |
| hsa-miR-7856-5p | 0 | 0 | 0 | 0 | 0 |
| hsa-miR-7976 | 0 | 0 | 0 | 0 | 0 |
| hsa-miR-7977 | 0 | 0 | 0 | 0 | 0 |
| hsa-miR-873-5p | 0 | 0 | 0 | 0 | 0 |
| hsa-miR-873-3p | 0 | 0 | 0 | 0 | 0 |
| hsa-miR-874-5p | 0 | 0 | 0 | 0 | 0 |
| hsa-miR-885-3p | 0 | 0 | 0 | 0 | 0 |
| hsa-miR-887-3p | 0 | 0 | 0 | 0 | 0 |
| hsa-miR-889-5p | 0 | 0 | 0 | 0 | 0 |
| hsa-miR-9-5p | 0 | 0 | 0 | 0 | 0 |
| hsa-miR-92a-1-5p | 0 | 0 | 0 | 0 | 0 |
| hsa-miR-92b-5p | 0 | 0 | 0 | 0 | 0 |
| hsa-miR-933 | 0 | 0 | 0 | 0 | 0 |
| hsa-miR-937-3p | 0 | 0 | 0 | 0 | 0 |
| hsa-miR-939-3p | 0 | 0 | 0 | 0 | 0 |
| hsa-miR-939-5p | 0 | 0 | 0 | 0 | 0 |
| hsa-miR-940 | 0 | 0 | 0 | 0 | 0 |
| hsa-miR-942-3p | 0 | 0 | 0 | 0 | 0 |
| hsa-miR-943 | 0 | 0 | 0 | 0 | 0 |
| hsa-miR-95-3p | 0 | 0 | 0 | 0 | 0 |
| hsa-miR-98-3p | 0 | 0 | 0 | 0 | 0 |
| hsa-miR-99a-3p | 0 | 0 | 0 | 0 | 0 |
| hsa-miR-99b-3p | 0 | 0 | 0 | 0 | 0 |
