# Supplementary material for: Serum microRNA signatures as "liquid biopsies" for interrogating hepatotoxic mechanisms and liver pathogenesis in human
Source: PLoS One. 2017 May 17;12(5):e0177928. doi: 10.1371/journal.pone.0177928 (PMC5435338; doi:10.1371/journal.pone.0177928)
Supplement: S4 Table — List of significantly altered miRNAs detected for all liver impairments and T2DM. (DOCX) [file pone.0177928.s004.docx]

| miRNA | Condition | Regulation | Log2 Fold Change | FDR |
| --- | --- | --- | --- | --- |
| hsa-let-7a-3p | APAP | Up | 0.87 | 8.77E-03 |
| hsa-let-7a-5p | T2DM | Up | 1.27 | 1.41E-04 |
| hsa-let-7b-3p | APAP | Up | 1.40 | 8.86E-06 |
| hsa-let-7c-5p | APAP | Up | 1.54 | 9.97E-03 |
| hsa-let-7c-5p | HBV | Up | 3.07 | 5.31E-07 |
| hsa-let-7d-5p | APAP | Down | -0.85 | 2.55E-03 |
| hsa-let-7d-5p | T2DM | Up | 1.07 | 5.96E-04 |
| hsa-let-7g-3p | APAP | Up | 2.01 | 2.07E-04 |
| hsa-let-7i-3p | APAP | Up | 1.25 | 7.73E-04 |
| hsa-miR-100-5p | APAP | Up | 3.64 | 1.15E-20 |
| hsa-miR-100-5p | HBV | Up | 1.57 | 3.66E-03 |
| hsa-miR-101-3p | T2DM | Down | -0.94 | 6.04E-03 |
| hsa-miR-103a-3p | APAP | Up | 2.34 | 1.20E-10 |
| hsa-miR-107 | APAP | Up | 2.53 | 1.24E-10 |
| hsa-miR-10b-5p | T2DM | Down | -2.55 | 3.79E-12 |
| hsa-miR-122-3p | HBV | Up | 6.18 | 1.51E-15 |
| hsa-miR-122-5p | APAP | Up | 5.18 | 5.32E-18 |
| hsa-miR-122-5p | HBV | Up | 4.82 | 4.99E-13 |
| hsa-miR-1226-3p | T2DM | Down | -2.63 | 4.67E-04 |
| hsa-miR-1228-3p | T2DM | Down | -3.00 | 2.26E-03 |
| hsa-miR-1246 | APAP | Up | 3.17 | 3.10E-06 |
| hsa-miR-1247-5p | APAP | Up | 2.49 | 2.62E-03 |
| hsa-miR-1249-3p | APAP | Down | -4.01 | 5.61E-07 |
| hsa-miR-1249-3p | T2DM | Down | -4.10 | 1.30E-09 |
| hsa-miR-125b-1-3p | APAP | Up | 4.42 | 1.15E-15 |
| hsa-miR-125b-2-3p | APAP | Up | 4.93 | 4.69E-23 |
| hsa-miR-125b-2-3p | HBV | Up | 2.28 | 3.72E-04 |
| hsa-miR-125b-5p | APAP | Up | 2.39 | 1.36E-07 |
| hsa-miR-125b-5p | HBV | Up | 1.68 | 4.70E-03 |
| hsa-miR-1260a | APAP | Down | -1.53 | 1.78E-03 |
| hsa-miR-1260a | T2DM | Down | -1.93 | 1.69E-04 |
| hsa-miR-1260b | APAP | Down | -1.97 | 5.63E-05 |
| hsa-miR-1260b | T2DM | Down | -1.97 | 3.04E-04 |
| hsa-miR-1273g-3p | T2DM | Down | -2.60 | 4.76E-03 |
| hsa-miR-1273h-3p | APAP | Down | -1.60 | 4.78E-04 |
| hsa-miR-127-3p | T2DM | Up | 1.51 | 4.19E-03 |
| hsa-miR-1285-5p | APAP | Up | 2.64 | 7.96E-05 |
| hsa-miR-1290 | APAP | Up | 5.01 | 1.74E-06 |
| hsa-miR-1291 | APAP | Up | 2.59 | 9.27E-03 |
| hsa-miR-1296-5p | T2DM | Up | 1.62 | 1.35E-03 |
| hsa-miR-1301-3p | T2DM | Up | 1.38 | 1.81E-03 |
| hsa-miR-1303 | APAP | Up | 2.37 | 9.11E-04 |
| hsa-miR-1304-3p | APAP | Down | -1.29 | 5.73E-05 |
| hsa-miR-1306-5p | APAP | Down | -2.30 | 1.08E-04 |
| hsa-miR-1306-5p | T2DM | Down | -1.82 | 7.74E-04 |
| hsa-miR-1307-3p | T2DM | Up | 0.90 | 8.40E-03 |
| hsa-miR-1307-5p | APAP | Up | 1.38 | 4.26E-06 |
| hsa-miR-130a-3p | APAP | Up | 2.90 | 3.45E-23 |
| hsa-miR-130b-3p | APAP | Up | 2.98 | 2.47E-20 |
| hsa-miR-130b-5p | APAP | Down | -1.29 | 1.39E-03 |
| hsa-miR-1343-3p | APAP | Down | -2.61 | 7.31E-03 |
| hsa-miR-141-3p | APAP | Down | -1.56 | 8.77E-03 |
| hsa-miR-141-3p | LC | Down | -1.94 | 3.54E-03 |
| hsa-miR-141-3p | T2DM | Down | -2.21 | 4.47E-04 |
| hsa-miR-142-3p | APAP | Down | -0.84 | 2.14E-03 |
| hsa-miR-142-5p | APAP | Down | -0.84 | 6.48E-03 |
| hsa-miR-144-3p | T2DM | Down | -1.31 | 2.86E-03 |
| hsa-miR-1468-5p | APAP | Up | 1.25 | 1.50E-03 |
| hsa-miR-1468-5p | HBV | Up | 1.77 | 1.09E-05 |
| hsa-miR-148a-3p | APAP | Up | 2.20 | 6.33E-10 |
| hsa-miR-148a-5p | HBV | Up | 1.97 | 2.49E-05 |
| hsa-miR-150-5p | APAP | Down | -1.73 | 3.01E-03 |
| hsa-miR-151a-3p | T2DM | Up | 0.79 | 6.24E-03 |
| hsa-miR-151a-5p | T2DM | Up | 1.03 | 4.53E-03 |
| hsa-miR-151b | APAP | Up | 1.11 | 1.76E-04 |
| hsa-miR-152-3p | APAP | Up | 2.08 | 8.61E-07 |
| hsa-miR-17-3p | LC | Down | -1.17 | 2.44E-03 |
| hsa-miR-181c-3p | T2DM | Up | 1.24 | 4.19E-03 |
| hsa-miR-188-5p | APAP | Up | 1.41 | 8.74E-03 |
| hsa-miR-18a-3p | APAP | Down | -1.22 | 5.98E-03 |
| hsa-miR-1908-3p | T2DM | Down | -1.96 | 1.75E-03 |
| hsa-miR-190a-5p | HBV | Down | -2.54 | 3.29E-03 |
| hsa-miR-191-3p | APAP | Down | -1.14 | 6.90E-03 |
| hsa-miR-191-5p | APAP | Down | -1.18 | 1.39E-03 |
| hsa-miR-192-5p | APAP | Up | 2.26 | 9.08E-09 |
| hsa-miR-192-5p | HBV | Up | 2.70 | 1.06E-09 |
| hsa-miR-193a-5p | APAP | Up | 3.92 | 6.78E-08 |
| hsa-miR-193b-3p | APAP | Up | 2.55 | 4.03E-06 |
| hsa-miR-193b-3p | HBV | Up | 3.18 | 6.36E-08 |
| hsa-miR-193b-5p | APAP | Up | 5.98 | 3.36E-27 |
| hsa-miR-193b-5p | LC | Up | 2.08 | 5.78E-03 |
| hsa-miR-194-3p | HBV | Up | 4.70 | 3.83E-05 |
| hsa-miR-194-5p | APAP | Up | 2.56 | 1.20E-10 |
| hsa-miR-194-5p | HBV | Up | 3.25 | 2.46E-14 |
| hsa-miR-200a-3p | T2DM | Down | -2.59 | 5.58E-04 |
| hsa-miR-203a-3p | T2DM | Down | -2.46 | 2.15E-03 |
| hsa-miR-204-5p | HBV | Up | 1.65 | 9.16E-03 |
| hsa-miR-204-5p | T2DM | Down | -1.96 | 6.07E-04 |
| hsa-miR-205-5p | T2DM | Down | -2.48 | 1.41E-04 |
| hsa-miR-210-3p | APAP | Up | 1.87 | 1.92E-08 |
| hsa-miR-2116-3p | T2DM | Down | -2.44 | 1.89E-03 |
| hsa-miR-21-3p | APAP | Up | 1.93 | 1.92E-08 |
| hsa-miR-214-3p | T2DM | Down | -2.71 | 4.19E-03 |
| hsa-miR-214-5p | T2DM | Down | -2.49 | 8.40E-03 |
| hsa-miR-215-5p | T2DM | Down | -2.94 | 7.47E-07 |
| hsa-miR-21-5p | APAP | Up | 1.48 | 8.37E-07 |
| hsa-miR-221-3p | APAP | Up | 1.24 | 7.53E-06 |
| hsa-miR-221-5p | T2DM | Up | 1.37 | 6.01E-03 |
| hsa-miR-222-3p | APAP | Down | -1.10 | 2.85E-03 |
| hsa-miR-222-3p | T2DM | Up | 1.18 | 4.53E-03 |
| hsa-miR-22-3p | APAP | Up | 1.89 | 2.49E-12 |
| hsa-miR-22-5p | APAP | Up | 2.56 | 2.16E-14 |
| hsa-miR-23a-3p | APAP | Up | 1.47 | 6.29E-07 |
| hsa-miR-23b-3p | APAP | Up | 3.15 | 1.85E-19 |
| hsa-miR-24-3p | APAP | Up | 1.56 | 7.17E-06 |
| hsa-miR-26a-5p | T2DM | Up | 1.12 | 1.68E-03 |
| hsa-miR-27a-5p | T2DM | Down | -2.13 | 4.53E-03 |
| hsa-miR-27b-3p | APAP | Up | 3.22 | 1.50E-18 |
| hsa-miR-28-3p | HBV | Up | 1.20 | 7.20E-04 |
| hsa-miR-29c-3p | APAP | Up | 0.78 | 1.78E-03 |
| hsa-miR-301a-3p | APAP | Down | -1.10 | 3.37E-03 |
| hsa-miR-30a-5p | APAP | Up | 2.62 | 4.31E-13 |
| hsa-miR-30a-5p | T2DM | Down | -1.78 | 1.15E-04 |
| hsa-miR-30b-5p | APAP | Down | -1.14 | 2.14E-03 |
| hsa-miR-30c-5p | APAP | Down | -1.34 | 3.20E-04 |
| hsa-miR-3173-5p | T2DM | Down | -1.80 | 1.44E-03 |
| hsa-miR-320a | APAP | Up | 3.67 | 5.52E-28 |
| hsa-miR-320b | APAP | Up | 5.59 | 4.06E-34 |
| hsa-miR-320b | LC | Up | 1.93 | 1.54E-03 |
| hsa-miR-320c | APAP | Up | 3.50 | 5.45E-22 |
| hsa-miR-320d | APAP | Up | 4.29 | 2.39E-10 |
| hsa-miR-328-3p | APAP | Down | -1.03 | 3.74E-03 |
| hsa-miR-335-3p | T2DM | Up | 1.70 | 3.38E-04 |
| hsa-miR-335-5p | APAP | Up | 1.44 | 1.06E-06 |
| hsa-miR-338-5p | LC | Up | 1.65 | 6.88E-03 |
| hsa-miR-339-5p | T2DM | Up | 1.01 | 3.22E-03 |
| hsa-miR-33b-5p | APAP | Up | 2.19 | 4.48E-07 |
| hsa-miR-33b-5p | T2DM | Down | -1.98 | 1.41E-04 |
| hsa-miR-342-3p | APAP | Down | -1.52 | 2.28E-03 |
| hsa-miR-345-5p | APAP | Up | 1.20 | 2.75E-07 |
| hsa-miR-34a-5p | APAP | Up | 4.20 | 2.15E-07 |
| hsa-miR-3591-5p | APAP | Up | 4.12 | 2.95E-04 |
| hsa-miR-3615 | LC | Up | 1.04 | 3.11E-03 |
| hsa-miR-361-5p | APAP | Up | 2.37 | 7.86E-15 |
| hsa-miR-362-3p | APAP | Up | 1.99 | 1.46E-03 |
| hsa-miR-365a-3p | HBV | Up | 1.77 | 8.32E-03 |
| hsa-miR-365b-3p | HBV | Up | 1.77 | 8.32E-03 |
| hsa-miR-375 | T2DM | Down | -2.37 | 1.41E-04 |
| hsa-miR-377-5p | T2DM | Up | 2.04 | 8.40E-03 |
| hsa-miR-378a-3p | APAP | Up | 2.28 | 1.54E-12 |
| hsa-miR-378a-3p | HBV | Up | 1.89 | 6.42E-07 |
| hsa-miR-378a-5p | APAP | Up | 2.11 | 1.41E-03 |
| hsa-miR-378c | APAP | Up | 2.82 | 1.47E-14 |
| hsa-miR-378d | APAP | Up | 2.79 | 3.66E-04 |
| hsa-miR-378i | APAP | Up | 2.83 | 3.28E-10 |
| hsa-miR-378i | HBV | Up | 2.05 | 9.34E-05 |
| hsa-miR-409-3p | T2DM | Up | 1.74 | 2.26E-03 |
| hsa-miR-421 | LC | Up | 0.63 | 9.56E-03 |
| hsa-miR-423-5p | LC | Up | 1.07 | 3.54E-03 |
| hsa-miR-432-5p | T2DM | Up | 1.95 | 4.09E-04 |
| hsa-miR-4433b-5p | APAP | Down | -3.00 | 9.59E-08 |
| hsa-miR-4448 | APAP | Up | 2.19 | 8.26E-03 |
| hsa-miR-4482-3p | T2DM | Down | -2.54 | 5.27E-03 |
| hsa-miR-4492 | APAP | Up | 2.62 | 9.97E-03 |
| hsa-miR-4507 | APAP | Up | 3.56 | 1.78E-03 |
| hsa-miR-4516 | APAP | Up | 2.35 | 5.56E-04 |
| hsa-miR-4516 | T2DM | Down | -3.94 | 7.05E-07 |
| hsa-miR-452-5p | APAP | Up | 2.51 | 1.28E-03 |
| hsa-miR-4532 | LC | Up | 3.30 | 1.54E-03 |
| hsa-miR-455-3p | HBV | Up | 3.87 | 3.08E-03 |
| hsa-miR-455-5p | APAP | Up | 2.86 | 4.36E-03 |
| hsa-miR-455-5p | HBV | Up | 4.81 | 5.50E-07 |
| hsa-miR-4792 | APAP | Up | 2.71 | 9.70E-06 |
| hsa-miR-483-3p | T2DM | Down | -4.84 | 1.45E-12 |
| hsa-miR-483-5p | APAP | Up | 5.21 | 2.66E-18 |
| hsa-miR-483-5p | LC | Up | 2.02 | 9.56E-03 |
| hsa-miR-484 | LC | Up | 1.55 | 7.09E-03 |
| hsa-miR-485-5p | T2DM | Up | 1.76 | 2.29E-03 |
| hsa-miR-487b-3p | T2DM | Up | 1.86 | 1.68E-03 |
| hsa-miR-497-5p | APAP | Up | 2.01 | 8.92E-05 |
| hsa-miR-499a-5p | APAP | Up | 2.46 | 6.66E-04 |
| hsa-miR-505-3p | APAP | Up | 1.20 | 1.06E-03 |
| hsa-miR-5096 | APAP | Up | 2.49 | 8.69E-05 |
| hsa-miR-5096 | LC | Up | 2.05 | 7.09E-03 |
| hsa-miR-5096 | T2DM | Down | -3.62 | 4.08E-07 |
| hsa-miR-5193 | APAP | Down | -2.58 | 8.77E-03 |
| hsa-miR-532-3p | LC | Up | 1.36 | 8.81E-03 |
| hsa-miR-5588-5p | APAP | Up | 4.99 | 2.60E-11 |
| hsa-miR-574-3p | APAP | Down | -1.15 | 6.62E-03 |
| hsa-miR-574-5p | APAP | Up | 3.79 | 2.43E-11 |
| hsa-miR-589-5p | APAP | Up | 0.96 | 1.45E-03 |
| hsa-miR-589-5p | LC | Up | 1.11 | 1.54E-03 |
| hsa-miR-592 | APAP | Up | 2.90 | 9.06E-03 |
| hsa-miR-6087 | APAP | Up | 4.54 | 6.86E-17 |
| hsa-miR-6087 | T2DM | Down | -2.61 | 1.41E-04 |
| hsa-miR-625-3p | APAP | Down | -1.86 | 7.59E-06 |
| hsa-miR-627-5p | APAP | Up | 1.46 | 3.62E-03 |
| hsa-miR-628-3p | T2DM | Up | 0.86 | 8.40E-03 |
| hsa-miR-629-5p | LC | Up | 1.55 | 8.81E-03 |
| hsa-miR-6511a-3p | T2DM | Down | -1.52 | 4.19E-03 |
| hsa-miR-6511b-3p | T2DM | Down | -2.13 | 1.10E-04 |
| hsa-miR-651-5p | APAP | Up | 1.74 | 2.00E-03 |
| hsa-miR-660-5p | APAP | Up | 1.29 | 1.66E-04 |
| hsa-miR-6741-3p | APAP | Down | -2.23 | 8.85E-04 |
| hsa-miR-6741-3p | T2DM | Down | -1.89 | 1.16E-03 |
| hsa-miR-6803-3p | T2DM | Down | -1.43 | 8.40E-03 |
| hsa-miR-6815-5p | APAP | Up | 3.86 | 3.59E-05 |
| hsa-miR-6852-5p | APAP | Down | -1.41 | 7.86E-03 |
| hsa-miR-6881-3p | HBV | Down | -3.35 | 7.77E-03 |
| hsa-miR-744-5p | APAP | Down | -1.48 | 1.57E-04 |
| hsa-miR-744-5p | T2DM | Up | 1.31 | 4.19E-03 |
| hsa-miR-766-3p | APAP | Down | -1.81 | 4.36E-03 |
| hsa-miR-7704 | APAP | Up | 2.19 | 5.78E-03 |
| hsa-miR-7704 | T2DM | Down | -2.69 | 2.15E-03 |
| hsa-miR-7977 | APAP | Down | -1.81 | 8.77E-03 |
| hsa-miR-8061 | T2DM | Down | -3.08 | 1.80E-03 |
| hsa-miR-885-5p | APAP | Up | 3.18 | 2.14E-07 |
| hsa-miR-885-5p | HBV | Up | 3.04 | 1.83E-05 |
| hsa-miR-885-5p | LC | Up | 1.98 | 9.56E-03 |
| hsa-miR-885-5p | T2DM | Down | -2.40 | 1.27E-03 |
| hsa-miR-92b-3p | T2DM | Down | -1.49 | 1.91E-04 |
| hsa-miR-93-5p | APAP | Down | -1.01 | 7.69E-04 |
| hsa-miR-93-5p | LC | Down | -0.91 | 9.10E-03 |
| hsa-miR-939-3p | T2DM | Down | -2.98 | 2.29E-03 |
| hsa-miR-98-5p | T2DM | Up | 1.43 | 4.09E-04 |
| hsa-miR-99a-3p | APAP | Up | 3.80 | 4.13E-06 |
| hsa-miR-99a-3p | HBV | Up | 2.73 | 8.58E-03 |
| hsa-miR-99a-5p | APAP | Up | 3.95 | 1.49E-22 |
| hsa-miR-99a-5p | HBV | Up | 1.57 | 5.43E-03 |
