# Supplementary material for: Serum microRNA signatures as "liquid biopsies" for interrogating hepatotoxic mechanisms and liver pathogenesis in human
Source: PLoS One. 2017 May 17;12(5):e0177928. doi: 10.1371/journal.pone.0177928 (PMC5435338; doi:10.1371/journal.pone.0177928)
Supplement: S5 Table — The list consist of miRNAs that show differences in the predominantly observed miRNA the conditions. (DOCX) [file pone.0177928.s005.docx]

| miRNA | APAP | HBV | HC | LC | T2DM |
| --- | --- | --- | --- | --- | --- |
| hsa-miR-548e-5p | 3' trimmed | 3' trimmed | 3' trimmed | canonical | canonical |
| hsa-miR-3127-5p | 3' trimmed | 3' trimmed | 3' trimmed | 3' trimmed | canonical |
| hsa-miR-98-5p | 3' trimmed | canonical | canonical | canonical | canonical |
| hsa-miR-151a-5p | canonical | canonical | canonical | 3' addition t | canonical |
| hsa-miR-671-5p | 3' addition nt | 3' addition nt | 3' addition nt | 3' addition nt | 3' addition nt |
| hsa-miR-30c-1-3p | 3' trimmed | 3' trimmed | 3' trimmed | canonical | 3' trimmed |
| hsa-miR-1284 | 3' trimmed | 3' trimmed | 3' trimmed | 3' trimmed | canonical |
| hsa-miR-340-5p | 3' trimmed | canonical | canonical | canonical | canonical |
| hsa-miR-486-5p | 3' trimmed | canonical | canonical | canonical | canonical |
| hsa-miR-589-3p | 3' trimmed | canonical | 3' trimmed | canonical | canonical |
| hsa-miR-501-3p | 3' trimmed | 3' trimmed | canonical | 3' trimmed | canonical |
| hsa-miR-30d-5p | 3' trimmed | 3' addition t | 3' addition t | 3' addition t | 3' addition t |
| hsa-miR-1260b | canonical | canonical | 3' addition t | 3' addition t | 3' addition t |
| hsa-miR-21-5p | canonical | canonical | canonical | 3' addition t | canonical |
| hsa-miR-618 | 3' trimmed | canonical | 3' trimmed | canonical | 3' trimmed |
| hsa-miR-99b-5p | 3' trimmed | 3' trimmed | 3' trimmed | canonical | 3' trimmed |
| hsa-let-7e-3p | canonical | canonical | 3prime_U-form | canonical | canonical |
| hsa-let-7a-5p | 3' trimmed | canonical | canonical | canonical | canonical |
| hsa-miR-30d-3p | 3' trimmed | canonical | canonical | canonical | canonical |
| hsa-let-7d-5p | 3' trimmed | canonical | canonical | canonical | canonical |
| hsa-miR-4532 | 3' addition nt | 3' addition nt | 3' addition nt | 3' addition nt | 3' addition nt |
| hsa-miR-877-5p | 3' addition t | 3' addition nt | 3' addition nt | 3' addition nt | 3' addition nt |
| hsa-let-7i-5p | 3' trimmed | 3' trimmed | canonical | canonical | canonical |
| hsa-miR-548ap-5p | 5' addition t | 5' addition t | 3' addition t | 3' addition t | 5' addition t |
| hsa-let-7d-3p | 3' trimmed | canonical | canonical | canonical | canonical |
| hsa-miR-874-3p | 3' trimmed | canonical | canonical | canonical | canonical |
| hsa-miR-128-3p | 3' trimmed | 3' trimmed | 3' trimmed | canonical | 3' trimmed |
| hsa-miR-361-5p | 3' trimmed | canonical | 3' trimmed | canonical | canonical |
| hsa-let-7g-5p | 3' trimmed | 3' trimmed | canonical | 3' trimmed | 3' trimmed |
| hsa-miR-423-5p | 3' trimmed | 3' trimmed | canonical | canonical | canonical |
| hsa-miR-4435 | 3' trimmed | canonical | 3' addition nt | canonical | canonical |
| hsa-miR-616-5p | 3' trimmed | 3' trimmed | canonical | canonical | 3' trimmed |
| hsa-miR-26b-3p | 3' trimmed | 3' trimmed | 3' trimmed | canonical | 3' trimmed |
| hsa-miR-30a-5p | 3' trimmed | 3' addition t | 3' trimmed | 3' addition t | 3' trimmed |
| hsa-miR-4516 | 3' addition nt | 3' addition nt | 3' addition nt | 5prime_nt | 3' addition nt |
| hsa-miR-221-3p | 3' trimmed | 3' trimmed | 3' trimmed | 3' addition nt | 3' trimmed |
| hsa-miR-181a-2-3p | 3' trimmed | canonical | 3' trimmed | canonical | canonical |
| hsa-miR-625-5p | 3' trimmed | 3' trimmed | 3' trimmed | 3' addition t | 3' trimmed |
| hsa-miR-3615 | 3' trimmed | 3' addition t | 3' addition t | 3' addition t | 3' addition t |
| hsa-miR-5010-5p | canonical | 3' trimmed | canonical | canonical | canonical |
| hsa-miR-99a-3p | 3' trimmed | 3' addition t | 3' trimmed | 3' trimmed | 3' trimmed |
| hsa-miR-454-3p | canonical | canonical | 3' addition t | canonical | canonical |
| hsa-miR-122-5p | 3' trimmed | canonical | 3' trimmed | 3' trimmed | 3' trimmed |
| hsa-miR-411-5p | canonical | 5' addition t | canonical | 5' addition t | 5' addition t |
| hsa-miR-574-3p | 3' trimmed | canonical | canonical | canonical | canonical |
| hsa-miR-7848-3p | 3' trimmed | 3' trimmed | canonical | canonical | 3' trimmed |
| hsa-miR-144-3p | 3' trimmed | 3' trimmed | canonical | 3' trimmed | 3' trimmed |
| hsa-miR-194-5p | 3' trimmed | canonical | canonical | canonical | canonical |
| hsa-miR-3677-3p | 3' trimmed | 3' trimmed | 3' trimmed | 3' trimmed | canonical |
| hsa-miR-369-3p | 3' trimmed | canonical | canonical | canonical | canonical |
| hsa-miR-421 | 3' trimmed | 3' trimmed | 3' trimmed | canonical | 3' trimmed |
| hsa-miR-6803-3p | 3' trimmed | 3' trimmed | 3' trimmed | 3' addition t | 3' trimmed |
| hsa-miR-323a-3p | 5' addition t | canonical | canonical | 5' addition t | 5' addition t |
| hsa-miR-30a-3p | 3' trimmed | canonical | canonical | canonical | canonical |
| hsa-miR-744-3p | canonical | canonical | 3' trimmed | canonical | canonical |
| hsa-miR-576-3p | 3' addition t | 3' addition t | canonical | 3' addition t | 3' addition t |
| hsa-miR-1271-5p | 3' trimmed | 3' trimmed | canonical | canonical | canonical |
| hsa-miR-219a-1-3p | 3' addition t | 3' addition t | 3' addition t | 3' addition nt | 3' addition t |
