# Supplementary material for: Serum microRNA signatures as "liquid biopsies" for interrogating hepatotoxic mechanisms and liver pathogenesis in human
Source: PLoS One. 2017 May 17;12(5):e0177928. doi: 10.1371/journal.pone.0177928 (PMC5435338; doi:10.1371/journal.pone.0177928)
Supplement: S6 Table — List of small RNA like sequences that passed our criteria to be potential novel miRNAs. (DOCX) [file pone.0177928.s006.docx]

| sequence name | ID | miRDeep2 score | total read count | mature read count | Mature sequence | Precursor Sequence |
| --- | --- | --- | --- | --- | --- | --- |
| seq_1 | 1:1:249250621:1_2092 | 31.1 | 56 | 51 | cccuccgugcugcgucccucg | aggggcgcaggcgggagggcggcggggcugagcgcgcccuccgugcugcgucccucg |
| seq_2 | 1:1:249250621:1_2100 | 13.5 | 24 | 21 | auugaucagaaguccagccuaa | uugccuggaucauucugaucaagauggcuacagauacauaagcauugaucagaaguccagccuaa |
| seq_3 | 1:1:249250621:1_2767 | 25.8 | 47 | 35 | ucuccugcagcucugacuugc | ucuccugcagcucugacuugcguuuaauuacaagcaagucagagcugcaggagaga |
| seq_4 | 1:1:249250621:1_351 | 43.4 | 84 | 70 | guggcagacugugagggaaau | uuucccucccuggcugccguccacacaucguugguggcagacugugagggaaau |
| seq_5 | 1:1:249250621:1_3586 | 816.4 | 1598 | 1199 | ccucuccuaaccucgcucucg | agagucgagagugggagaagagcggagcgugugagcaguacugcggccuccucuccucuccuaaccucgcucucg |
| seq_6 | 1:1:249250621:1_677 | 110.1 | 212 | 168 | agccucccagucuggccugagu | ucaggccaggcugggaggaugcuguaaaugcagccucccagucuggccugagu |
| seq_7 | 1:1:249250621:1_684 | 24.1 | 44 | 35 | ucuccugcagcucugacuugc | ucuccugcagcucugacuugcuuguaauuaaacgcaagucagagcugcaggagaga |
| seq_8 | 10:1:135534747:1_25371 | 52.6 | 99 | 95 | caugguccauuuugcucugcuuc | caugguccauuuugcucugcuucugaaagcugaguacuuugcaagcagagcaaaacggaccaugcau |
| seq_9 | 10:1:135534747:1_25429 | 33.6 | 63 | 62 | accugaaagugaucugugau | accugaaagugaucugugauccaaccucuucuggaucacagaucacuuucagaaa |
| seq_10 | 12:1:133851895:1_28718 | 652.7 | 1279 | 1260 | aaagcaaauguugggugaacggcu | aaagcaaauguugggugaacggcuguuuccucuuauucaagccaugcaccuuacucuugcug |
| seq_11 | 12:1:133851895:1_29749 | 63.4 | 121 | 119 | ucuggcuccuuucuaaucacu | ucuggcuccuuucuaaucacuaugucaggaugccacagugauuagacaggagccagacaca |
| seq_12 | 12:1:133851895:1_29863 | 23.4 | 43 | 41 | cgaggggucuuccaggaacu | cgaggggucuuccaggaacuccggcaucucggggaucugcgaagcccccuccccaca |
| seq_13 | 13:1:115169878:1_30273 | 101.4 | 195 | 182 | uaugugccuaguggcugcugucu | agaguagccacuagccacaugucaguucaugcuuuuaaggcuauaugugccuaguggcugcugucu |
| seq_14 | 14:1:107349540:1_31731 | 17.6 | 31 | 27 | agcaaggcggcaucucucuccu | cagagagaugccgccuugcuccuccuguucaaggagcaaggcggcaucucucuccu |
| seq_15 | 16:1:90354753:1_34462 | 13.5 | 22 | 18 | ccuccggaagcuccgccccacg | ccuccggaagcuccgccccacgcguucccggggcgcaugcgacguggggcggagcgucuggaagc |
| seq_16 | 16:1:90354753:1_35569 | 76.1 | 144 | 118 | cggcggcuccagggaccuggcg | cggcggcuccagggaccuggcggccgccgaucggggcugcgaggccccauggcgccgcc |
| seq_17 | 17:1:81195210:1_36139 | 48.8 | 92 | 91 | cucagggccugcucccaccccgc | uggggugggaggggugaugagccccuguuaucugaggagcucagggccugcucccaccccgc |
| seq_18 | 17:1:81195210:1_36871 | 59.6 | 114 | 109 | ucaggcuuugcaucccgggacg | cucccgggauccgaagcuggggagcugcuucuguccaaucaggcuuugcaucccgggacg |
| seq_19 | 17:1:81195210:1_36883 | 16 | 28 | 24 | uuucuagccucucugccaagu | ucuggcagagaggcccagaauccucccaagguguagguuucuagccucucugccaagu |
| seq_20 | 17:1:81195210:1_36914 | 79.4 | 153 | 137 | uagcaccugccgagcacugaga | uagcaccugccgagcacugagaacaaggaagcccucagugcacguuagcugucauu |
| seq_21 | 17:1:81195210:1_37065 | 107.1 | 207 | 182 | ccucaguccguauuggucucu | ccucaguccguauuggucucuauggcauccauagaggccauucggcucugaggucc |
| seq_22 | 18:1:78077248:1_38413 | 639 | 1252 | 1177 | cuucgaaagcggcuucggcu | cuucgaaagcggcuucggcugccucggccgcuuucgaagcc |
| seq_23 | 19:1:59128983:1_39021 | 45.6 | 86 | 85 | uaucugcuguuguccccucagg | cagaggccgcacagcaggaaccucacauuggauguaucugcuguuguccccucagg |
| seq_24 | 19:1:59128983:1_39101 | 101.3 | 196 | 195 | cuuccccacccucuccugcag | ggggagaggaggggaccagggcugugaauuaccuucccuuccccacccucuccugcag |
| seq_25 | 19:1:59128983:1_39170 | 33.7 | 62 | 61 | gugugugcaccugugucugucugu | gugugugcaccugugucugucuguauucguguuguauagacagacgcagguacacaca |
| seq_26 | 19:1:59128983:1_39331 | 11.7 | 21 | 14 | ucugcccuccuacuccccagg | acgggauggaggaacagagaaagcuaaggguagccucugcccuccuacuccccagg |
| seq_27 | 19:1:59128983:1_39734 | 110.6 | 213 | 211 | ucagaaccgaccgccucccagc | ggggguggcggagguuucugagaagugacauaggcugggaugacucagaaccgaccgccucccagc |
| seq_28 | 2:1:243199373:1_4466 | 47.1 | 90 | 79 | ccagccucagucucuguauccu | ccagccucagucucuguauccugagauuuugccagauagguuauagaaacugaggcucuuccgg |
| seq_29 | 2:1:243199373:1_5347 | 22.6 | 41 | 38 | acacaagugggccaaagagcu | ucuuugccccaccugugucgagauaagcucgacacaagugggccaaagagcu |
| seq_30 | 2:1:243199373:1_6526 | 23 | 41 | 38 | agcuccugacauugucaccuga | aggugacaaugucaggugcucaggugcucaggagcuccugacauugucaccuga |
| seq_31 | 2:1:243199373:1_6928 | 14.4 | 25 | 21 | aauagugucuagaauaucuuga | aagauauucuagacacuauucugugucacccagaauagugucuagaauaucuuga |
| seq_32 | 20:1:63025520:1_41355 | 73.4 | 144 | 86 | cccaggacaguuucagugaug | cccaggacaguuucagugaugcaaauugugugcccucugguucagcugaaacaguccuggacu |
| seq_33 | 3:1:198022430:1_8020 | 813.3 | 1592 | 1582 | ggacgaaauccaagcgcagcug | ggacgaaauccaagcgcagcuggaaugcucuggagacaacagcugcuuuugggauuccguugccc |
| seq_34 | 3:1:198022430:1_8052 | 19.3 | 34 | 23 | ggcucaagguucaagaaggc | ggcucaagguucaagaaggccaauguugugagcacaggccuucucgagccuugagugugc |
| seq_35 | 3:1:198022430:1_8963 | 33.2 | 61 | 60 | ucaguaaguggcacucugucu | gacagagugccacuuacugagagaaaaaaccuuucaguaaguggcacucugucu |
| seq_36 | 4:1:191154276:1_11673 | 21 | 37 | 21 | agauggggagaacucaauccu | agauggggagaacucaauccuaaauguauuuaggaauuuaagauugaguucuccccaucu |
| seq_37 | 5:1:180915260:1_14775 | 23 | 43 | 31 | uucuuggaccuugcuucagacc | uucuuggaccuugcuucagacccacugaaucagaagcucuggagugggacccagugaucu |
| seq_38 | 5:1:180915260:1_14832 | 62.4 | 119 | 114 | cugccaucuggugccagccuuu | ggcuggccuggagggcaggccagagauuggggcugccaucuggugccagccuuu |
| seq_39 | 6:1:171115067:1_15290 | 20.6 | 36 | 34 | agaauaugggagucuguggcu | agaauaugggagucuguggcuauuaaaagacaaaauagccacagacucccauauucuca |
| seq_40 | 6:1:171115067:1_16623 | 20.6 | 36 | 34 | agaauaugggagucuguggcu | agaauaugggagucuguggcuauuuugucuuuuaauagccacagacucccauauucuca |
| seq_41 | 6:1:171115067:1_17014 | 22.9 | 48 | 18 | aucugggucugcgugaguaac | aucugggucugcgugaguaacuuucuugcauaaucaagguuacucaaguagaagccugaaa |
| seq_42 | 7:1:159138663:1_19288 | 38.7 | 73 | 54 | aucauuuaugcuugcggaggac | gaccguaaggauacaaugauugagaugcaauguaagugcugcaaucauuuaugcuugcggaggac |
| seq_43 | 7:1:159138663:1_19665 | 3836.4 | 7523 | 7520 | ucggcuguguaucucugugcc | ucggcuguguaucucugugccagaagcuuagccacaguguggcacagucguguc |
| seq_44 | 8:1:146364022:1_21464 | 35.9 | 66 | 65 | uuauccuccaguagacuaggga | ccagccuacuggaggauaagaggauauaaaggucucuuauccuccaguagacuaggga |
| seq_45 | 9:1:141213431:1_23438 | 22.9 | 41 | 38 | cggggcacucgggucuuugcug | ugcugggacucgggcucccagggucugcucggucucucucggggcacucgggucuuugcug |
| seq_46 | X:1:155270560:1_43419 | 17.2 | 30 | 23 | auuuaggccuguauaaucaugg | augauuauacaggccuaaauguuuggcaagaaauauuuaggccuguauaaucaugg |
| seq_47 | X:1:155270560:1_44370 | 18.4 | 33 | 30 | ccccccggagcggcccugaga | aucggggaccaccuccggggcugccuccgguucuugaggccccccggagcggcccugaga |
