# Supplementary material for: Serum microRNA signatures as "liquid biopsies" for interrogating hepatotoxic mechanisms and liver pathogenesis in human
Source: PLoS One. 2017 May 17;12(5):e0177928. doi: 10.1371/journal.pone.0177928 (PMC5435338; doi:10.1371/journal.pone.0177928)
Supplement: S7 Table — List of small RNA like sequences that passed our criteria to be potential novel miRNAs and are altered upon liver disease and T2DM. (DOCX) [file pone.0177928.s007.docx]

|  | Disease | FC | FDR |
| --- | --- | --- | --- |
| seq_22 | APAP | 1.3607224 | 2.92E-02 |
| seq_22 | LC | 1.4573146 | 2.40E-02 |
| seq_10 | T2DM | 1.592579 | 0.006114101 |
| seq_16 | T2DM | -1.984888 | 0.037386559 |
| seq_24 | T2DM | -2.770174 | 0.001716393 |
| seq_5 | T2DM | -2.77875 | 0.000101163 |
| seq_5 | LC | 1.643792 | 0.02402743 |
| seq_43 | T2DM | 1.501768 | 0.006131583 |
